# Computational and Experimental Study of Turbo‐Organomagnesium Amide Reagents: Cubane Aggregates as Reactive Intermediates in Pummerer Coupling

**DOI:** 10.1002/chem.202004164

**Published:** 2021-01-12

**Authors:** Ferran Planas, Stefanie V. Kohlhepp, Genping Huang, Abraham Mendoza, Fahmi Himo

**Affiliations:** ^1^ Department of Organic Chemistry Arrhenius Laboratory Stockholm University 10691 Stockholm Sweden; ^2^ Department of Chemistry School of Science Tianjin University Tianjin 300072 P. R. China

**Keywords:** computational chemistry, density functional calculations, Grignard reaction, isotope effects, reaction mechanism

## Abstract

The dynamic equilibria of organomagnesium reagents are known to be very complex, and the relative reactivity of their components is poorly understood. Herein, a combination of DFT calculations and kinetic experiments is employed to investigate the detailed reaction mechanism of the Pummerer coupling between sulfoxides and turbo‐organomagnesium amides. Among the various aggregates studied, unprecedented heterometallic open cubane structures are demonstrated to yield favorable barriers through a concerted anion‐anion coupling/ S−O cleavage step. Beyond a structural curiosity, these results introduce open cubane organometallics as key reactive intermediates in turbo‐organomagnesium amide mixtures.

## Graphical abstract

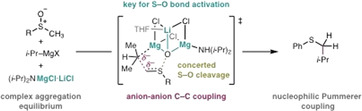

## Introduction

**Scheme 1:**
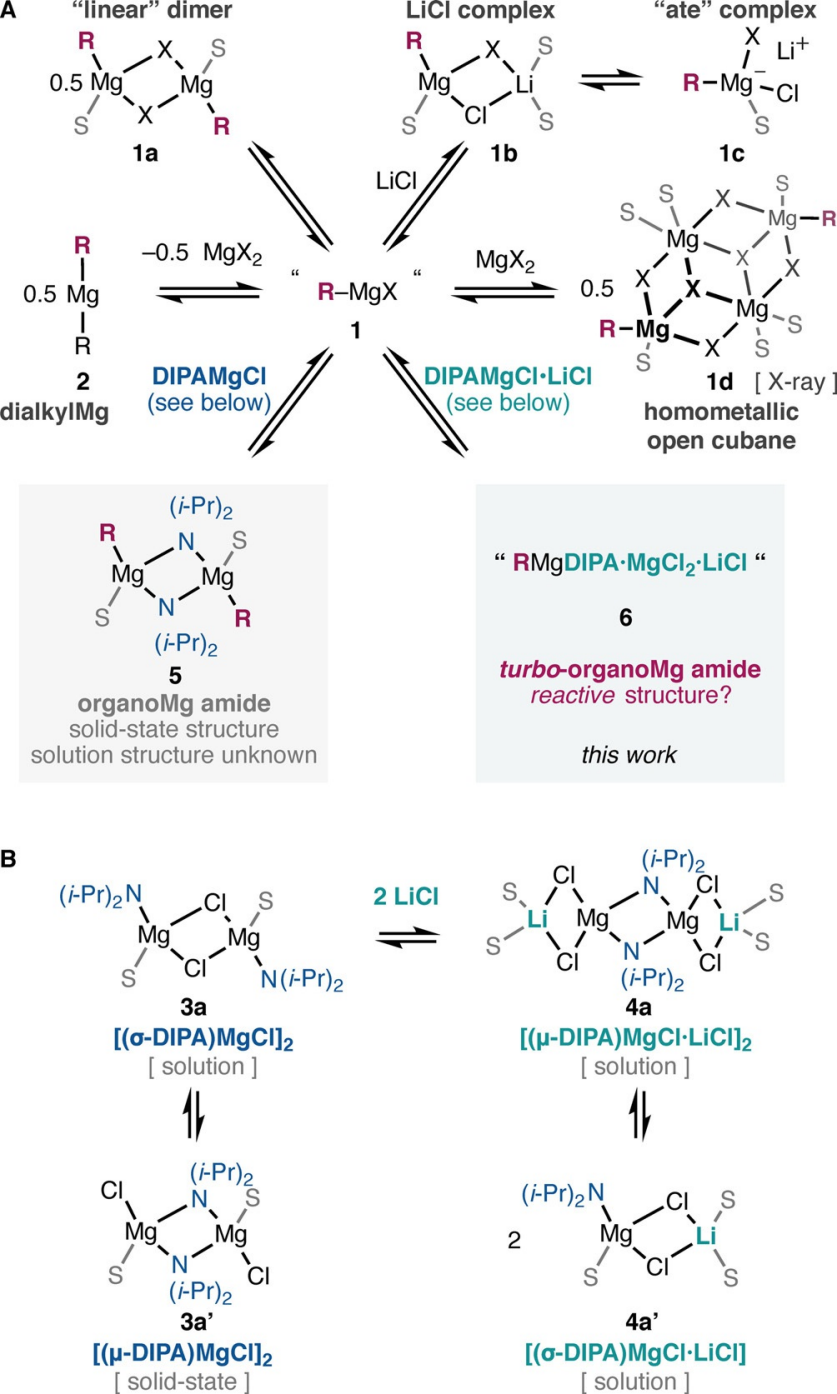
Relevant structures involved in the solution equilibria of Grignard reagents and their organomagnesium amides (A), or Hauser and Knochel–Hauser bases (B). For simplicity, only the most common dominant species are shown. DIPA, diisopropylamide; S, solvent.

Main group organometallics are the most common source of nucleophilic carbon in organic synthesis. Grignard reagents (R‐MgX; **1**) have been particularly instrumental due to their balanced reactivity, cost, and functional group tolerance.^[1]^ Structural studies in the solid‐state and solution dynamics of these seemingly simple organometallics have demonstrated the complexity of their aggregation equilibria, often involving various species co‐existing in solution (Scheme 1 A).[^2^, ^3^] The Schlenk‐equilibrium yields diorganomagnesium species **2** and it is an important example of this dynamic behavior, which is influenced by concentration, solvent, steric properties of the carbon fragment, halide anion, and temperature.[^4^, ^5^] The presence of magnesium or lithium salts fundamentally changes the reactivity of Grignard reagents and affects the positions of these equilibria through the formation of mono‐ or multinuclear complexes with variable relative stabilities and reactivities (i.e. “linear” dimers **1 a**, LiCl adducts **1 b**, “ate” complexes **1 c**, open cubane aggregates **1 d**, etc.).[^3^, ^6^] Despite the studies on the solution equilibria of these systems to determine the most abundant species in different conditions, their relative contributions to the overall reactivity is difficult to assess. This is due to the problematic deconvolution of the roles that various aggregates take in the overall kinetic progress of the reaction.

The solution equilibria of magnesium amide bases (R_2_N‐MgX), termed ‘Hauser’ bases **3** (Scheme 1 B),^[7]^ and their LiCl complexes, known as ‘Knochel–Hauser’ or ‘*turbo*‐Hauser’ bases **4**, are governed by similar principles.[^8^, ^9^] These reagents have been mostly used in the selective magnesiation of C−H bonds.^[10]^ They are structurally diverse and the amide ligand can occupy bridging or terminal positions.[^8^, ^9^, ^11^, ^12^, ^13^] Hauser bases have been combined with Grignard reagents to drive the most challenging deprotonations. The resulting organomagnesium amides (**5**; R‐Mg‐NR_2_) turn the acid‐base reaction irreversible through deprotonation of the initially formed amine with the magnesium alkyl.^[14]^ The solution dynamics of these reagents are significantly more complex than their parent components. The Schlenk (diorganomagnesium and magnesium halide clusters) and aggregation (monomeric, dimeric, etc.) equilibria on the Grignard and Hauser base co‐exist with various heteroleptic organomagnesium amide complexes. Understandably, the structural information on these systems is limited to solid‐state studies, in which dimeric structures with bridging amide ligands are most common (see **5**, Scheme 1 A).^[15]^

**Scheme 2:**
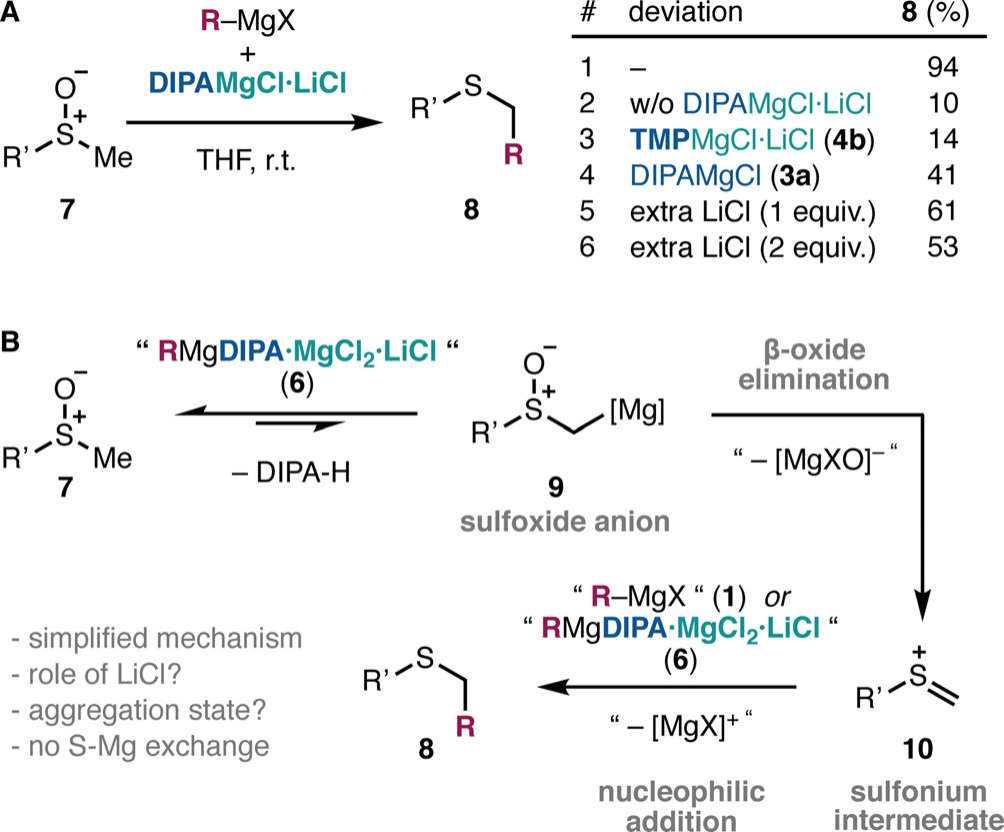
(A) Pummerer coupling between sulfoxides and Grignard reagents **1** mediated by the turbo‐Hauser base DIPAMgCl⋅LiCl (**4 a**) including key optimization studies.^[16]^ (B) Previous mechanistic proposal.

Recently, Mendoza and co‐workers discovered the differential reactivity of Grignard reagents **1** upon the addition of a specific Knochel–Hauser base (DIPAMgCl⋅LiCl; **4 a**) in the context of Pummerer‐type reactions (Scheme 2 A).^[16]^ This process allows the direct transformation of sulfoxides **7** into α‐functionalized sulfides **8**. Unlike conventional electrophilic Pummerer reactions, this method is compatible with strong and localized alkyl‐, aryl‐, vinyl‐ and alkynyl‐Grignard nucleophiles. This work introduced the potential of *turbo*‐magnesium amides as activators of organometallics, beyond their role as bases in earlier work.[^10^, ^14^] Surprisingly, the mixture of Grignard **1** and DIPAMgCl⋅LiCl (**4 a**) eluded the fast S−Mg exchange that occurs between Grignards **1** and sulfoxides **7** at cryogenic temperatures.^[17]^ Interestingly, control experiments without **4 a** (entries 1 and 2; Scheme 2 A) or with a similar Knochel–Hauser base **4 b** (entry 3) clearly indicated the importance of the diisopropylamide fragment,^[8]^ which pointed to a critical aggregation of Grignard **1** with the base **4 a** into a new *turbo*‐organomagnesium amide species **6**. It was also found that LiCl (entry 4), and in particular the 1:1:1 stoichiometry between the Grignard, the base and LiCl, are critical for the success of this reaction (entries 5 and 6). Moreover, it was recently found that the same Grignard‐DIPAMgCl⋅LiCl combination is uniquely more reactive and more selective in addition reactions to challenging carboxylate anions.^[18]^ This further suggests that a new species is formed in solution upon mixing Grignard reagents **1** and DIPAMgCl⋅LiCl (**4 a**), and this is relevant for the new reactivity observed.

Although the exact mechanism of the Grignard activation by DIPAMgCl⋅LiCl (**4 a**) in this process was unclear, the initially proposed mechanism was consistent with previous knowledge on related β‐functionalized organolithiums^[19]^ and Pummerer reactions (Scheme 2 B).^[20]^ The deprotonation of the sulfoxide **7** would produce a sulfoxide anion **9**, which would undergo S−O bond excision through β‐oxide elimination^[19]^ to generate a Pummerer sulfonium intermediate **10**.^[20]^ The latter would react with an undefined nucleophilic Grignard species **1,6** to generate the thioether product **8**.

To gain insight into the intimate mechanism of activation of the Grignard reagent **1** bestowed by the Knochel–Hauser base **4 a**, we set out to undertake a computational and kinetic profiling of this system. At the onset, it is important to underscore that only solid‐state information existed on some organomagnesium amides **5** ^[15]^ and none on the LiCl adducts **6** that seemed to be responsible for the differential reactivity observed (see Scheme 2 A). Given the complex equilibria that may be at place in solutions of *turbo*‐organomagnesium amides **6**, the combined experimental‐computational approach allows to evaluate the energies of various aggregates, as well as their relative relevance for the reactivity towards sulfoxides **7**.

## Results and Discussion

In both the experimental and computational investigations described below, the reaction was studied using methyl phenyl sulfoxide (**7 a**) and isopropylmagnesium chloride (**1 e**) as representative reactants.

**Scheme 3:**
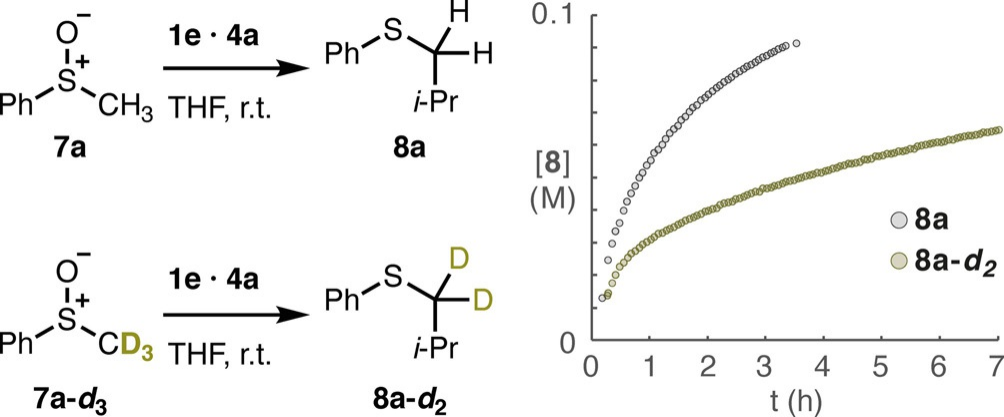
The slower reaction with a deuterated substrate **7 a**–**d_3_** is consistent with the rate‐determining deprotonation predicted by the calculations.

First, we set out to observe the evolution of the system using in situ no‐D ^1^H‐NMR spectroscopy. The sulfoxide **7 a** displayed broadened and shifted resonances when the base DIPAMgCl⋅LiCl **4 a** and Grignard **1 e** were subsequently added, thus suggesting interaction through several complexes in equilibrium (see Supporting Information). This mixture is unstable and evolves into the product even at low temperature, thus preventing structural studies on this equilibrium by NMR or XRD. As discussed above, the dominant structures participating in this rapid equilibrium may not be relevant for the reactivity, and the reactive species may be minor components. The formation of the product could be monitored by NMR, finding that the reaction reached 91 % yield in about 3 h at 25 °C (Scheme 3).

Next, we employed density functional theory (DFT) calculations, in the form of the dispersion‐corrected B3LYP method,[^21^, ^22^] to determine the structures of the most stable species that can form initially under the experimental conditions. To that end, we optimized the structures of various aggregates that can form prior to the addition of the sulfoxide, and compared their energies to those of the monomer of the magnesium amide and the dimer of the Grignard reagent, as these two have been reported to be dominant forms of the respective reagents in solution.[^4b^, ^8^] This study concluded that the aggregation of the Grignard reagent and DIPAMgCl⋅LiCl results in more stable heteroleptic aggregates (see Supporting Information for details).

**Figure 1:**
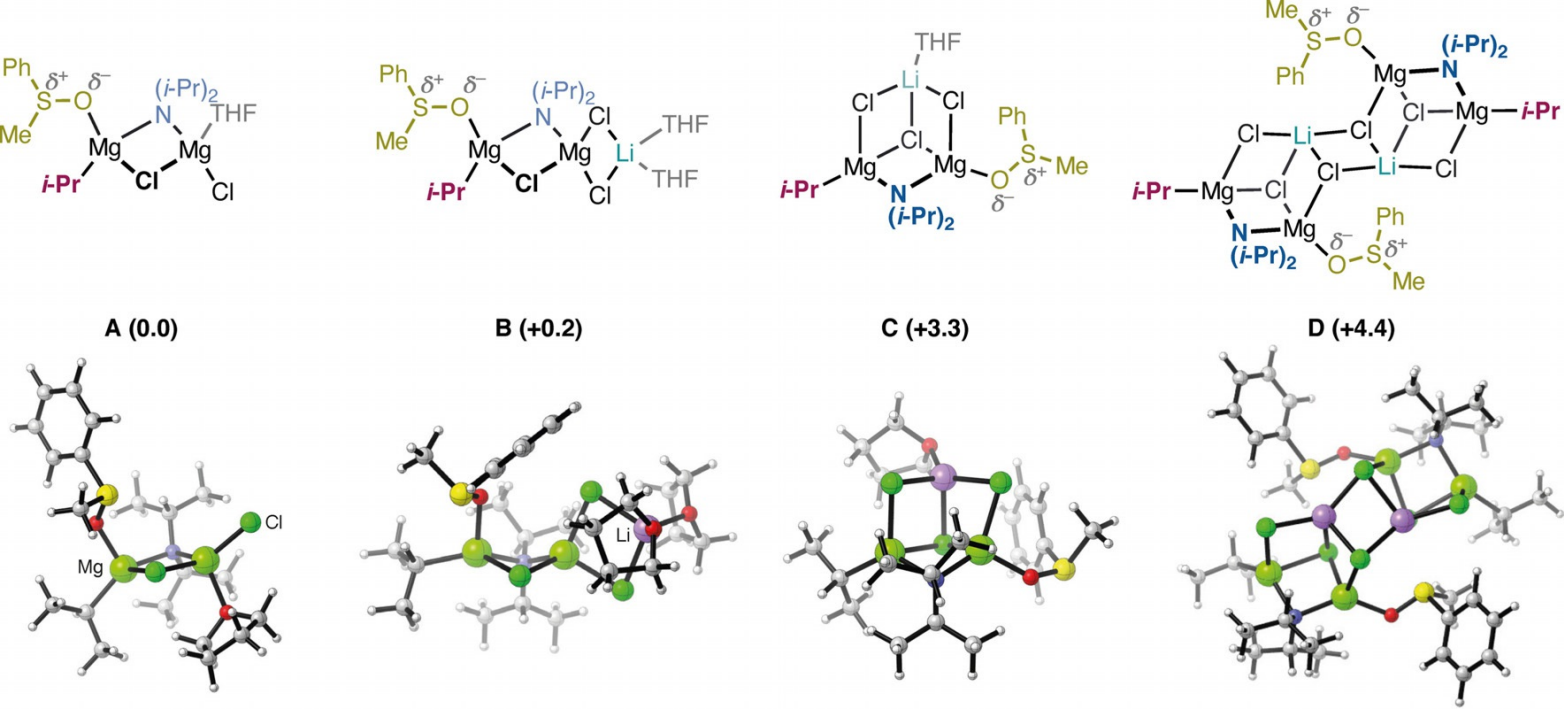
Structures of the four starting complexes used to study the reaction mechanism. Relative energies are indicated in kcal mol^−1^. For important distances in these structures, see Supporting Information.

The two most stable complexes with linear topology and two open cubane complexes were then used to explore the coordination of the sulfoxide substrate. In all cases, the exchange of one solvent molecule for the sulfoxide at the terminal magnesium resulted in complexes that are 3–6 kcal mol^−1^ more stable (see Supporting Information). The exothermic character of this ligand substitution is consistent with the interaction observed by ^1^H‐NMR in the reaction mixture (see above). The resulting complexes **A–D** are shown in Figure 1 along with their calculated relative energies.

Complex **A** consists of two magnesium ions bridged by the amide and a chloride ion. One magnesium ion binds the sulfoxide and the isopropyl moiety, while the other ion binds a second chloride and a THF molecule. Complex **B** is essentially the lithium chloride adduct of complex **A**, where two chlorides bridge the Mg and the Li ions. This structural motif is commonly found in related Grignard and Hauser‐base complexes.[^8^, ^9^, ^10^, ^12^] The energy of complex **B** is only 0.2 kcal mol^−1^ higher than complex **A**.

Despite not being reported in structural studies, we explored the feasibility of the magnesium‐lithium heterometallic open cubanes **C** and **D** as reactive intermediates. Similar cubane and open cubane structures have been implicated in unrelated reactions involving potassium^[23]^ or zinc aggregates.^[24]^ Complex **C** features two magnesium ions linked by the amide and a chloride, with additional chloride bridges with the lithium ion to complete the compact structure. This species is calculated to be +3.3 kcal mol^−1^ relative to complex **A**. Complex **D** is a dimer formed of two units of complex **C**. This aggregate is calculated to be only 4.4 kcal mol^−1^ higher than complex **A**. As seen in Figure 1, in complexes **A–D** the amide bridges two magnesium ions while the sulfoxide and alkyl ligand bind to a single magnesium. Exchanging of the positions of the amide and sulfoxide leads to higher energies (see Supporting Information).

Next, we calculated the full reaction mechanisms starting from these four complexes. The calculations show that they follow essentially the same reaction mechanism, consisting of the following steps: 1) a ligand rearrangement into a *μ*‐sulfoxide/*σ*‐amide disposition, 2) a proton transfer from the sulfoxide α‐carbon to the amide, forming a sulfoxide enolate featuring an S=C double bond, and finally 3) a nucleophilic addition of the isopropyl moiety to the sp^2^ carbon of the sulfoxide anion taking place concertedly with the cleavage of the S−O bond. The latter of these steps is a surprising anion‐anion C−C coupling that contrasts with electrophilic Pummerer reactions via sulfonium intermediates **10**.^[20]^

**Figure 2:**
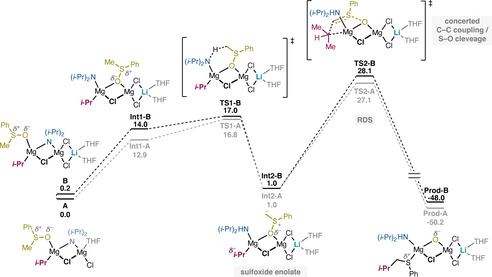
Free‐energy profile of the reaction mechanism of complexes **A** and **B**. Relative energies are in kcal mol^−1^.

As shown in Figure 2, the calculations demonstrate that the two linear complexes **A** and **B** have very similar reaction energy profiles. The optimized geometries of the intermediates and transition states (TSs) for both complexes are given in the Supporting Information. The last step was found to be rate‐determining for both cases, with a calculated barrier of 27.1 kcal mol^−1^ for complex **A** and 28.1 kcal mol^−1^ for complex **B**. An important observation here is that, in contrast to previous proposals,^[16a]^ the β‐oxide elimination pathway^[19]^ to form a discrete sulfonium intermediate^[20]^ could not be obtained by the calculations (see Supporting Information for details).

The overall barriers calculated for these two complexes are not compatible with the reaction time and temperature used in the experiments, indicating that the two considered linear complexes might not be the active species in the reaction. Furthermore, the fact that identical mechanisms were obtained for complexes **A** and **B**, with very similar barriers, suggests that other linear complexes (e.g. complexes **E–K** in the Supporting Information) are also likely to behave similarly, yielding high barriers. These complexes were therefore not considered further by the calculations.

**Figure 3:**
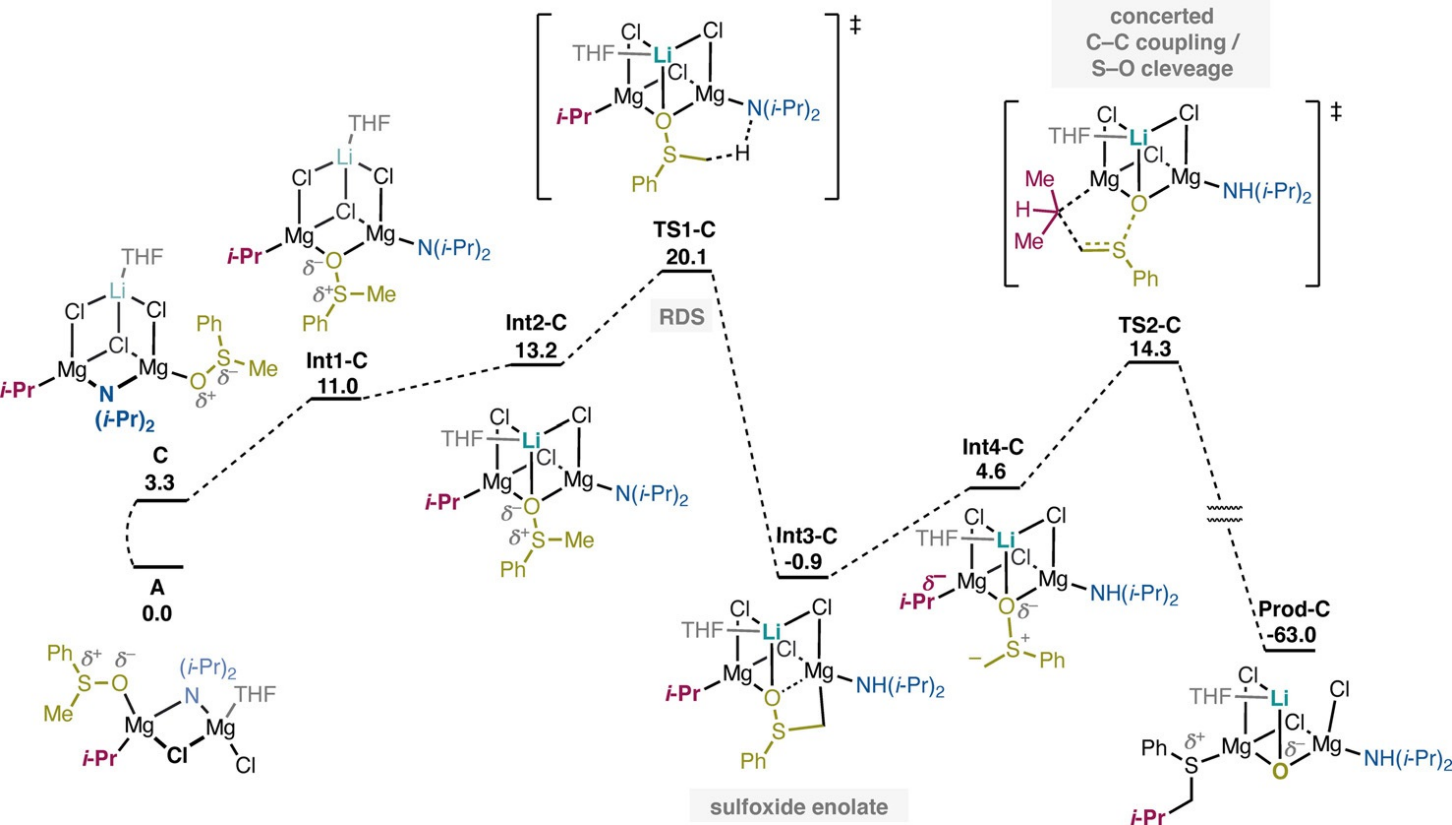
Free‐energy profile of the reaction mechanism of complex **C**. Relative energies are in kcal mol^−1^.

**Figure 4:**
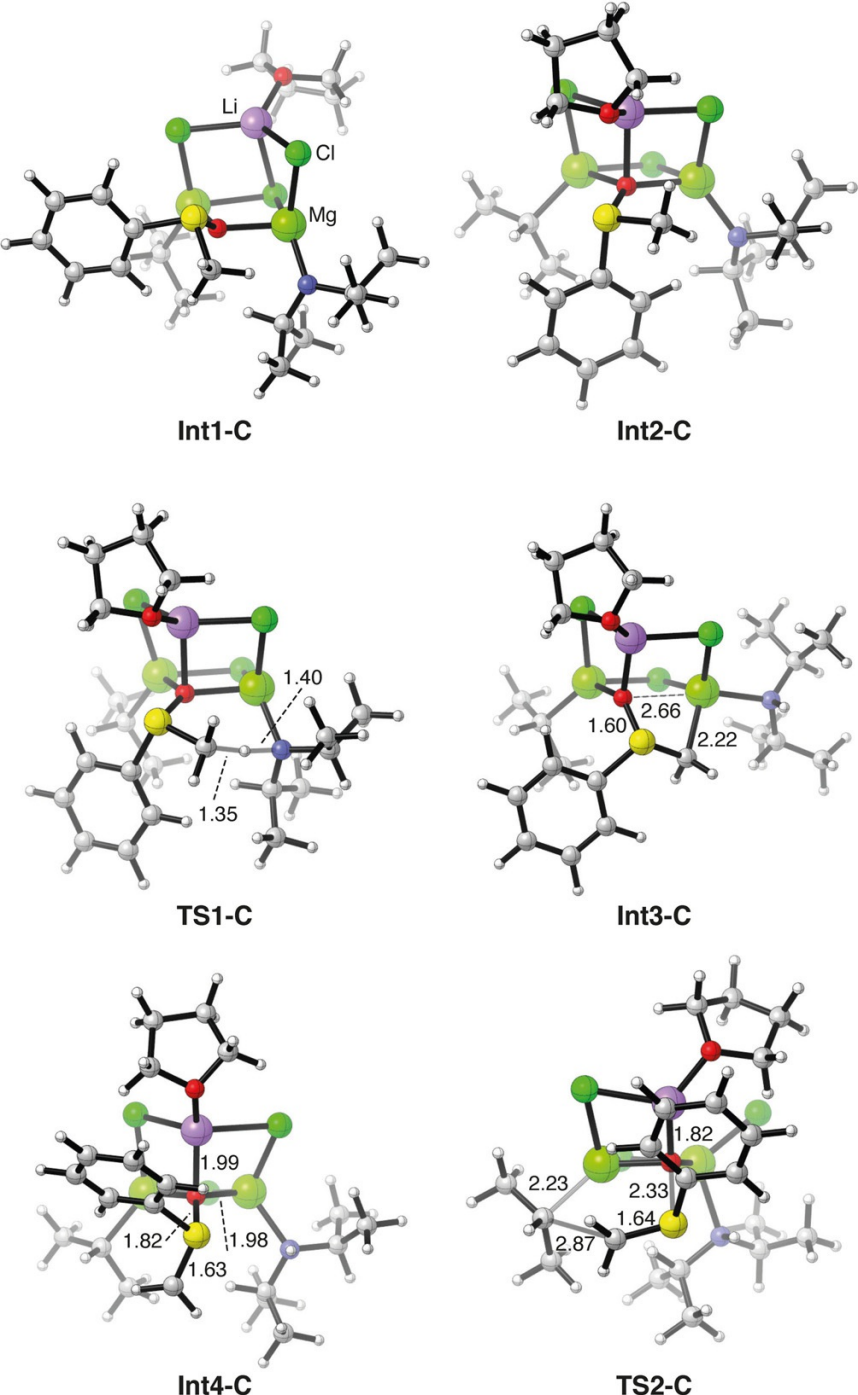
Optimized geometries of the intermediates and transition states of the reaction mechanism for complex **C**. Selected distances are given in Angstrom.

We then turned our attention to reactions starting from the open cubane structures **C** and **D**. These two complexes gave similar energy profiles compared to each other, but very different compared to the linear structures discussed above. The calculated energy graph and the optimized geometries of the intermediates and transition states for the reaction starting from complex **C** are depicted in Figures 3 and 4, while the corresponding figures for complex **D** are given in the Supporting Information.

According to this mechanism, the first step also here is the formation of the intermediate with the sulfoxide in the bridging position and the amide in the terminal one (σ‐amide; **Int1‐C**). The energy of this intermediate is 7.7 kcal mol^−1^ higher than complex **C**, that is, +11.0 kcal mol^−1^ compared to the most stable complex **A**. This rearrangement is necessary, as the direct deprotonation of the sulfoxide in the terminal position is associated with high barriers (see Supporting Information), probably due to the coordinative saturation of the bridged amide (μ‐amide) that prevents the proton abstraction. Next, **Int1‐C** undergoes a second rearrangement step to form **Int2‐C**, 2.2 kcal mol^−1^ higher in energy, in which the oxygen of the sulfoxide is bound to the three metallic ions (see Figures 3 and 4). **Int2‐C** can then undergo the intramolecular proton transfer through **TS1‐C**, which has an accumulated barrier of 16.8 kcal mol^−1^ relative to complex **C**, that is, 20.1 kcal mol^−1^ relative to the most stable species, complex **A**. Interestingly, in the resulting **Int3‐C** (Figure 4), the anionic carbon of the sulfoxide enolate binds to the Mg ion (Mg−C distance 2.22 Å), displacing the oxygen, which is no longer bridging (Mg‐O distance 2.66 Å). At **Int2‐C**, the alternative proton transfer to the isopropyl moiety has a more than 11 kcal mol^−1^ higher barrier compared to **TS1‐C** and can be ruled out (see Supporting Information).

**Int3‐C** is calculated to be 14.1 kcal mol^−1^ lower than **Int2‐C** (−0.9 kcal mol^−1^ relative to complex **A**), and in order for the reaction to proceed, another rearrangement step is necessary, namely the sulfoxide rotates around the S−O bond to form **Int4‐C**, which is calculated to be 5.5 kcal mol^−1^ higher than **Int3‐C**. This rotation breaks the Mg‐C coordination, resulting in an sp^2^‐hybridized anion in close proximity with the isopropyl moiety (**Int4‐C**). In the final step of the reaction, the isopropyl adds to the sulfoxide enolate concertedly with the cleavage of the S−O bond to form the final product, similarly to the mechanism found for the linear complexes **A** and **B**. The cumulative barrier for this step is calculated to be 15.2 kcal mol^−1^ relative to **Int3‐C** (see Figure 3). In the transition state for the C−C bond formation (**TS2‐C**), the Li−O distance is 1.82 Å, which is 0.17 Å shorter than in the preceding intermediate due to the increasing negative charge on the oxygen. This shorter distance, in turn, increases the congestion in the complex, which results in the chloride that is bridging the Li and Mg ions becoming terminal.

The rate‐determining barrier of this mechanism is thus 20.1 kcal mol^−1^, which is 7–8 kcal mol^−1^ lower than the barriers obtained for the linear complexes **A** and **B**. Interestingly, the energies for the reaction starting from the double cubane complex **D** are similar to those of complex **C** (details given in Supporting Information), with a calculated rate‐determining barrier of 24.9 kcal mol^−1^. Hence, despite the fact that the cubane starting structures are slightly higher in energy compared to the linear ones (Figure 1), they lead to reaction mechanisms with lower barriers. One reason is the greater extent of electrostatic stabilization of the negative charge of the oxygen upon cleavage of the S−O bond due to the complexation of the lithium ion. This stabilization can be detected from the shortening of the Li−O distance at the last step, and also from the much lower energy of the final product, which is calculated to be much more stable than the products of the linear complexes (compare, for example, **Prod‐C** in Figure 3 with **Prod‐A** in Figure 2).

It is also interesting to note that the S−O bond distance of the sulfoxide enolate of **Int4‐C** is 0.10 Å longer than the corresponding distance for complex **B**. Moreover, the sp^2^ carbon in **Int4‐C** has less negative charge (−0.82) compared to the corresponding intermediate for complex **B** (−0.97), indicating that the carbon in **Int4‐C** would facilitate coupling with the alkyl nucleophile in the case of the open cubane structure (see Supporting Information for details).

An important difference between the reactions of the linear complexes and the cubane ones is that the C−C bond formation is rate‐limiting in the former, while in the latter the proton transfer step is rate‐limiting. The fact that this step is also irreversible implies that one would expect to observe a primary kinetic isotope effect (KIE) when using an analogous deuterated substrate if the predictions of the calculations are correct. Indeed, we observed a significantly slower reaction using sulfoxide **7 a**–***d***_**3**_, reaching only 48 % yield in the first 3.3 h as evidenced by ^1^H‐NMR (Scheme 3). This contrasts with the complete conversion in the same time obtained with the protonated substrate **7 a**. However, the complex kinetics observed in the system prevented an accurate determination of the KIE value. The deprotonation equilibrium could also affect the KIE observed, but the irreversible deprotonation predicted by the calculations makes this scenario less likely. The mechanism involving chiral cubane intermediates presented herein may also explain the partial chirality transfer observed for more complex substrates,^[16a]^ which was initially ascribed to an intimate sulfonium ion pair in line with earlier Pummerer literature.

## Conclusions

In summary, the Pummerer reaction between sulfoxides and Grignard nucleophiles activated by *turbo*‐Hauser bases has been calculated to occur through an open‐cubane heterometallic *turbo*‐organomagnesium amide. The caged topology of this intermediate is crucial to facilitate the activation of the S−O bond and stabilize the negative charge on the sulfoxide enolate carbon. Surprisingly, the formation of the new C−C bond occurs through a concerted anion‐anion coupling with concomitant cleavage of the S−O bond. This contrasts with conventional Pummerer‐type reactions operating through electrophilic intermediates. These insights explain the critical role of LiCl in nucleophilic Pummerer coupling, and open new avenues for research in new synthetic methods based on the synergy between Knochel–Hauser bases and Grignard reagents.

## Computational Methods

All the calculations were performed with the package Gaussian 09^[25]^ and the B3LYP‐D3(BJ) functional.[^21^, ^22^] Geometry optimizations and frequency calculations were carried out with the 6‐31G(*d,p*) basis set. Solvation effects were considered by performing single‐point calculations with the SMD model and THF as solvent.^[26]^ To obtain better accuracy, the electronic energies of the optimized structures were calculated using single‐point calculations with the larger basis set 6–311+G(2*d,2p*). Vibrational frequencies were calculated at the same level of theory as the geometry optimization, and the Gibbs free energy corrections were calculated using the rigid‐rotor‐harmonic‐oscillator (RRHO) approximation at room temperature. Standard state corrections to account for the conversion from the 1 atm ideal gas to the 1 m standard state for the solutes and the 12.3 m for the solvent were included. This correction was done by adding the term *RT* ln(24.5)=+1.9 kcal mol^−1^ for the solutes and *RT* ln(24.5⋅12.3)=+3.4 kcal mol^−1^ for the solvent.

### Supporting information

Further computational results, absolute energies and energy corrections, and Cartesian coordinates of reported structures (PDF). Experimental procedures, kinetic monitoring and data analysis (PDF).

## Conflict of interest

The authors declare no conflict of interest.

## Supporting information

As a service to our authors and readers, this journal provides supporting information supplied by the authors. Such materials are peer reviewed and may be re‐organized for online delivery, but are not copy‐edited or typeset. Technical support issues arising from supporting information (other than missing files) should be addressed to the authors.

Supplementary

## References

[1a] P. Knochel in Organometallics in Synthesis: Organomagnesium and Organozinc Chemistry, Wiley-VCH, Weinheim, 2013;

[1b] P. Knochel , A. Kmsovskiy , L. Sapountzis in Handbook of Functionalized Organometallics: Applications in Synthesis, Wiley-VCH, Weinheim, 2008;

[1c] P. Knochel , W. Dohle , N. Gommermann , F. F. Kneisel , F. Kopp , T. Korn , I. Sapountzis , V. A. Vu , Angew. Chem. Int. Ed. 2003, 42, 4302–4320; 10.1002/anie.200300579 14502700

[1d] For an account on the use of Grignard reagents in process scale, see: U. Tilstam , H. Weinmann , Org. Proc. Res. Dev. 2002, 6, 906–910.

[2] For an NMR solution study on Grignard and turbo-Grignard reagents, see:

[2a] C. Schnegelsberg , S. Bachmann , M. Kolter , T. Auth , M. John , D. Stalke , K. Koszinowski , Chem. Eur. J. 2016, 22, 7752–7762; 27150118 10.1002/chem.201600699

[2b] For an X-ray and CSI-MS study on Grignard reagents, see: S. Sakamoto , T. Imamoto , K. Yamaguchi , Org. Lett. 2001, 3, 1793–1795. 11405713 10.1021/ol010048x

[3] R. M. Peltzer , J. Gauss , O. Eisenstein , M. Cascella , J. Am. Chem. Soc. 2020, 142, 2984–2994. 31951398 10.1021/jacs.9b11829

[4a] D. Seyferth , Organometallics 2009, 28, 1598–1605;

[4b] For a recent computational study, see: R. M. Peltzer , O. Eisenstein , A. Nova , M. Cascella , J. Phys. Chem. B. 2017, 121, 4226–4237. 28358509 10.1021/acs.jpcb.7b02716

[5] For a comprehensive structural review, see: A. Harrison-Marchand , F. Mongin , Chem. Rev. 2013, 113, 7470–7562. 23952819 10.1021/cr300295w

[6a] A. Krasovskiy , P. Knochel , Angew. Chem. Int. Ed. 2004, 43, 3333–3336; 10.1002/anie.200454084 15213967

[6b] P. Knochel , M. N. Barl , V. Werner , C. Sämann , Heterocycles 2014, 88, 827–844.

[7a] C. R. Hauser , H. G. Walker , J. Am. Chem. Soc. 1947, 69, 295–297;

[7b] Eaton introduced DIPA and TMP as hindered magnesium bases: P. E. Eaton , C. H. Lee , Y. Xiong , J. Am. Chem. Soc. 1989, 111, 8016–8018.

[8a] R. Neufeld , T. L. Teuteberg , R. Herbst-Irmer , R. A. Mata , D. Stalke , J. Am. Chem. Soc. 2016, 138, 4796–4806; 27011251 10.1021/jacs.6b00345

[8b] R. Neufeld , D. Stalke , Chem. Eur. J. 2016, 22, 12624–12628; 27224841 10.1002/chem.201601494

[8c] D. R. Armstrong , P. García-Álvarez , A. R. Kennedy , R. E. Mulvey , J. A. Parkinson , Angew. Chem. Int. Ed. 2010, 49, 3185–3188; 10.1002/anie.201000539 20352641

[9] For the solid-state structure of TMPMgCl⋅LiCl, see:

[9a] P. García-Álvarez , D. V. Graham , E. Hevia , A. R. Kennedy , J. Klett , R. E. Mulvey , C. T. O'Hara , S. Weatherstone , Angew. Chem. Int. Ed. 2008, 47, 8079–8081; 10.1002/anie.200802618 18677732

[10a] L. Melzig , C. B. Rauhut , P. Knochel , Chem. Commun. 2009, 3536–3538; 10.1039/b907330b 19521599

[10b] C. J. Rohbogner , S. H. Wunderlich , G. C. Clososki , P. Knochel , Eur. J. Org. Chem. 2009, 1781–1795;

[10c] G. C. Clososki , C. J. Rohbogner , P. Knochel , Angew. Chem. Int. Ed. 2007, 46, 7681–7684; 10.1002/anie.200701487 17665388

[10d] A. Krasovskiy , V. Krasovskaya , P. Knochel , Angew. Chem. Int. Ed. 2006, 45, 2958–2961; 10.1002/anie.200504024 16568481

[11] Hauser-bases like DIPAMgCl are mostly dimeric species that display bridging amido ligands in the solid-state. However, they have been shown to be likely chloro-bridged in solution, thus illustrating the small energetic difference between these coordination manifolds (see ref. [8c]).

[12] The Knochel–Hauser bases DIPAMgCl.LiCl is dimeric in the solid state with bridging amide ligands, but equilibrates with its monomer in solution (see ref. [8c]).

[13] For Hauser bases with terminal bulky amide ligands, see: [HMDSMgBr]_2_:

[13a] K. C. Yang , C. C. Chang , J. Y. Huang , C. C. Lin , G. H. Lee , Y. Wang , M. Y. Chiang , J. Organomet. Chem. 2002, 648, 176–187;

[13b] [HMDSMgCl]_2_: R. A. Bartlett , M. M. Olmstead , P. P. Power , Inorg. Chem. 1994, 33, 4800–4803;

[13c] [(Bn_2_N)_2_Mg]_2_2THF: W. Clegg , F. J. Craig , K. W. Henderson , A. R. Kennedy , R. E. Mulvey , P. A. O'Neil , D. Reed , Inorg. Chem. 1997, 36, 6238–6246;

[13d] For monomeric (HMDS)_2_Mg.2THF, see: D. C. Bradley , M. B. Hursthouse , A. A. Ibrahim , K. M. A. Malik , M. Motevalli , R. Moseler , H. Powell , J. D. Runnacles , A. C. Sullivan , Polyhedron 1990, 9, 2959–2964.

[14] Organomagnesium amides are structurally characterized bases for challenging metallation reactions:

[14a] M.-X. Zhang , P. E. Eaton , Angew. Chem. Int. Ed. 2002, 41, 2169–2171; 19746633

[14b] B. Conway , E. Hevia , A. R. Kennedy , R. E. Mulvey , S. Weatherstone , Dalton Trans. 2005, 1532–1544; 15824793 10.1039/b501502b

[14c] Z. Rappoport , I. Marek , The Chemistry of Organomagnesium Compounds, Wiley, 2008.

[15] For X-ray structures of organomagnesium amides, see: *i*PrMg(DIPA) and EtMg(TMP):

[15a] G. E. Coates , D. Ridley , J. Chem. Soc. A 1967, 56–59;

[15b] *s*BuMg(HMDS) and *t*BuMg(NR_2_)–NR_2_=DIPA, HMDS, TMP, Cy_2_N, Bn_2_N: see ref. [14b]; *n*BuMg(TMP):

[15c] E. Hevia , A. R. Kennedy , R. E. Mulvey , S. Weatherstone , Angew. Chem. Int. Ed. 2004, 43, 1709–1712; 10.1002/anie.200353286 15038044

[15d] RCCMg(DIPA)–R=Ph, TMS: see ref. [13a]; *t*BuMg(NH*t*Bu):

[15e] M. M. Olmstead , W. J. Grigsby , D. R. Chacon , T. Hascall , P. P. Power , Inorg. Chim. Acta 1996, 251, 273–284;

[15f] MeMg(NHTIPS): M. Westerhausen , T. Bollwein , N. Makropoulos , H. Piotrowski , Inorg. Chem. 2005, 44, 6439–6444; 16124825 10.1021/ic0503277

[15g] For a chelate organomagnesium amide: K. W. Henderson , R. E. Mulvey , A. E. Dorigo , J. Organomet. Chem. 1996, 518, 139–146; For a review, see:

[15h] A. G. Pinkus , Coord. Chem. Rev. 1978, 25, 173–197.

[16a] K. Colas , R. Martin-Montero , A. Mendoza , Angew. Chem. Int. Ed. 2017, 56, 16042–16046; 10.1002/anie.201709715 29053208

[16b] K. Colas , A. Mendoza , Synlett 2018, 29, 1329–1333.

[17a] S. Ruppenthal , R. Brückner , J. Org. Chem. 2015, 80, 897–910; 25553340 10.1021/jo502417j

[17b] R. Li-Yuan Bao , R. Zhao , L. Shi , Chem. Commun. 2015, 51, 6884–6900; 10.1039/c4cc10194d 25714498

[17c] C. B. Rauhut , L. Melzig , P. Knochel , Org. Lett. 2008, 10, 3891–3894; 18680381 10.1021/ol801431z

[17d] L. Shi , Y. Chu , P. Knochel , H. Mayr , Org. Lett. 2012, 14, 2602–2605; 22571332 10.1021/ol300906a

[17e] G. Casoni , M. Kucukdisli , J. M. Fordham , M. Burns , E. L. Myers , V. K. Aggarwal , J. Am. Chem. Soc. 2017, 139, 11877–11886; 28812893 10.1021/jacs.7b05457

[17f] S. Oae , Y. Uchida , Acc. Chem. Res. 1991, 24, 202–208.

[18] K. Colas , A. dos Santos , V. D. Catarina , A. Mendoza , Org. Lett. 2019, 21, 7908–7913. 31513423 10.1021/acs.orglett.9b02899

[19] For seminal work, see:

[19a] J. J. Eisch , J. E. Galle , J. Am. Chem. Soc. 1976, 98, 4646–4648; f

[19b] or applications of β-oxyfunctionalized organolithiums, see: J. Barluenga , M. Yus , J. M. Concellon , P. Bernad , J. Org. Chem. 1981, 46, 2721–2726;

[19c] J. Barluenga , M. Yus , J. M. Concellón , P. Bernad , J. Org. Chem. 1983, 48, 609–611;

[19d] J. Barluenga , M. Yus , J. M. Concellón , P. Bernad , J. Org. Chem. 1983, 48, 3116–3118;

[19e] for a comprehensive review, see: C. Nájera , M. Yus , Curr. Org. Chem. 2003, 7, 867–926.

[20a] J. L. García Ruano , J. Alemán , M. B. Cid , M. Á. Fernández-Ibáñez , M. C. Maestro , M. R. Martín , A. M. Martín-Castro in Organosulfur Chemistry in Asymmetric Synthesis: Asymmetric Transformations Mediated by Sulfinyl Groups, Wiley-VCH, Weinheim, 2009;

[20b] S. K. Bur in Comprehensive Organic Synthesis: Polonovski- and Pummerer-Type Reactions and the Nef Reaction, Elsevier, 2014;

[20c] K. S. Feldman , Tetrahedron 2006, 62, 5003–5034;

[20d] A. P. Pulis , D. J. Procter , Angew. Chem. Int. Ed. 2016, 55, 9842–9860; 10.1002/anie.201601540 27409984

[20e] S. K. Bur , A. Padwa , Chem. Rev. 2004, 104, 2401–2432; 15137795 10.1021/cr020090l

[20f] for selected recent examples of Pummerer-based reactions, see: D. Kaldre , B. Maryasin , D. Kaiser , O. Gajsek , L. González , N. Maulide , Angew. Chem. Int. Ed. 2017, 56, 2212–2215; 10.1002/anie.201610105 28097797

[20g] D. Kaiser , L. F. Veiros , N. Maulide , Chem. Eur. J. 2016, 22, 4727–4732; 26918863 10.1002/chem.201600432

[20h] J. A. Fernández-Salas , A. J. Eberhart , D. J. Procter , J. Am. Chem. Soc. 2016, 138, 790–793; 26745643 10.1021/jacs.5b12579

[20i] B. Peng , X. Huang , L.-G. Xie , N. Maulide , Angew. Chem. Int. Ed. 2014, 53, 8718–8721; 10.1002/anie.201310865 24590501

[20j] A. J. Eberhart , D. J. Procter , Angew. Chem. Int. Ed. 2013, 52, 4008–4011; 10.1002/anie.201300223 23447131

[20k] A. J. Eberhart , J. E. Imbriglio , D. J. Procter , Org. Lett. 2011, 13, 5882–5885; 21999481 10.1021/ol2025197

[20l] H. Yorimitsu , Chem. Rec. 2017, 17, 1156–1167; 28488753 10.1002/tcr.201700017

[20m] L. H. S. Smith , S. C. Coote , H. E. Sneddon , D. J. Procter , Angew. Chem. Int. Ed. 2010, 49, 5832–5844; 10.1002/anie.201000517 20583014

[21a] A. D. Becke , J. Chem. Phys. 1993, 98, 5648–5652;

[21b] C. Lee , W. Yang , R. G. Parr , Phys. Rev. B 1988, 37, 785–789. 10.1103/physrevb.37.785 9944570

[22a] S. Grimme , J. Antony , S. Ehrlich , H. Krieg , J. Chem. Phys. 2010, 132, 154104; 20423165 10.1063/1.3382344

[22b] S. Grimme , S. Ehrlich , L. Goerigk , J. Comput. Chem. 2011, 32, 1456–1465. 21370243 10.1002/jcc.21759

[23] W.-B. Liu , D. P. Schuman , Y.-F. Yang , A. A. Toutov , Y. Liang , H. F. T. Klare , N. Nesnas , M. Oestreich , D. G. Blackmond , S. C. Virgil , S. Banerjee , R. N. Zare , R. H. Grubbs , K. N. Houk , B. M. Stoltz , J. Am. Chem. Soc. 2017, 139, 6867–6879. 28403611 10.1021/jacs.6b13031

[24a] M. Ishimori , T. Hagiwara , T. Tsuruta , Y. Kai , N. Yasuoka , N. Kasai , Bul. Chem. Soc. Jpn. 1976, 49, 1165–1166;

[24b] M. Ullrich , R. J. F. Berger , S. Jana , T. Pape , R. Fröhlich , N. W. Mitzel , Dalton Trans. 2011, 40, 1144–1157; 21165506 10.1039/c0dt01100b

[24c] R. Petrus , P. Sobota , Organometallics 2012, 31, 4755–4762;

[24d] S. V. Athavale , A. Simon , K. N. Houk , S. E. Denmark , Nat. Chem. 2020, 12, 412–423. 32203445 10.1038/s41557-020-0421-8 PMC7117993

[25] Gaussian 09, Revision E.01, M. J. Frisch, G. W. Trucks, H. B. Schlegel, G. E. Scuseria, M. A. Robb, J. R. Cheeseman, G. Scalmani, V. Barone, B. Mennucci, G. A. Petersson, H. Nakatsuji, M. Caricato, X. Li, H. P. Hratchian, A. F. Izmaylov, J. Bloino, G. Zheng, J. L. Sonnenberg, M. Hada, M. Ehara, K. Toyota, R. Fukuda, J. Hasegawa, M. Ishida, T. Nakajima, Y. Honda, O. Kitao, H. Nakai, T. Vreven, J. A. Montgomery, Jr., J. E. Peralta, F. Ogliaro, M. Bearpark, J. J. Heyd, E. Brothers, K. N. Kudin, V. N. Staroverov, T. Keith, R. Kobayashi, J. Normand, K. Raghavachari, A. Rendell, J. C. Burant, S. S. Iyengar, J. Tomasi, M. Cossi, N. Rega, J. M. Millam, M. Klene, J. E. Knox, J. B. Cross, V. Bakken, C. Adamo, J. Jaramillo, R. Gomperts, R. E. Stratmann, O. Yazyev, A. J. Austin, R. Cammi, C. Pomelli, J. W. Ochterski, R. L. Martin, K. Morokuma, V. G. Zakrzewski, G. A. Voth, P. Salvador, J. J. Dannenberg, S. Dapprich, A. D. Daniels, O. Farkas, J. B. Foresman, J. V. Ortiz, J. Cioslowski, D. J. Fox, Gaussian Inc., Wallingford CT, **2013**.

[26] A. V. Marenich , C. J. Cramer , D. G. Truhlar , J. Phys. Chem. B. 2009, 113, 6378–6396. 19366259 10.1021/jp810292n

