# Supplementary material for: Computational and Experimental Study of Turbo‐Organomagnesium Amide Reagents: Cubane Aggregates as Reactive Intermediates in Pummerer Coupling
Source: Chemistry. 2021 Jan 12;27(8):2767–73. doi: 10.1002/chem.202004164 (PMC7898302; doi:10.1002/chem.202004164)
Supplement: Supplementary file 1 — Supplementary [file CHEM-27-2767-s001.pdf]

Chemistry–A European Journal

Supporting Information

Computational and Experimental Study of Turbo-Organomagnesium Amide Reagents: Cubane Aggregates as Reactive Intermediates in Pummerer Coupling

Ferran Planas,[a] Stefanie V. Kohlhepp,[a] Genping Huang,[b] Abraham Mendoza,*[a] and Fahmi Himo*[a]

Part 1: Computational Details

Table of Contents

1. Calculated aggregates formed from the Grignard reagent and the magnesium amide S1-3
2. Calculated complexes formed after inclusion of the sulfoxide S1-4
3. Energies for the formation of complexes **B-D** from complex **A** S1-5
4. Optimized geometries complexes **A-D** S1-6
5. Geometries for the reaction mechanism of complex **A** S1-7
6. Geometries for the reaction mechanism of complex **B** S1-8
7. Energy for the elongation of the S-O bond to form a sulfonium intermediate S1-9
8. Alternative deprotonation pathways in complex **C**. S1-10
9. Free energy profile and geometries for the reaction mechanism of complex **D** S1-11
10. Calculated absolute energies and energy corrections S1-122
11. Cartesian coordinates S1-133

1. Calculated aggregates formed from the Grignard reagent and the magnesium amide

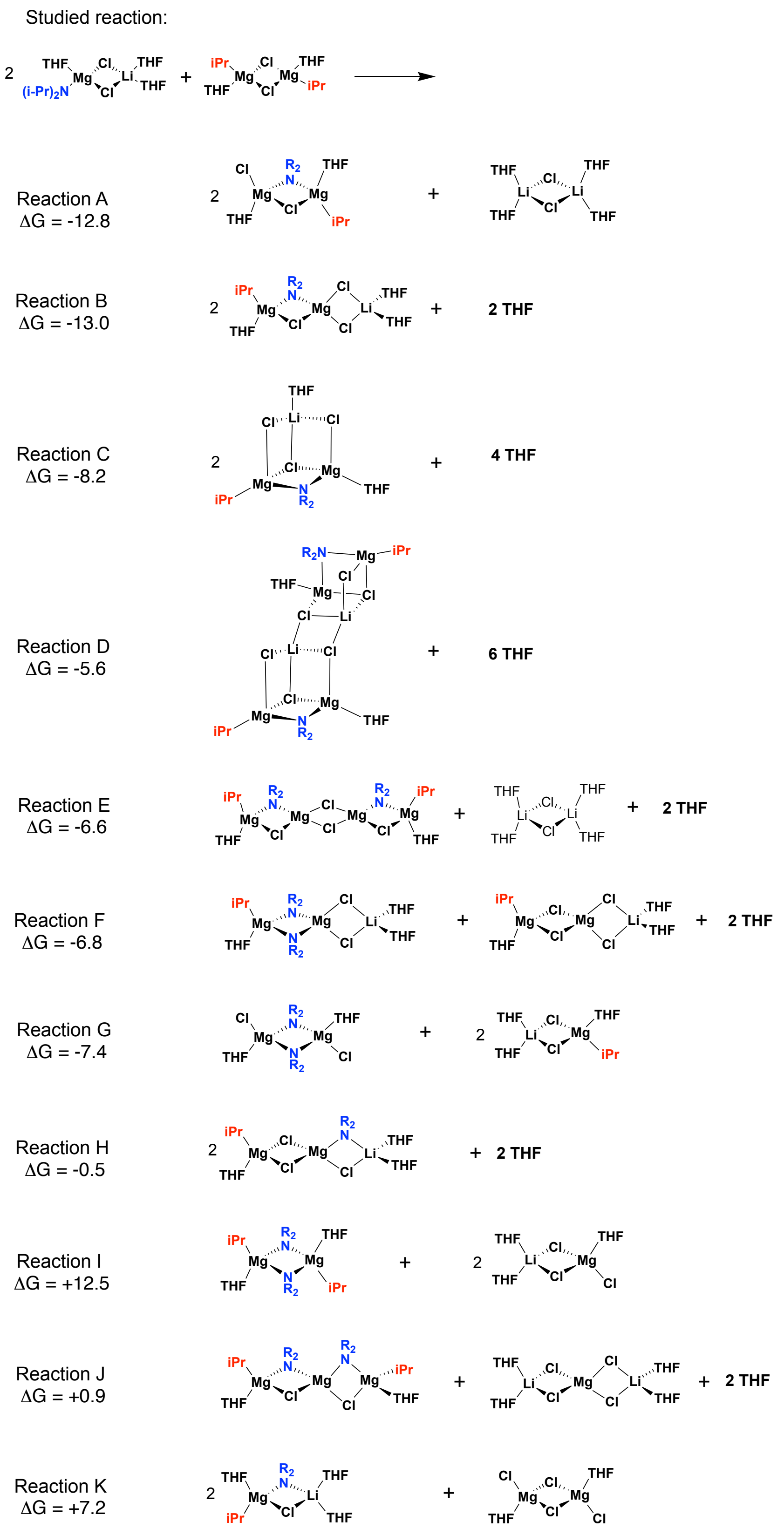

Figure S1. *Reaction free energies (in kcal/mol) of calculated aggregates formed from the Grignard reagent and the magnesium amide.*

2. Calculated complexes formed after inclusion of the sulfoxide

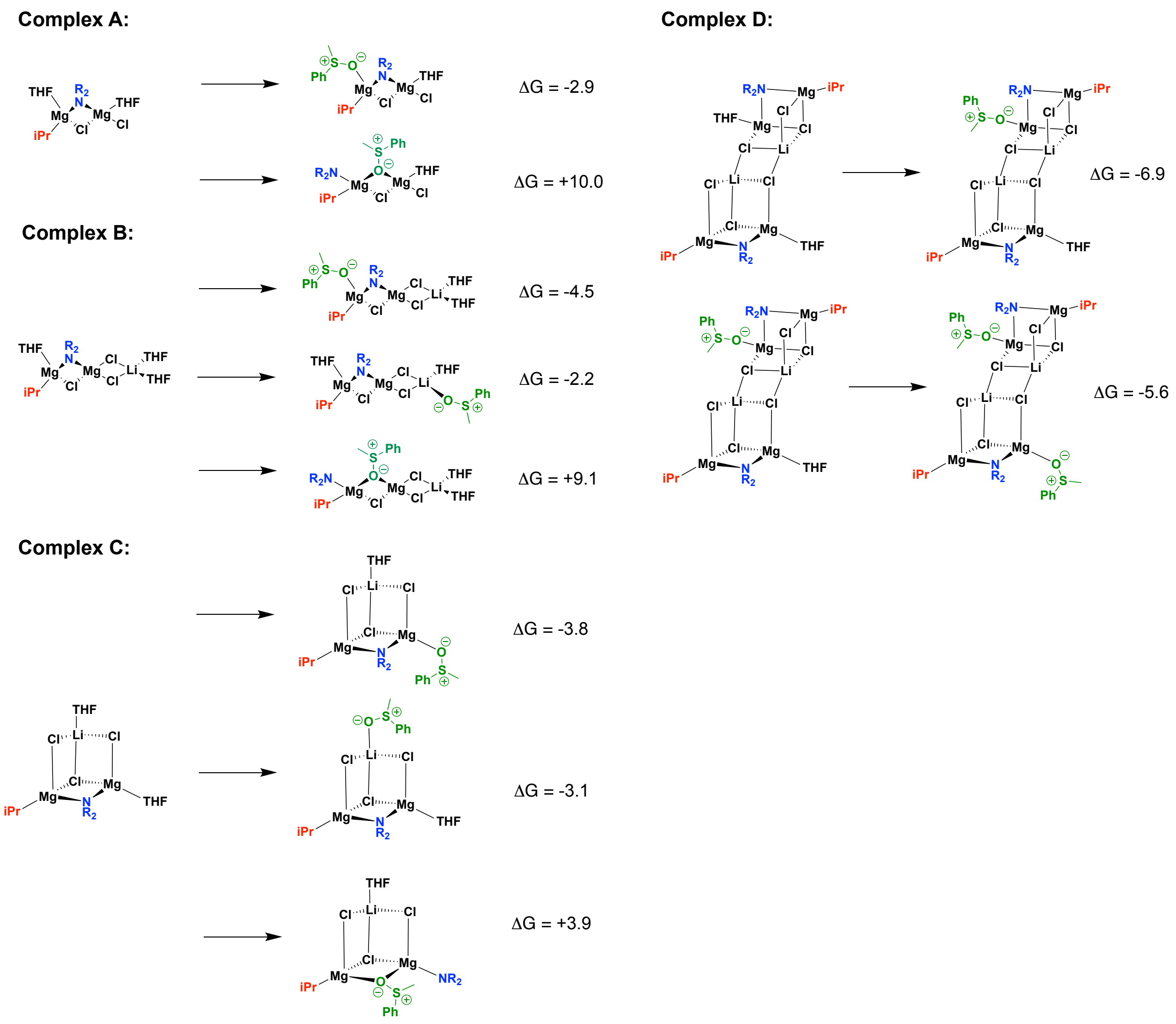

Figure S2. *Complexes including a sulfoxide molecule. The reaction free energies (in kcal/mol) correspond to the exchange reaction of one THF molecule for the sulfoxide.*

3. Energies for the formation of complexes B-D from complex A

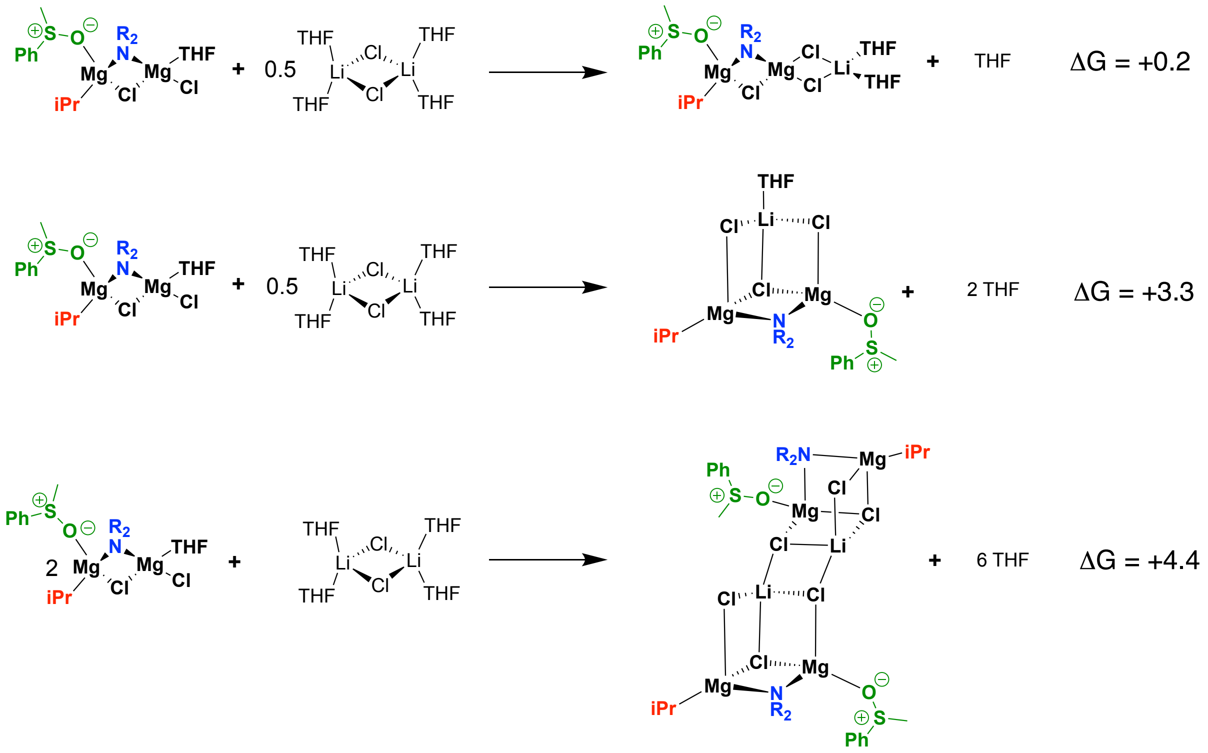

Figure S3. *Calculated free energies (in kcal/mol) for the formation of complexes* ***B****,* ***C*** *and* ***D*** *from complex* ***A****.*

4. Optimized geometries complexes A-D

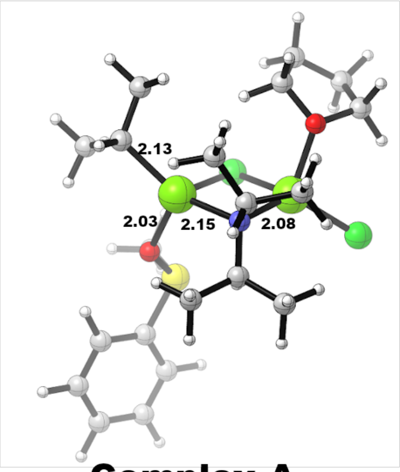

Complex A

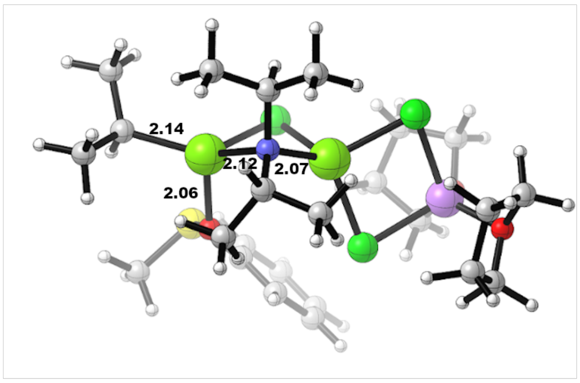

Complex B

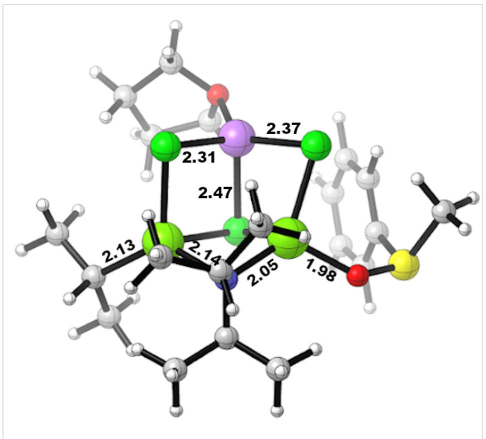

Complex C

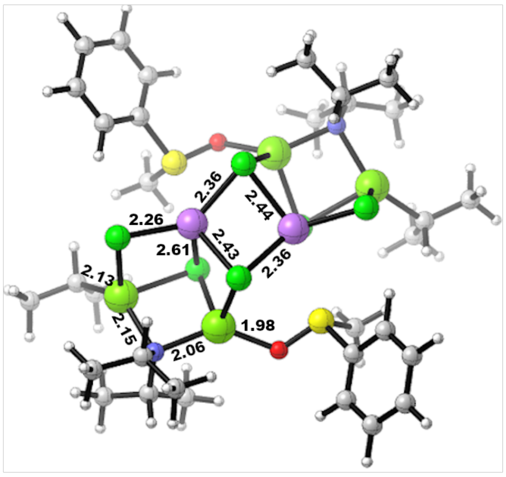

Complex D

Figure S4. *Optimized geometries of the four complexes used in the mechanistic investigation. Relevant bond distances are given in Å.*

5. Geometries for the reaction mechanism of complex A

.

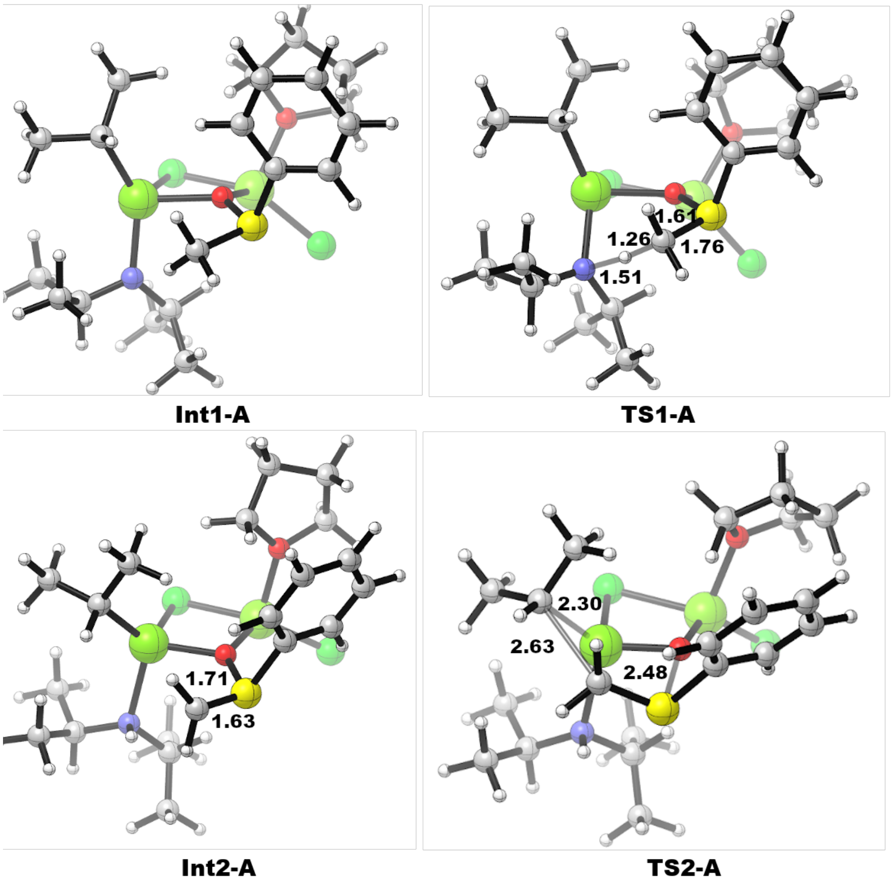

Figure S5. *Optimized geometries of the relevant stationary points of the calculated reaction mechanism for complex **A**.*

6. Geometries for the reaction mechanism of complex B

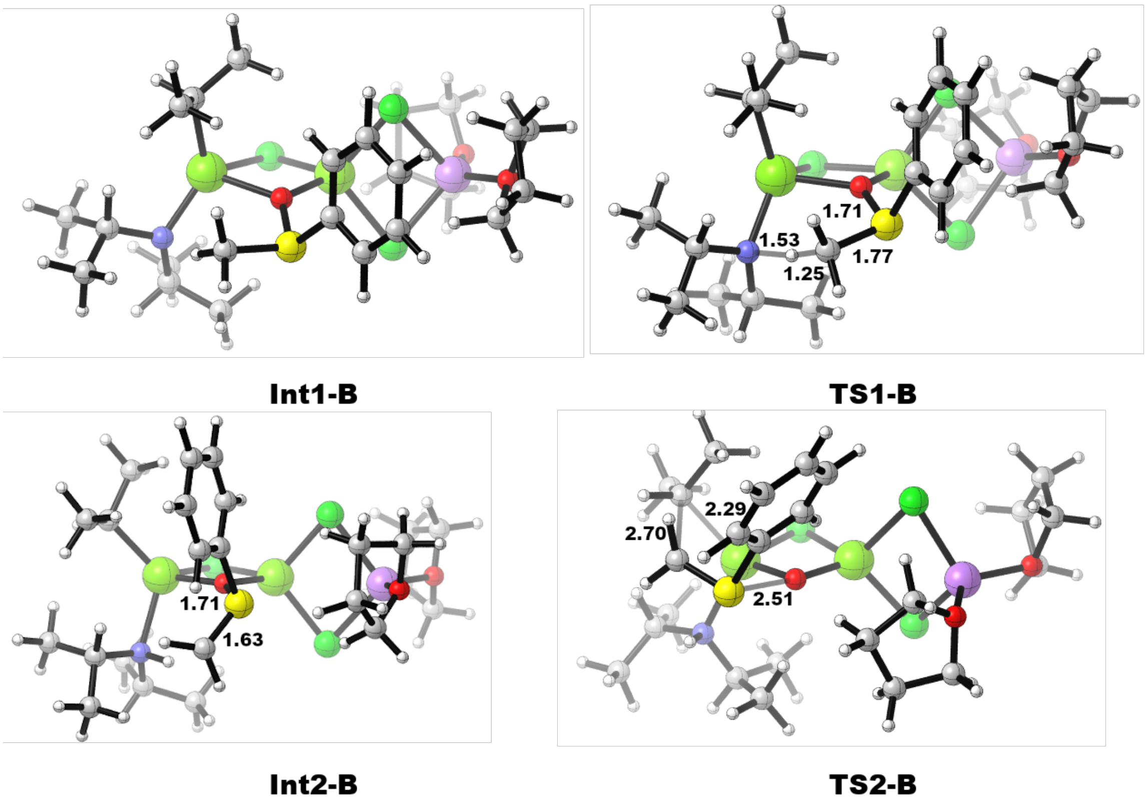

Figure S6. *Optimized geometries of the relevant stationary points of the calculated reaction mechanism for complex **B**.*

7. Energy for the elongation of the S-O bond to form a sulfonium intermediate

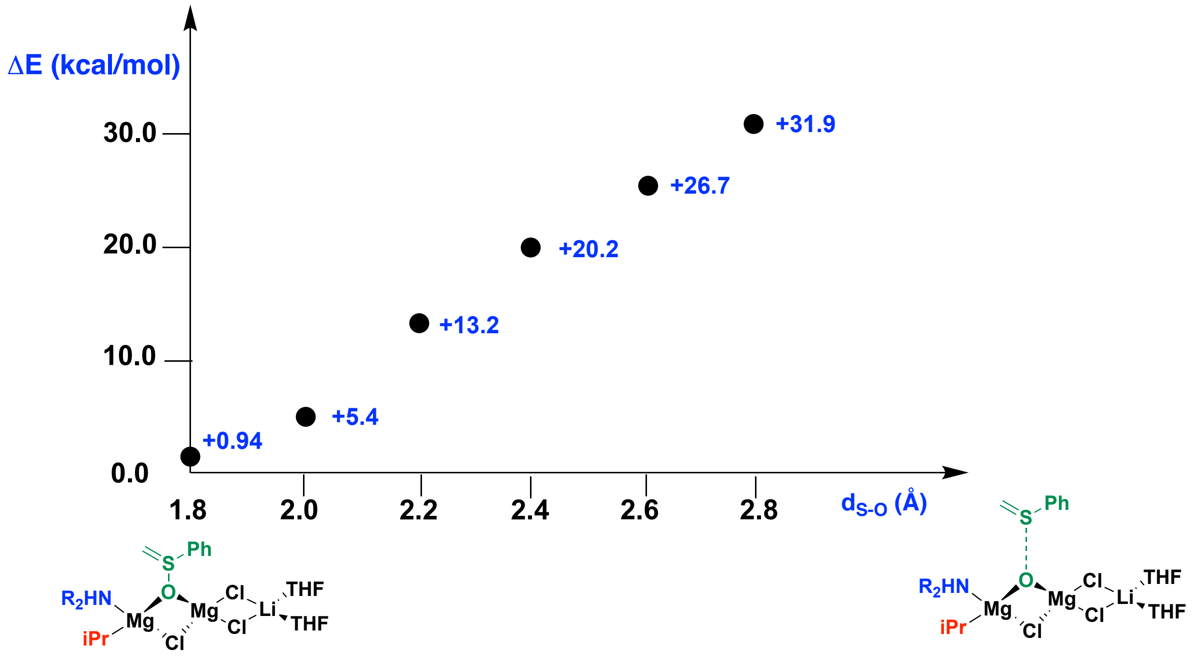

*Figure S7. Linear transit energies for the elongation of the S-O bond at **Int1-B**.*

8. Alternative deprotonation pathways in complex C.

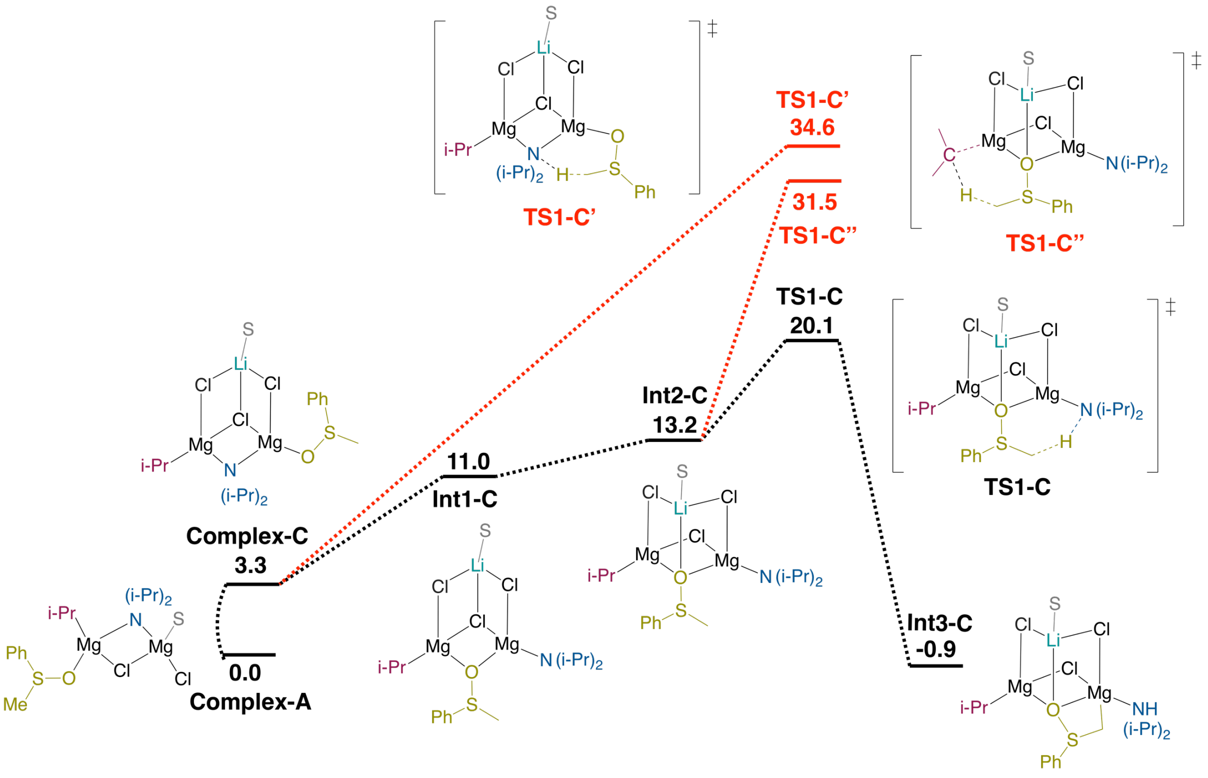

Figure S8. *Comparison of alternative deprotonations of the sulfoxide.* ***TS1-C***: *Deprotonation of the bridging sulfoxide by the amide.* ***TS1-C'***: *Deprotonation of the terminal sulfoxide by the amide.* ***TS1-C''***: *Deprotonation of the bridging sulfoxide by the isopropyl moiety. All energies are in kcal/mol*

9. Free energy profile and geometries for the reaction mechanism of complex D

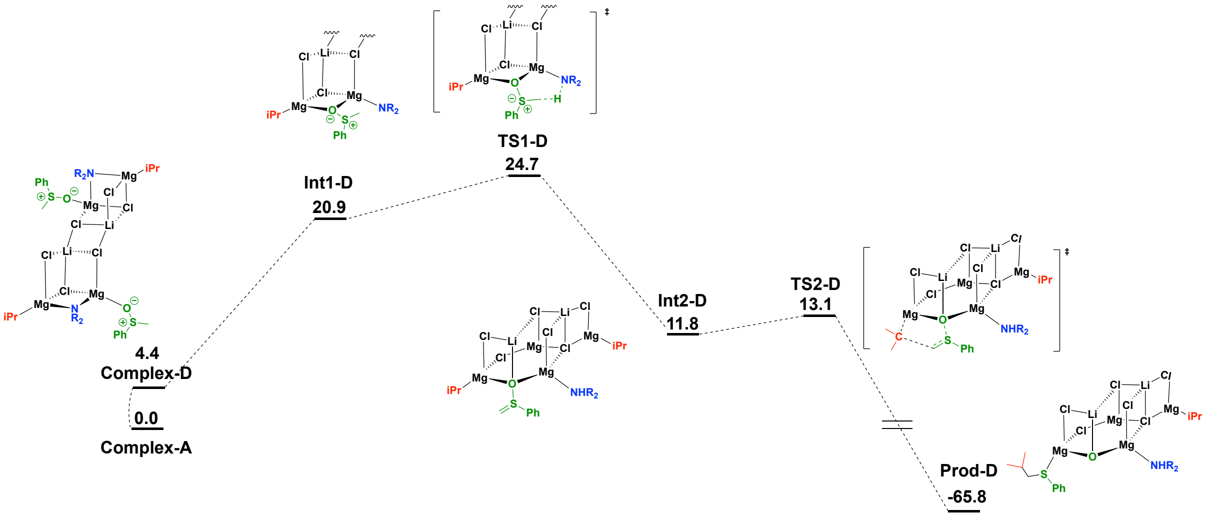

Figure S9. *Free energy profile (kcal/mol) of the calculated reaction mechanism of complex* ***D****.*

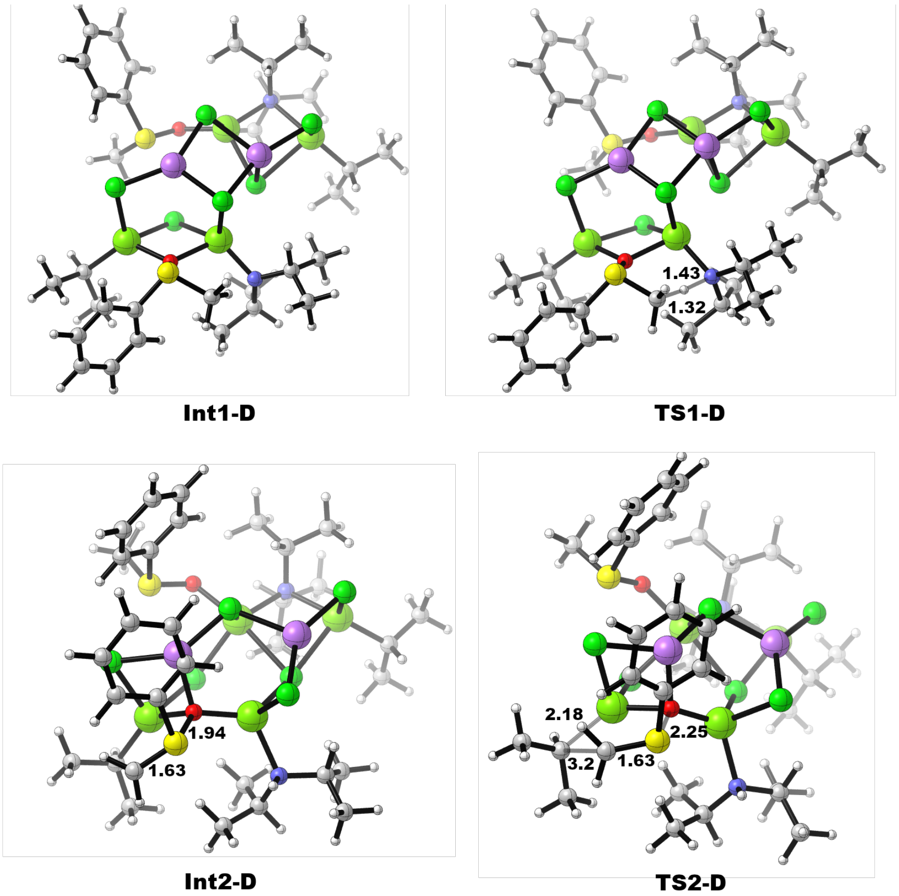

Figure S10. *Optimized geometries of the relevant stationary points of the calculated reaction mechanism for complex* ***D****.*

10. Calculated absolute energies and energy corrections

Stationary point	E_{el} (a.u.)	E_{solv} (a.u.)	E_{bb} (a.u.)	G_{corr} (a.u.)	G_{tot} (a.u.)	ΔG (kcal/mol)
**$[LiCl]_2$**	-1865.74747	-1865.77283	-1866.06098	0.41696	-1865.66635	-
**THF**	-232.47579	-232.48167	-232.54340	0.08866	-232.45520	-
**A**	-2708.80512	-2708.83877	-2709.20048	0.47260	-2708.75852	**0.0**
**Int1-A**	-2708.78422	-2708.81972	-2709.17776	0.47225	-2708.73799	**+12.9**
**TS1-A**	-2708.78258	-2708.81501	-2709.17498	0.47272	-2708.73167	**+16.8**
**Int2-A**	-2708.80892	-2708.84036	-2709.20459	0.47604	-2708.75697	**+1.0**
**TS2-A**	-2708.76604	-2708.80180	-2709.15889	0.47623	-2708.71539	**+27.1**
**Prod-A**	-2708.89711	-2708.93495	-2709.28414	0.48048	-2708.83847	**-50.2**
**B**	-3409.20197	-3409.23365	-3409.69149	0.58394	-3409.13621	**+0.2**
**Int1-B**	-3409.17521	-3409.21140	-3409.66346	0.58241	-3409.11420	**+14.0**
**TS1-B**	-3409.17161	-3409.20569	-3409.65903	0.58063	-3409.10945	**+17.0**
**Int2-B**	-3409.20052	-3409.23296	-3409.69054	0.58498	-3409.13498	**+1.0**
**TS2-B**	-3409.16004	-3409.19531	-3409.64641	0.58695	-3409.09171	**+28.1**
**Prod-B**	-3409.28424	-3409.32405	-3409.76608	0.58986	-3409.21300	**-48.0**
**C**	-3176.69460	-3176.72792	-3177.12165	0.47581	-3176.67613	**+3.3**
**Int1-C**	-3176.67704	-3176.71015	-3177.10360	0.46992	-3176.66375	**+11.0**
**Int2-C**	-3176.67525	-3176.71213	-3177.10076	0.47432	-3176.66030	**+13.2**
**TS1-C**	-3176.66405	-3176.69917	-3177.08787	0.47074	-3176.64923	**+20.1**
**Int3-C**	-3176.69635	-3176.73350	-3177.12256	0.47399	-3176.68270	**-0.9**
**Int4-C**	-3176.69077	-3176.72715	-3177.11653	0.47583	-3176.6741	**+4.6**
**TS2-C**	-3176.67888	-3176.71555	-3177.10240	0.47744	-3176.65861	**+14.3**
**Prod-C**	-3176.81202	-3176.84630	-3177.23077	0.48028	-3176.78174	**-63.0**
**D**	-5888.42281	-5888.47070	-5889.15235	0.75194	-5888.44527	**+4.4**
**Int1-D**	-5888.39365	-5888.44245	-5889.12327	0.74999	-5888.41905	**+20.9**
**TS1-D**	-5888.38865	-5888.43479	-5889.11724	0.74743	-5888.41292	**+24.9**
**Int2-D**	-5888.41456	-5888.46560	-5889.14049	0.75589	-5888.43262	**+11.8**
**TS2-D**	-5888.41214	-5888.46369	-5889.13818	0.75533	-5888.43137	**+13.1**
**Prod-D**	-5888.54212	-5888.59371	-5889.26565	0.75714	-5888.55708	**-65.8**

E_{el}: Energy of the optimized geometry at B3LYP-D3(BJ)/6-31G(d,p) level.
E_{solv}: Single-point solvation energy calculated at the B3LYP-D3(BJ)/6-31G(d,p) level using the SMD method with THF as solvent.
E_{bb}: Single-point energy at the B3LYP-D3(BJ)/6-311+G(2d,2p) level.
G_{corr}: Thermal correction to Gibbs free energy calculated at B3LYP-D3(BJ)/6-31G(d,p) level.
G_{tot}: Total Gibbs free energy calculated as $G_{tot} = E_{bb} + (E_{solv} – E_{el}) + G_{corr} + SSc$
SSc: Standard state correction of +1.9 kcal/mol for all solutes, and +3.4 kcal/mol for the solvent.
ΔG: Relative Gibbs free energy with respect to complex **A**.

11. Cartesian coordinates

Complex C

Mg	2.84192400	0.38762300	1.15272100
Cl	4.78431200	−1.01581600	1.41642000
C	1.46689600	0.62586100	2.76078500
H	0.56816900	0.01340800	2.57311300
N	2.56028400	0.62288100	−0.96346400
Li	6.14710100	0.82207400	1.08789900
C	5.83057400	1.86143100	4.44278100
H	5.91654900	2.39060500	5.39504200
H	4.82117100	2.02011200	4.05767400
C	6.13131000	0.34320400	4.56918300
H	5.26090300	−0.24256100	4.27013700
H	6.41010600	0.05451200	5.58550200
C	6.86366700	2.36163700	3.42633900
H	7.77555600	2.72989500	3.91663100
H	6.48315000	3.11892400	2.74267300
C	7.28720200	0.10782900	3.58649800
H	8.26613900	0.15307800	4.08081300
H	7.20344000	−0.82052000	3.02115300
O	7.20728600	1.20102200	2.63906000
C	2.35505500	−0.64832500	−1.71084800
C	1.42003600	1.58269600	−1.10286000
H	1.58735300	−0.49101600	−2.47958100
H	1.66614600	2.42574500	−0.43949600
C	3.64132800	−1.05963900	−2.43336300
C	1.87666200	−1.80115200	−0.81187200
C	1.27088700	2.15881600	−2.52226400
C	0.06751400	1.02578600	−0.62898300
H	2.18431900	2.66016200	−2.85169100
H	1.03048400	1.37382100	−3.24687100
H	0.45532000	2.88948100	−2.55341300
H	−0.28002000	0.21810800	−1.28111400
H	0.12522900	0.64478100	0.39315100
H	−0.69168200	1.81432600	−0.65091900
H	1.64401600	−2.69241500	−1.40555900
H	2.65963100	−2.08205600	−0.09878000
H	0.97670300	−1.52763600	−0.25533300
H	3.94679200	−0.29629700	−3.16257500
H	4.45855700	−1.21003300	−1.72008400
H	3.51029500	−1.99621900	−2.98523500
Mg	4.34587600	1.60147500	−1.26581200
Cl	4.36752700	2.52780200	0.98391000
Cl	6.59382200	0.80566100	−1.24649900
C	1.00294000	2.08383600	2.90542400
H	1.85329400	2.75973400	3.07253900
H	0.48585400	2.44793400	2.00806600
H	0.31257600	2.23516800	3.75478200
C	2.06965100	0.13678300	4.08702000
H	1.38267700	0.25706500	4.94398800
H	2.35684200	−0.92114000	4.04743900
H	2.97788500	0.69942800	4.34719700
S	5.43990600	4.28094600	−2.77758900
O	4.44555600	3.10320500	−2.56423900
C	6.16230300	4.74767600	−1.19975900
C	5.50840300	5.79658200	−0.54607800
C	7.25432400	4.09999000	−0.61619400
C	5.95397800	6.20308800	0.70915500
H	4.65639300	6.28276000	−1.01105800
C	7.69918800	4.52585200	0.63253300
H	7.73207600	3.25894300	−1.09866200
C	7.05082900	5.57056500	1.29445500
H	5.44646800	7.01019600	1.22625100
H	8.54411800	4.02600000	1.09429200
H	7.39968100	5.88915100	2.27163300
C	6.86233800	3.53782200	−3.62554600
H	7.16470000	2.62992200	−3.10068400
H	7.66615900	4.27486100	−3.67220200
H	6.51536500	3.29042800	−4.62969000

Int1-C

Mg	2.84192400	0.38762300	1.15272100
Cl	4.78431200	−1.01581600	1.41642000
C	1.46689600	0.62586100	2.76078500
H	0.56816900	0.01340800	2.57311300
N	2.56028400	0.62288100	−0.96346400
Li	6.14710100	0.82207400	1.08789900
C	5.83057400	1.86143100	4.44278100
H	5.91654900	2.39060500	5.39504200
H	4.82117100	2.02011200	4.05767400
C	6.13131000	0.34320400	4.56918300
H	5.26090300	−0.24256100	4.27013700
H	6.41010600	0.05451200	5.58550200
C	6.86366700	2.36163700	3.42633900
H	7.77555600	2.72989500	3.91663100
H	6.48315000	3.11892400	2.74267300
C	7.28720200	0.10782900	3.58649800
H	8.26613900	0.15307800	4.08081300
H	7.20344000	−0.82052000	3.02115300
O	7.20728600	1.20102200	2.63906000
C	2.35505500	−0.64832500	−1.71084800
C	1.42003600	1.58269600	−1.10286000
H	1.58735300	−0.49101600	−2.47958100
H	1.66614600	2.42574500	−0.43949600
C	3.64132800	−1.05963900	−2.43336300
C	1.87666200	−1.80115200	−0.81187200
C	1.27088700	2.15881600	−2.52226400
C	0.06751400	1.02578600	−0.62898300
H	2.18431900	2.66016200	−2.85169100
H	1.03048400	1.37382100	−3.24687100
H	0.45532000	2.88948100	−2.55341300
H	−0.28002000	0.21810800	−1.28111400
H	0.12522900	0.64478100	0.39315100
H	−0.69168200	1.81432600	−0.65091900
H	1.64401600	−2.69241500	−1.40555900
H	2.65963100	−2.08205600	−0.09878000
H	0.97670300	−1.52763600	−0.25533300
H	3.94679200	−0.29629700	−3.16257500
H	4.45855700	−1.21003300	−1.72008400
H	3.51029500	−1.99621900	−2.98523500
Mg	4.34587600	1.60147500	−1.26581200
Cl	4.36752700	2.52780200	0.98391000
Cl	6.59382200	0.80566100	−1.24649900
C	1.00294000	2.08383600	2.90542400
H	1.85329400	2.75973400	3.07253900
H	0.48585400	2.44793400	2.00806600
H	0.31257600	2.23516800	3.75478200
C	2.06965100	0.13678300	4.08702000
H	1.38267700	0.25706500	4.94398800
H	2.35684200	−0.92114000	4.04743900
H	2.97788500	0.69942800	4.34719700
S	5.43990600	4.28094600	−2.77758900
O	4.44555600	3.10320500	−2.56423900
C	6.16230300	4.74767600	−1.19975900
C	5.50840300	5.79658200	−0.54607800
C	7.25432400	4.09999000	−0.61619400
C	5.95397800	6.20308800	0.70915500
H	4.65639300	6.28276000	−1.01105800
C	7.69918800	4.52585200	0.63253300
H	7.73207600	3.25894300	−1.09866200
C	7.05082900	5.57056500	1.29445500
H	5.44646800	7.01019600	1.22625100
H	8.54411800	4.02600000	1.09429200
H	7.39968100	5.88915100	2.27163300
C	6.86233800	3.53782200	−3.62554600
H	7.16470000	2.62992200	−3.10068400
H	7.66615900	4.27486100	−3.67220200
H	6.51536500	3.29042800	−4.62969000

Int2-C

Mg	2.38171000	0.43389700	1.52371200
Cl	3.48422600	−1.66722900	1.77591900
C	0.54345400	1.30501200	2.11011200
H	−0.29066200	0.62825000	1.86537200
N	4.04155900	3.09288500	−2.02390100
Li	5.46786300	−0.54157000	1.31805500
C	7.43098800	1.32547700	3.81670900
H	8.30824100	1.59316700	4.41078800
H	6.99768500	2.24399700	3.41756900
C	6.37599600	0.54515900	4.64600300
H	5.39149400	1.00098100	4.52294200
H	6.61060600	0.51979500	5.71282300
C	7.81451300	0.35870700	2.67998900
H	8.77464400	−0.13540500	2.87241500
H	7.83929000	0.81235400	1.68793800
C	6.38577000	−0.85738500	4.03034700
H	7.12463400	−1.51103300	4.51221100
H	5.41519600	−1.35534400	4.01498200

O	6.77976900	−0.64909700	2.65841200
C	5.04335900	3.86376800	−2.76133000
C	2.71868100	3.71288100	−1.90078300
H	4.69840800	4.89858800	−2.91065900
H	2.06950500	2.95363100	−1.43679100
C	6.35836100	3.94988500	−1.97049500
C	5.31044900	3.26040100	−4.15172400
C	2.69802700	4.92882700	−0.95328900
C	2.06522400	4.07959300	−3.24675700
H	3.06723000	4.64176600	0.03664400
H	3.33953900	5.73278000	−1.33098000
H	1.68684700	5.33589200	−0.83547400
H	2.62945900	4.86187800	−3.76468200
H	2.01796900	3.21212800	−3.91290500
H	1.04639900	4.45474900	−3.09842300
H	6.05126100	3.83822800	−4.71833800
H	5.68813400	2.23654300	−4.04308600
H	4.38881900	3.21783600	−4.73886800
H	6.19308300	4.44406600	−1.00674100
H	6.76072200	2.94397200	−1.78440600
H	7.13277500	4.50515100	−2.51195000
Mg	4.53366900	1.83934400	−0.60232400
Cl	4.39029300	1.90346000	1.82341700
Cl	6.14913300	0.12356100	−0.80059900
C	0.33480400	2.60939800	1.32199600
H	1.15039500	3.32351100	1.49898500
H	0.29889800	2.45087700	0.23511900
H	−0.59858500	3.13089300	1.59710800
C	0.47912900	1.59266200	3.61835600
H	−0.45112600	2.10876300	3.91423700
H	0.53989300	0.67523700	4.21725500
H	1.30482700	2.24211400	3.94091000
S	2.74274900	−0.55662300	−1.55994800
O	2.85474000	0.61680500	−0.49508900
C	1.14822600	−1.27634000	−1.19648200
C	1.08232200	−2.66522900	−1.08403300
C	0.04273300	−0.45885000	−0.94617800
C	−0.13665300	−3.25142500	−0.74446400
H	1.96985900	−3.27011800	−1.23527900
C	−1.16274900	−1.05799200	−0.59485100
H	0.13172700	0.62074100	−0.98812600
C	−1.25240600	−2.44992500	−0.50077200
H	−0.20714900	−4.32971000	−0.65106700
H	−2.02613900	−0.43749900	−0.38101600
H	−2.19480900	−2.90979800	−0.22156300
C	2.41432000	0.39045900	−3.06968600
H	1.41303900	0.81771500	−3.02262000
H	2.51973900	−0.28330900	−3.92206100
H	3.16675700	1.19804300	−3.07811200

TS1-C

Mg	3.37538000	−0.54798500	1.21012000
Cl	4.92860900	−2.36507600	1.27143300
C	1.45469300	−0.35035900	2.08459500
H	0.79980800	−1.18564700	1.79439100
N	3.94447000	2.49341700	−2.38076900
Li	5.46131400	−1.54865000	−0.81033500
C	7.27773400	−4.32170400	−1.86629100
H	7.92017200	−5.01230600	−2.41761100
H	7.87631100	−3.86606400	−1.07323700
C	6.02108700	−5.01288600	−1.26733100
H	6.05343200	−4.99498500	−0.17642900
H	5.92470400	−6.05229100	−1.59011000
C	6.69747400	−3.23487200	−2.77719800
H	6.50908200	−3.61100400	−3.79136700
H	7.28181900	−2.31632500	−2.83192500
C	4.84546400	−4.16232800	−1.77887500
H	4.36582200	−4.60837100	−2.65934900
H	4.09434900	−3.95011100	−1.01797600
O	5.43009300	−2.89984600	−2.17002800
C	4.80282100	3.36514700	−3.21482300
C	2.69234400	3.10764200	−1.88523200
H	4.21310200	4.21388400	−3.58376400
H	2.17655300	2.30249300	−1.34154600
C	5.98721300	3.93838900	−2.41875500
C	5.31240400	2.58962300	−4.43594500
C	2.90315300	4.25022500	−0.88278100
C	1.75579600	3.56197600	−3.01949100
H	3.50959600	3.92327000	−0.03352900
H	3.39847400	5.10404100	−1.35622200
H	1.94133600	4.59955100	−0.49243400
H	2.11812100	4.47456400	−3.50180500
H	1.66671100	2.79641400	−3.79688700
H	0.75665700	3.77890600	−2.62725200
H	5.97791700	3.21149700	−5.04293200
H	5.87598300	1.70473600	−4.12084000
H	4.48159400	2.26052300	−5.06758600
H	5.64680600	4.47322900	−1.52819300
H	6.66643800	3.13502400	−2.10441200
H	6.57706200	4.63324300	−3.02691800
Mg	4.88568400	1.34836100	−0.99467000
Cl	4.68454300	1.55341200	1.34052000
Cl	6.83424500	0.18222400	−1.58292100
C	0.79916500	0.95166300	1.59845200
H	1.41553300	1.82510900	1.85105100
H	0.65574100	0.96837200	0.51224400
H	−0.19213800	1.12870000	2.05285100
C	1.54980700	−0.34927500	3.61887900
H	0.57235800	−0.17950600	4.10434900
H	1.94247700	−1.29590200	4.01059500
H	2.21291100	0.44797000	3.98289300
S	2.86552400	−1.02767900	−2.13804100
O	3.70959700	−0.36359100	−0.90016500
C	1.14181700	−0.75150400	−1.74273700
C	0.55549600	−1.72944600	−0.93433300
C	0.40468000	0.34327900	−2.20148800
C	−0.78106100	−1.59760300	−0.56451400
H	1.14067800	−2.57439700	−0.58595200
C	−0.93447700	0.45523400	−1.83832600
H	0.86336500	1.09599400	−2.82550800
C	−1.52413900	−0.50750700	−1.01615300
H	−1.23583800	−2.34282100	0.07847800
H	−1.51351500	1.30327400	−2.18846800
H	−2.56462800	−0.40583600	−0.72583800
C	3.19154900	0.09243700	−3.47360700
H	3.52304300	1.31675700	−3.01787600
H	2.32534700	0.09956300	−4.13698400
H	4.05268000	−0.32889600	−3.99861900

Int3-C

Mg	3.17851600	−0.55002800	0.79655800
Cl	5.24135600	−1.76175900	0.76594800
C	1.55821700	−0.62286900	2.16999000
H	1.11266000	−1.63099500	2.16669000
N	4.25178800	3.19526200	−3.15401100
Li	4.77693100	−1.74253100	−1.53084500
C	7.22323700	−4.09708000	−1.61089400
H	8.11992000	−4.64994900	−1.90162500
H	7.53022300	−3.20194200	−1.06349000
C	6.24761800	−4.93676500	−0.75148800
H	6.33616800	−4.67964200	0.30491000
H	6.43317000	−6.00834800	−0.86359900
C	6.37008300	−3.69538800	−2.81191500
H	6.32133700	−4.49242000	−3.56646300
H	6.66072200	−2.75956600	−3.29179500
C	4.85047900	−4.56724100	−1.29650600
H	4.37538900	−5.40834000	−1.81527100
H	4.17852000	−4.18362600	−0.52757100
O	5.05538300	−3.49772600	−2.25045600
C	5.62264500	3.82343600	−3.10587900
C	3.10381200	4.15137300	−2.99078700
H	5.49754700	4.90846900	−3.19069300
H	2.21214000	3.51392600	−2.97132800
C	6.32436800	3.51798600	−1.78353100
C	6.44270700	3.34457400	−4.30351400
C	3.17248400	4.90086600	−1.66199300
C	2.97342900	5.10198500	−4.18518000
H	3.24511600	4.21349000	−0.81714900
H	4.02436000	5.58635100	−1.62810600
H	2.26526100	5.49895800	−1.53917900
H	3.83047000	5.77797400	−4.26252900
H	2.88941300	4.54785400	−5.12659900
H	2.07603300	5.71852800	−4.07928600
H	7.44027900	3.79165200	−4.28065900
H	6.55655600	2.25713500	−4.28114500
H	5.96822700	3.63175000	−5.24885700
H	5.72227600	3.81509400	−0.92347900
H	6.54315500	2.44924100	−1.69592900
H	7.27562500	4.05694000	−1.73750000
Mg	3.90691100	1.33818300	−2.05904800
Cl	3.86618200	1.78339900	0.26177900
Cl	5.59099400	−0.04314300	−2.97931300
C	0.45232800	0.36972400	1.77924900
H	0.83740800	1.39803100	1.73209100
H	0.01687600	0.14634400	0.79831300
H	−0.38367900	0.38519100	2.50181800

C	2.03388300	−0.33660400	3.60347800
H	1.20613200	−0.33513100	4.33553300
H	2.76887300	−1.07314400	3.95171300
H	2.51018400	0.65113000	3.68184900
S	1.92915800	−0.88636200	−2.39408800
O	3.01771700	−1.02332200	−1.22624300
C	0.41913700	−1.36044300	−1.52970600
C	0.47656700	−2.39910400	−0.59908200
C	−0.78771300	−0.73727100	−1.84675600
C	−0.69173100	−2.78956300	0.05087400
H	1.42415600	−2.86641900	−0.36072300
C	−1.95232000	−1.14528600	−1.19710700
H	−0.81408000	0.06651200	−2.57418000
C	−1.90346000	−2.16369600	−0.24513600
H	−0.65016900	−3.57389000	0.79895700
H	−2.89358100	−0.65599400	−1.42559000
H	−2.80895600	−2.46670400	0.27029700
C	1.84730800	0.82299500	−2.71842800
H	4.15030000	2.81546200	−4.09661900
H	1.13197200	1.32709500	−2.06416500
H	1.61624600	0.96457800	−3.77587300

Int4-C

Mg	2.98777300	1.58293300	4.04543600
Cl	4.82108800	0.07670700	4.49998900
C	1.50419600	2.32840000	5.39488600
H	0.72310200	1.54900400	5.32979100
N	4.56750100	4.32744000	−0.33190500
Li	5.45853200	1.19356600	2.56281400
C	8.47320200	0.23895300	4.38221200
H	9.51810400	0.33232400	4.69014300
H	7.91121700	−0.21115400	5.20174200
C	8.33539000	−0.59669200	3.08749400
H	7.44109300	−1.22318100	3.13974500
H	9.19989400	−1.23775000	2.89853100
C	7.89291800	1.61923000	4.00429300
H	8.65961400	2.40266200	3.99596800
H	7.06617200	1.92408600	4.64604800
C	8.14914300	0.46707100	2.00941300
H	9.10653700	0.90428800	1.69386800
H	7.59026400	0.14293600	1.13053100
O	7.36160200	1.48425200	2.66434300
C	6.00874700	4.29690000	−0.75289900
C	3.57520200	4.91697400	−1.30519700
H	6.26810900	5.28794400	−1.14637900
H	2.67337300	5.02719600	−0.69848300
C	6.86744800	4.04972500	0.48670900
C	6.30194300	3.25400600	−1.83014800
C	3.98439100	6.30376500	−1.80689400
C	3.21763100	3.97840800	−2.45887800
H	4.23944600	6.97019900	−0.97674100
H	4.83398100	6.26073700	−2.49520700
H	3.14764700	6.75256500	−2.35041200
H	3.96313500	3.99517700	−3.25658400
H	3.08550600	2.94964700	−2.11825500
H	2.26476300	4.29874700	−2.88871600
H	7.38408200	3.19755000	−1.98308600
H	5.95129100	2.26586600	−1.52458500
H	5.85042500	3.50696900	−2.78903400
H	6.68641700	4.80156600	1.25993400
H	6.68738100	3.06300900	0.91759900
H	7.92648300	4.10167200	0.21871000
C	1.51017500	−1.05333800	0.94698900
H	2.00442000	−0.47596900	0.16245800
H	1.38672500	−2.08179800	0.59838000
C	0.17416500	−0.38891900	1.34951400
H	−0.58214100	−1.13747800	1.60182600
H	−0.21329600	0.23777900	0.54528700
C	2.33797300	−0.97229500	2.22053900
H	3.41402800	−0.95672200	2.06518500
H	2.08028100	−1.75887000	2.94107300
C	0.52390100	0.45846200	2.58854400
H	0.33493200	1.52128300	2.45129400
H	0.02294900	0.10267000	3.49428000
O	1.95935700	0.31037700	2.77503800
Mg	3.67590800	2.61699200	0.73927500
Cl	1.42103600	2.80055400	0.32199700
Cl	4.89437700	0.62978900	0.32133000
S	5.02147300	3.97365500	3.59622100
O	4.12604100	2.65211800	2.68414700
C	4.11349900	5.33584600	2.84271200
C	4.82887000	6.51275200	2.59858100
C	2.76775300	5.22656200	2.49423000
C	4.18660600	7.58675500	1.98334900
H	5.87221900	6.58514800	2.88816700
C	2.13452600	6.30980000	1.88251500
H	2.22470000	4.30914300	2.66888700
C	2.83994900	7.48500100	1.62315000
H	4.73816000	8.50006000	1.78480000
H	1.09165500	6.21648900	1.60004400
H	2.34498800	8.31989700	1.13785900
C	4.68667600	4.13750600	5.18328200
H	3.72007300	4.48265800	5.52741600
H	4.55326300	4.98679700	0.44891600
C	1.95155300	2.36044900	6.86436000
H	2.34016500	1.38984500	7.19726400
H	2.75311600	3.09212900	7.03523100
H	1.13484100	2.63632200	7.55511600
H	5.32116300	3.57054300	5.85410300
C	0.81879000	3.65122100	5.02554200
H	1.51798300	4.49984900	5.05125400
H	0.38914900	3.63203000	4.01435300
H	−0.00157800	3.91868500	5.71509700

TS2-C

Mg	3.15678500	1.69320000	3.96090300
Cl	4.94453800	0.22159200	4.59103200
C	1.68806400	2.38507400	5.46420000
H	1.23315200	1.39640100	5.26659400
N	4.53101000	4.40799500	−0.33139000
Li	5.51096000	1.31106000	2.46766700
C	8.53027400	0.10572400	4.21696300
H	9.59961200	0.11302000	4.44604300
H	7.99062500	−0.24433900	5.09791700
C	8.22188800	−0.77807700	2.98614200
H	7.26857800	−1.29370800	3.12758800
H	8.99749600	−1.52261300	2.78991000
C	8.05561700	1.51444000	3.79638100
H	8.88965200	2.22244900	3.71791100
H	7.29035100	1.92255400	4.45759700
C	8.08762100	0.24042500	1.85798600
H	9.06702700	0.55233100	1.46856700
H	7.44777700	−0.06475500	1.02902800
O	7.45083300	1.36967500	2.49141900
C	5.99738800	4.45748100	−0.64438300
C	3.58026400	4.89147600	−1.39553000
H	6.24476900	5.48303800	−0.95008500
H	2.65257000	5.05501300	−0.84104800
C	6.78451600	4.14759600	0.62865300
C	6.40455600	3.50553700	−1.76794800
C	4.00273200	6.22184000	−2.02397000
C	3.26887800	3.83041400	−2.45304700
H	4.22123000	6.97294900	−1.25792800
H	4.88336600	6.11559900	−2.66461700
H	3.18896000	6.60485800	−2.64697700
H	4.07556800	3.70461800	−3.17666300
H	3.05342100	2.86178200	−1.99736000
H	2.37231300	4.13317800	−3.00160300
H	7.49692800	3.46630900	−1.82098500
H	6.03653300	2.49526300	−1.57400300
H	6.04393300	3.83571900	−2.74291900
H	6.49398100	4.80516900	1.45152700
H	6.64767700	3.11383400	0.94789500
H	7.85223800	4.29867000	0.44461200
C	1.55854500	−0.88029400	1.02026700
H	1.94318300	−0.16131100	0.29347000
H	1.53887000	−1.87017000	0.55750200
C	0.18350000	−0.43712300	1.54974200
H	−0.39315000	−1.29944500	1.89849400
H	−0.40002800	0.08383600	0.78849100
C	2.42103300	−0.84585000	2.27031100
H	3.48247500	−0.70639500	2.07691800
H	2.27498100	−1.72486500	2.90957100
C	0.51999300	0.49723800	2.72356400
H	0.36704800	1.54504200	2.46956000
H	−0.02610000	0.24487800	3.63720700
O	1.94565600	0.32808600	2.98005800
Mg	3.68923600	2.67297800	0.74012700
Cl	1.39397300	2.92628800	0.43572900
Cl	4.89018700	0.69969300	0.12815500
S	4.97738500	4.21066100	4.00381500
O	4.18943000	2.61291500	2.58437300
C	4.15503100	5.48010600	3.05203300
C	4.90551800	6.59581400	2.66966800
C	2.82900800	5.32680700	2.64099600
C	4.31659900	7.56953500	1.86145300

H	5.93521800	6.70019900	2.99555400
C	2.25102700	6.30745700	1.83602100
H	2.27433500	4.43554300	2.89607300
C	2.99108700	7.42556700	1.44469300
H	4.89463000	8.43640600	1.55825300
H	1.23183700	6.16953300	1.49299100
H	2.53925100	8.17917800	0.80796100
C	4.05206000	4.06315600	5.34833200
H	3.12276100	4.59739400	5.46762900
H	4.41566900	5.08002100	0.42944600
C	1.94589600	2.44518100	6.96549400
H	2.63192500	1.65629400	7.29491700
H	2.38076700	3.40470400	7.27715000
H	1.01816800	2.33753000	7.55207400
H	4.43451400	3.43840800	6.14428500
C	0.69308400	3.44934000	5.00940700
H	1.08456200	4.46801100	5.14659700
H	0.42885600	3.35696700	3.94863300
H	-0.25173000	3.41716800	5.57860400

Prod-C

Mg	3.42939200	-1.18538000	-0.23543700
Cl	3.31068600	-3.29800500	0.77770700
C	0.02204000	-0.83941200	2.05619400
H	-0.47227500	-1.61704900	2.65540700
N	5.09710500	0.95955800	-3.64006500
Li	4.65161900	-3.32157900	-1.34153600
C	8.50868000	-4.03256000	-1.57169100
H	9.07281400	-4.96828000	-1.64486300
H	9.12432400	-3.21433400	-1.94788200
C	8.04456400	-3.79414400	-0.12703400
H	7.80781400	-2.73563300	0.01653000
H	8.78703100	-4.09275800	0.61724800
C	7.18879700	-4.13352500	-2.34479300
H	7.19250200	-4.91893800	-3.10613500
H	6.91905300	-3.17699500	-2.79963700
C	6.77859800	-4.63688000	-0.05982400
H	7.00231700	-5.70199500	0.08128700
H	6.05304200	-4.32070000	0.69296600
O	6.15814700	-4.45679900	-1.36068300
C	5.71966200	2.31551600	-3.81607800
C	3.62016200	0.86714300	-3.88578700
H	5.01092900	2.94883500	-4.36306100
H	3.37472400	-0.16124000	-3.61141500
C	6.00981100	2.95804400	-2.45973800
C	6.99391200	2.18150700	-4.64970700
C	2.83329100	1.79474000	-2.96377100
C	3.25468700	1.07591000	-5.35717200
H	3.09671000	1.63642700	-1.91531600
H	3.00285100	2.84950600	-3.20159500
H	1.76473900	1.59613700	-3.08216400
H	3.45616400	2.09868300	-5.69261800
H	3.81210300	0.38870100	-6.00346900
H	2.18855300	0.87918500	-5.50482100
H	7.47167600	3.15745000	-4.77326200
H	7.70278900	1.51178800	-4.15286300
H	6.77384200	1.78610100	-5.64795500
H	5.11911000	3.00670200	-1.83068400
H	6.77828100	2.39017200	-1.92457000
H	6.38495500	3.97672100	-2.59943400
Mg	5.62118400	-0.24011100	-1.90546200
Cl	4.71861600	0.75487300	0.25905000
Cl	7.89775400	-0.49746400	-2.08613300
C	-0.84344400	0.42426700	2.11123300
H	-0.39107000	1.23878700	1.53686900
H	-1.84382100	0.23976500	1.70503700
H	-0.95684800	0.76765700	3.14373900
C	1.41287300	-0.60131500	2.65939700
H	1.33012300	-0.28761400	3.70406900
H	2.02468000	-1.50703500	2.62353100
H	1.94612700	0.20022900	2.13226400
S	1.03483300	-0.32133000	-0.51219200
O	4.34212500	-1.63628800	-1.82836900
C	0.50146700	-0.93065800	-2.11910100
C	-0.78674800	-0.59911300	-2.55547400
C	1.36748300	-1.66068500	-2.93932600
C	-1.21786200	-1.01685400	-3.81271300
H	-1.44013300	-0.01195300	-1.91787600
C	0.92642400	-2.05944000	-4.20349600
H	2.38880100	-1.87428800	-2.61925300
C	-0.36062400	-1.74639200	-4.63957300
H	-2.21747500	-0.76219700	-4.15056500
H	1.59978100	-2.61778800	-4.84670800

H	-0.69546200	-2.06362000	-5.62224600
C	0.09287500	-1.43462800	0.64390400
H	5.52432600	0.35479900	-4.34323300
H	0.59323000	-2.40499400	0.64468800
H	-0.90425500	-1.53943600	0.21026900

Complex A

Cl	-1.41535200	-1.72222800	0.64579500
O	-1.50679600	1.34738300	-0.58792900
C	-0.08076000	1.27555100	-0.30988400
C	-2.03742300	2.36541100	0.30576900
C	0.06761300	1.57354100	1.19347200
H	0.26476800	0.28271000	-0.58966300
H	0.41687700	2.02382200	-0.93559300
C	-1.33291000	2.07645400	1.62699100
H	-1.76982500	3.34642200	-0.10512300
H	-3.12008300	2.24500100	0.32122900
H	0.84119400	2.32726700	1.35944400
H	0.34572900	0.67082100	1.73910300
H	-1.28781100	2.95866900	2.26982300
H	-1.87102800	1.28856200	2.15951300
Mg	-0.64818200	-2.66231900	-1.58358100
C	1.47219500	-2.91413100	-1.60050100
H	1.83275000	-3.16222900	-2.61300400
N	-2.08618300	-1.44935200	-2.62805400
C	-3.21871700	-2.35573200	-3.00358400
C	-2.79639000	-3.48856000	-3.95771300
C	-4.46479100	-1.65439700	-3.56954500
H	-3.53821500	-2.82882700	-2.06386400
H	-1.95671300	-4.06278600	-3.55946600
H	-3.62724200	-4.18549100	-4.11229800
H	-2.51377200	-3.09751300	-4.93984100
H	-4.82704800	-0.87128700	-2.89956300
H	-4.26541200	-1.21628900	-4.55256400
H	-5.27207000	-2.38353400	-3.69731400
C	-1.61573300	-0.62520300	-3.77767400
C	-0.09195900	-0.48494800	-3.77687900
C	-2.25249200	0.77671700	-3.82386400
H	-1.88738700	-1.13479600	-4.71076100
H	0.40863900	-1.45448200	-3.84550200
H	0.24350700	0.13409300	-4.61556000
H	0.25233000	0.00009300	-2.85737400
H	-3.34208800	0.73122000	-3.77834100
H	-1.97573600	1.30112900	-4.74559700
H	-1.90044700	1.38832500	-2.98553100
Mg	-2.63320400	-0.35860700	-0.93541900
Cl	-4.75396400	0.20643500	-0.32134900
C	2.25559500	-1.67952100	-1.13118000
H	3.34253100	-1.86188700	-1.05367200
H	2.13152400	-0.82357200	-1.80710200
H	1.93131600	-1.35415700	-0.13135000
C	1.81151700	-4.10861100	-0.69205000
H	1.45693800	-3.94286200	0.33633400
H	1.34577400	-5.03898200	-1.04022100
H	2.89733300	-4.29727400	-0.61432000
S	-2.70193300	-4.69150000	-0.21971000
O	-1.52382500	-4.45679200	-1.19703600
C	-3.39382800	-6.26754500	-0.75305500
C	-2.66098700	-7.08026100	-1.61492300
C	-4.66820500	-6.62047100	-0.30931100
C	-3.21738500	-8.29079500	-2.02584900
H	-1.68653800	-6.75044100	-1.95747200
C	-5.20872800	-7.83807200	-0.72136200
H	-5.23294700	-5.95538300	0.33785400
C	-4.48412800	-8.67029500	-1.57692800
H	-2.66257300	-8.93642600	-2.69894500
H	-6.19836100	-8.12931400	-0.38502200
H	-4.91267700	-9.61274100	-1.90248100
C	-1.89218900	-5.24003200	1.31367000
H	-1.18022700	-6.02691400	1.05973000
H	-2.65713400	-5.60194800	2.00381500
H	-1.38642600	-4.36276600	1.71787400

Int1-A

Cl	-0.06041800	-1.73800700	1.40354000
O	-1.19844100	1.35469500	0.50424500
C	0.14116000	1.60343800	-0.03497800
C	-2.03645000	2.53305900	0.32563400
C	0.09462500	3.02064700	-0.61094700
H	0.86217300	1.46891400	0.77241200
H	0.32101500	0.84900400	-0.80045400
C	-1.40479800	3.25263100	-0.85655400

H	-3.05664000	2.17979200	0.17394800
H	-1.98886700	3.12882800	1.24375200
H	0.69003900	3.10140700	-1.52283400
H	0.47962900	3.74469300	0.11352900
H	-1.71731100	2.78576900	-1.79413300
H	-1.67388800	4.31101200	-0.89008300
Mg	0.10939300	-2.73341100	-0.93656900
C	1.87898400	-2.19256800	-2.04468500
H	1.58528100	-2.08225500	-3.10772400
N	-1.16560700	-4.28376400	-1.16054000
C	-2.44740900	-4.23576200	-0.44991800
C	-2.41355300	-4.80869600	0.97949800
C	-3.61579800	-4.85930900	-1.24176900
H	-2.70374200	-3.16990000	-0.33965300
H	-1.62746900	-4.32412300	1.56506000
H	-3.37100300	-4.65417600	1.49035800
H	-2.21529900	-5.88600900	0.96614300
H	-3.70173200	-4.40885600	-2.23618500
H	-3.47737000	-5.93671900	-1.37933400
H	-4.56717300	-4.71206300	-0.71930100
C	-0.62900400	-5.63152000	-1.37429600
C	0.53445500	-5.96466600	-0.42001100
C	-0.17904000	-5.79834000	-2.83382200
H	-1.41036200	-6.38456100	-1.19311900
H	0.22401500	-5.85109700	0.62159200
H	0.90305000	-6.98846900	-0.56153100
H	1.38123400	-5.28879300	-0.59529500
H	-1.03071800	-5.69065000	-3.51366000
H	0.28258400	-6.77611500	-3.01417000
H	0.55735200	-5.02838300	-3.09338300
Mg	-1.87853400	-0.55693000	0.52943300
Cl	-4.12723700	-0.38816600	0.65348400
C	2.54248200	-0.87094300	-1.62516000
H	3.48177800	-0.66878700	-2.17110500
H	1.89753500	0.00285600	-1.79052200
H	2.79733900	-0.86724400	-0.55702500
C	2.94015200	-3.30642800	-2.00938000
H	3.28310500	-3.50262800	-0.98362300
H	2.56375500	-4.25754800	-2.40279300
H	3.84468800	-3.05496900	-2.59155900
S	-2.39449100	-1.15975900	-2.53989800
O	-1.38502900	-1.20799200	-1.31205000
C	-1.81785500	0.28419000	-3.42788600
C	-0.44540600	0.50312600	-3.58074100
C	-2.76701600	1.19345500	-3.89393000
C	-0.02344400	1.66329800	-4.22312300
H	0.27111600	-0.21292700	-3.19306400
C	-2.32807500	2.34698100	-4.54742400
H	-3.82556000	1.00998600	-3.73891800
C	-0.96198600	2.58180500	-4.70683300
H	1.03844200	1.84883700	-4.34758700
H	-3.05270300	3.06430100	-4.91792200
H	-0.62479100	3.48390900	-5.20698200
C	-1.82124300	-2.52707200	-3.56026000
H	-0.87707800	-2.26791400	-4.04092100
H	-2.60006900	-2.74312800	-4.29409200
H	-1.67037500	-3.37179500	-2.82668100

TS1-A

Cl	0.03464400	-1.78990600	1.42564900
O	-1.17023700	1.38103700	0.59085000
C	0.00828400	1.72251100	-0.20758500
C	-2.06624100	2.52550800	0.64262500
C	-0.33141400	3.04614100	-0.91245700
H	0.86627700	1.79345900	0.46400000
H	0.16570100	0.89881200	-0.90243200
C	-1.84847300	3.20359600	-0.70070200
H	-3.06989500	2.13490600	0.81213500
H	-1.76542600	3.16258400	1.48223200
H	-0.06183900	3.01631000	-1.96976400
H	0.20703300	3.87651600	-0.44681100
H	-2.40139200	2.67122800	-1.47921900
H	-2.17147500	4.24723900	-0.69981300
Mg	0.10738700	-2.65456300	-0.93907000
C	1.94079600	-2.20480000	-1.97248600
H	1.74117300	-2.13313300	-3.05867000
N	-1.23662900	-4.19016000	-1.26875500
C	-2.45922200	-4.21186100	-0.44270800
C	-2.27077400	-4.77740100	0.97363400
C	-3.65750900	-4.89925700	-1.12506200
H	-2.74844800	-3.15709500	-0.31835800
H	-1.43710800	-4.28880500	1.48363900
H	-3.17603900	-4.62120400	1.57019200
H	-2.07549900	-5.85446400	0.94304500
H	-3.82048600	-4.51048200	-2.13420000
H	-3.51596600	-5.98189800	-1.20180100
H	-4.56990400	-4.72984900	-0.54458500
C	-0.68556200	-5.50758600	-1.62947100
C	0.44305700	-5.95577000	-0.68318600
C	-0.17561000	-5.47569300	-3.07702900
H	-1.47400900	-6.27131000	-1.57910500
H	0.10564500	-5.97895800	0.35469300
H	0.81005300	-6.95498500	-0.94642200
H	1.29556200	-5.26968000	-0.74648200
H	-0.99495200	-5.28841300	-3.77805200
H	0.29898600	-6.42294300	-3.35307800
H	0.56826000	-4.68069900	-3.20491000
Mg	-1.79627800	-0.54677600	0.63986000
Cl	-4.01530200	-0.48008700	1.04662800
C	2.55406000	-0.86248700	-1.53679300
H	3.52763400	-0.65997500	-2.01805400
H	1.90955400	-0.00439900	-1.76882800
H	2.73145600	-0.83365800	-0.45359600
C	3.00321800	-3.30627600	-1.81040500
H	3.24780700	-3.48060600	-0.75296300
H	2.67642800	-4.26734700	-2.22377600
H	3.95658800	-3.05392600	-2.30727500
S	-2.39951800	-1.05839500	-2.45304400
O	-1.36529200	-1.14718500	-1.21898000
C	-1.64248100	0.25169600	-3.41336600
C	-0.26540600	0.24826600	-3.65435500
C	-2.46550500	1.27496000	-3.88464100
C	0.28955800	1.29541200	-4.38437100
H	0.35911600	-0.54856400	-3.26657700
C	-1.89601400	2.31649500	-4.61995100
H	-3.53060900	1.26067800	-3.67473700
C	-0.52285100	2.32753300	-4.86708400
H	1.35817900	1.30583300	-4.57247200
H	-2.52452700	3.11910300	-4.99159900
H	-0.08274400	3.14073800	-5.43521800
C	-2.08502300	-2.55619900	-3.33463300
H	-1.27207500	-2.42697000	-4.05047200
H	-3.01127100	-2.85561100	-3.82652600
H	-1.73505600	-3.39699700	-2.45905600

Int2-A

Cl	-0.38460100	-1.76616100	1.14418000
O	-1.50069000	1.34286100	0.36325800
C	-0.34183700	1.62435200	-0.47537200
C	-1.98751500	2.55595500	1.00571500
C	0.11347900	3.01206200	-0.04234300
H	0.38875700	0.83513000	-0.29596800
H	-0.66423800	1.59707900	-1.51906100
C	-1.21299100	3.68802800	0.33624700
H	-3.06817600	2.59580400	0.86417900
H	-1.77070200	2.47806800	2.07535200
H	0.64809500	3.53275000	-0.83986400
H	0.77382100	2.94308400	0.82798700
H	-1.73416400	4.01887500	-0.56584200
H	-1.08600800	4.54549400	1.00101500
Mg	-0.43254800	-2.37407700	-1.27536100
C	1.24439800	-2.00224100	-2.54745500
H	1.01832000	-2.55619600	-3.47777700
N	-1.36721300	-4.34237100	-1.46889600
C	-2.71264100	-4.47898900	-0.81690200
C	-2.60970100	-4.77770300	0.67616700
C	-3.59765300	-5.49288100	-1.54781300
H	-3.16902700	-3.49094500	-0.92391300
H	-1.98820700	-4.03984700	1.18732800
H	-3.60948900	-4.74156300	1.11736300
H	-2.20134600	-5.77549500	0.86571700
H	-3.69827500	-5.23591800	-2.60809500
H	-3.19911900	-6.51031800	-1.47841900
H	-4.60027800	-5.50017600	-1.11056800
C	-0.44417200	-5.51474000	-1.33842600
C	0.56079100	-5.29161700	-0.20629500
C	0.26760900	-5.73692600	-2.67337200
H	-1.03709100	-6.40901000	-1.11068700
H	0.06869700	-5.07954100	0.74350100
H	1.18525200	-6.18107000	-0.07769200
H	1.22522000	-4.45339000	-0.43940200
H	-0.44810700	-5.96744700	-3.47012700
H	0.96901200	-6.57229400	-2.59849000
H	0.83209700	-4.84517800	-2.96235400
Mg	-2.31430600	-0.50344600	0.46095600
Cl	-4.35974900	-0.22008100	1.38850600

C	1.34195800	−0.51904100	−2.94303200
H	2.10571300	−0.32801200	−3.71756200
H	0.39402500	−0.11630600	−3.32321300
H	1.62577900	0.10356300	−2.08313000
C	2.61859600	−2.48351800	−2.05877400
H	2.89443100	−2.00993500	−1.10646200
H	2.65001000	−3.56773100	−1.89127200
H	3.43328000	−2.25254000	−2.76785200
S	−3.05714700	−1.28762200	−2.74715900
O	−2.12461900	−1.29405500	−1.31169500
C	−2.92060400	0.50494500	−2.96847400
C	−2.16764700	1.06307800	−3.99858100
C	−3.64337600	1.31417400	−2.08676200
C	−2.09404600	2.45182700	−4.11713800
H	−1.65812800	0.41998300	−4.70654300
C	−3.58386100	2.69951700	−2.22902200
H	−4.24738900	0.87366500	−1.29770400
C	−2.80036500	3.27078100	−3.23468600
H	−1.49595700	2.89108400	−4.90926100
H	−4.15584700	3.33096600	−1.55624900
H	−2.75592900	4.35013000	−3.34211400
C	−2.27969700	−2.03737900	−3.97508300
H	−1.31099500	−1.72918600	−4.34665800
H	−2.74351100	−2.95089400	−4.32001900
H	−1.57215600	−4.22956500	−2.46460700

TS2-A

Cl	0.47098200	−2.02441200	0.90693700
O	−0.90771500	1.25750400	0.91959000
C	−0.41868300	1.86262900	−0.31852200
C	−1.51410000	2.27775900	1.75055200
C	−0.97299400	3.29606200	−0.33212800
H	0.67384900	1.82880400	−0.30496300
H	−0.79463300	1.23269600	−1.12677800
C	−2.07413300	3.27014300	0.74154600
H	−2.25598200	1.77918100	2.37495300
H	−0.73623400	2.72902600	2.37885500
H	−1.35131200	3.56866600	−1.31933100
H	−0.19435700	4.01369200	−0.05542600
H	−3.00949000	2.87986200	0.32827700
H	−2.27149500	4.25221000	1.17836200
Mg	−0.37498700	−2.26759700	−1.38169200
C	1.38559300	−1.92424900	−2.81929600
H	1.45899900	−2.07235400	−3.89684300
N	−1.48222800	−4.18305600	−1.50180600
C	−2.73277100	−4.03472600	−0.66219600
C	−2.49794900	−4.36642700	0.80757700
C	−3.89678700	−4.83378300	−1.25168600
H	−2.95945800	−2.96610600	−0.73485800
H	−1.64462500	−3.81969300	1.21262600
H	−3.37733000	−4.06515300	1.38174400
H	−2.33912600	−5.43836900	0.96717600
H	−4.09131800	−4.54543100	−2.29161500
H	−3.71687200	−5.91415000	−1.22314600
H	−4.80779700	−4.63703900	−0.67925500
C	−0.80535300	−5.51700900	−1.45609000
C	0.32592600	−5.54380500	−0.42876500
C	−0.28608600	−5.84363100	−2.85730500
H	−1.54604000	−6.27819200	−1.18004900
H	−0.01259100	−5.25017100	0.56365300
H	0.74242500	−6.55422400	−0.36593200
H	1.13271800	−4.86566500	−0.71614700
H	−1.10711600	−5.91830700	−3.57993800
H	0.24927200	−6.79670700	−2.86042100
H	0.40776600	−5.06820300	−3.20010900
Mg	−1.63542200	−0.66332700	0.75560500
Cl	−3.25166600	−0.79266400	2.37068100
C	1.63885700	−0.46224800	−2.46118200
H	2.66748400	−0.15537600	−2.71577700
H	0.95258600	0.21818500	−2.97692300
H	1.52288700	−0.28158800	−1.38558400
C	2.38522100	−2.86898000	−2.14423100
H	2.34074500	−2.83225800	−1.04979900
H	2.24677300	−3.90924400	−2.45576500
H	3.41781700	−2.59135000	−2.41860700
S	−2.43141100	−1.46325000	−3.43134700
O	−1.77764100	−1.03943100	−1.07911000
C	−2.58617900	0.31078700	−3.53349300
C	−2.13290600	1.01055700	−4.65370400
C	−3.28597700	0.95759700	−2.51036000
C	−2.35170800	2.38638100	−4.73244900
H	−1.62638700	0.49196200	−5.46038500
C	−3.53184000	2.32387700	−2.62239500
H	−3.57500900	0.38647100	−1.63741700
C	−3.05692600	3.04230700	−3.72331700
H	−1.98837700	2.93808600	−5.59353300
H	−4.08442500	2.83248500	−1.83909500
H	−3.24271300	4.10922800	−3.79707100
C	−0.93409700	−1.82910600	−4.06498000
H	−0.29952000	−1.04339500	−4.44384600
H	−0.79249200	−2.83662700	−4.43183800
H	−1.82741700	−4.09100600	−2.45770300

Prod-A

Cl	0.55098700	−2.72826600	0.80269400
O	0.28673500	1.00972900	1.44887900
C	−0.21217700	2.16751600	0.73070500
C	0.61740300	1.46463900	2.78634800
C	−1.07822800	2.89418100	1.75996300
H	0.64768900	2.76896900	0.40901800
H	−0.74668000	1.78203200	−0.13841800
C	−0.40646800	2.56545400	3.11935700
H	0.55596000	0.59727300	3.44298900
H	1.64504600	1.84485300	2.76438100
H	−2.09288800	2.48866000	1.73805700
H	−1.13433300	3.96719500	1.56061500
H	−1.14542100	2.19198700	3.82904400
H	0.08926700	3.43722700	3.55413200
Mg	−0.70174900	−2.24320400	−1.15167100
C	1.71677100	0.35444000	−2.91752900
H	2.48237100	0.97592100	−3.40886000
N	−2.30793200	−3.56323600	−1.72493000
C	−3.40142000	−3.22621500	−0.73731300
C	−3.17178600	−3.90097400	0.61177400
C	−4.78574700	−3.52189400	−1.31577900
H	−3.29386300	−2.14370900	−0.59643900
H	−2.17691400	−3.68595300	1.00970700
H	−3.89635000	−3.51633100	1.33339300
H	−3.29701600	−4.98745500	0.55490600
H	−4.93717700	−2.99141100	−2.26269600
H	−4.94120300	−4.59201100	−1.49205500
H	−5.55937700	−3.18730000	−0.61869000
C	−2.09647700	−5.01586500	−2.01938900
C	−0.85320800	−5.53482400	−1.29801500
C	−1.99003300	−5.21896400	−3.53112300
H	−2.96882500	−5.57394400	−1.66052900
H	−0.89817400	−5.35014500	−0.22357800
H	−0.73995400	−6.61140800	−1.45853800
H	0.04972100	−5.04652500	−1.68423000
H	−2.89270800	−4.87235700	−4.04515200
H	−1.85093900	−6.27800300	−3.76491900
H	−1.13381400	−4.67163600	−3.93864900
Mg	−0.96673100	−0.61223500	1.15669400
Cl	−2.11192500	−0.68742200	3.13723000
C	0.43812400	1.18373300	−2.77121200
H	0.67355800	2.14419700	−2.30135700
H	−0.02585200	1.39092400	−3.74122300
H	−0.28132600	0.66376400	−2.12755400
C	2.26464600	−0.06371400	−1.55011300
H	1.53522700	−0.64903000	−0.98541700
H	3.18398400	−0.65207500	−1.64062100
H	2.48606000	0.81900200	−0.94302800
S	0.31816300	−2.15113500	−3.51539200
O	−1.43971000	−0.62059000	−0.65259900
C	−1.18644500	−1.58149500	−4.33474500
C	−1.38774200	−1.97113900	−5.66424300
C	−2.16273700	−0.84784500	−3.64529700
C	−2.56773400	−1.61733100	−6.31713500
H	−0.62866800	−2.55368300	−6.17610700
C	−3.34519200	−0.51511800	−4.31105800
H	−2.01540500	−0.55701000	−2.59633000
C	−3.54937200	−0.89237200	−5.63912700
H	−2.72317600	−1.91747800	−7.34864300
H	−4.10657900	0.04822400	−3.78028700
H	−4.47214000	−0.62554400	−6.14510600
C	1.57191000	−0.82773000	−3.88198300
H	1.31304400	−0.48944200	−4.88868000
H	2.51100600	−1.38540100	−3.94480100
H	−2.60785100	−3.12265300	−2.59540100

Complex B

Mg	1.30809300	1.30636400	−0.70167600
Cl	1.73994800	2.73279700	1.35671400
N	2.72628300	−0.08417000	0.05718000
C	3.81163000	4.84967600	3.65486600

H	3.18441400	3.96477500	3.53557900
H	3.29075200	5.54954600	4.31311300
C	4.13842400	5.46516000	2.26780400
H	3.79067300	6.49552400	2.16884000
H	3.67464000	4.87801600	1.47410400
C	5.18247400	4.46315700	4.22595400
H	5.18760100	3.51118700	4.75772900
H	5.59607500	5.24901900	4.87187900
C	5.66643000	5.38016600	2.17146100
H	6.02890200	5.10913100	1.18091900
H	6.15176300	6.30839800	2.50258700
O	6.04290700	4.31887800	3.07452500
C	8.45610100	0.05163100	0.79842200
H	7.72084100	0.02421600	−0.00721300
H	9.36256000	−0.44868100	0.44823000
C	7.88777300	−0.60141100	2.08554200
H	8.36127600	−1.55724600	2.32257300
H	6.81225300	−0.76454000	1.99025100
C	8.75785200	1.50473700	1.21598000
H	8.38732000	2.24944300	0.51126100
H	9.82967800	1.66397700	1.38886800
C	8.14194900	0.45061900	3.16583900
H	7.39183000	0.46567500	3.95646600
H	9.14444300	0.35779000	3.60505300
O	8.05862800	1.70815900	2.46261100
C	3.46786300	−0.88507500	−0.95092800
C	1.96996900	−0.92553400	1.03865800
H	3.01786800	−1.88432000	−1.01195800
H	1.54235800	−0.21610000	1.76319700
C	3.34387100	−0.25088800	−2.34045700
C	4.95391700	−1.07260700	−0.60125400
C	0.78407800	−1.67140500	0.40156800
C	2.83378400	−1.91123000	1.84091700
H	0.11523000	−0.98512800	−0.12478900
H	1.12122400	−2.42641600	−0.31620500
H	0.19770900	−2.18740500	1.16878300
H	3.24672500	−2.69430800	1.19652500
H	3.65935300	−1.40359300	2.34631600
H	2.22578700	−2.40470300	2.60627600
H	5.44283800	−1.75316800	−1.30826100
H	5.47730500	−0.11225200	−0.64808600
H	5.07887100	−1.48388600	0.40291600
H	2.29858900	−0.22269300	−2.67185100
H	3.73261500	0.77270000	−2.33638400
H	3.90623800	−0.82014500	−3.08814600
Cl	5.55603800	2.52986700	0.12755400
Cl	4.68649600	1.08137600	3.26309400
Mg	3.71992400	1.38673600	1.12593400
Li	6.32459000	2.55110600	2.37323000
C	1.20503600	4.45846700	−3.52098800
H	2.14211700	4.35407400	−4.07022500
S	1.51972200	4.09068600	−1.76771200
O	2.26985100	2.73356900	−1.83574300
C	2.78446500	5.32930200	−1.44426900
C	4.12323900	5.02498700	−1.67174600
C	2.38118700	6.56564100	−0.93872300
C	5.08284300	6.00105200	−1.40543100
H	4.40095000	4.03354400	−2.00672700
C	3.35010900	7.53593600	−0.68809800
H	1.33349900	6.76194400	−0.73073200
C	4.69781900	7.25322400	−0.92312900
H	6.13176600	5.77480100	−1.56586200
H	3.05511500	8.50391800	−0.29626000
H	5.45048800	8.00746500	−0.71592500
H	0.47016100	3.72685100	−3.86122700
H	0.80818200	5.47223800	−3.60595800
C	−0.66662500	1.26953300	−1.53179200
H	−0.79473200	2.29801700	−1.92457900
C	−1.83463700	1.03803200	−0.56309400
H	−1.79358600	0.04023200	−0.10479700
H	−1.83385100	1.76220800	0.26083000
H	−2.82146400	1.10500600	−1.05511300
C	−0.78076300	0.32634900	−2.74025200
H	−1.76644000	0.37178400	−3.23742000
H	−0.02702600	0.54407800	−3.51040500
H	−0.63754400	−0.72396400	−2.44859700

Int1-B

Mg	0.17352800	1.46383500	1.10951700
Cl	1.44542700	3.30522100	2.39301100
N	−0.35780900	1.77694300	−0.79693300
C	4.17516200	4.61466000	4.89075000
H	3.30998000	3.97374400	4.70764400
H	3.89284500	5.34287900	5.65462000
C	4.64393600	5.28824700	3.57217000
H	4.58983000	6.37850700	3.61508100
H	4.02661600	4.95866800	2.73339300
C	5.38574100	3.78198500	5.33687600
H	5.12524200	2.80358000	5.74023600
H	6.01217100	4.32524300	6.05611400
C	6.08986600	4.80551400	3.39972700
H	6.36705500	4.59091500	2.36748800
H	6.81060000	5.50796200	3.83791200
O	6.15753300	3.56308700	4.13490300
C	7.86849800	−1.68743400	2.60056000
H	7.48730700	−2.48755800	1.96430600
H	8.95450000	−1.79993400	2.68842500
C	7.21641600	−1.65680500	3.98887200
H	7.62400900	−2.39894200	4.67962800
H	6.13576100	−1.80717700	3.90358800
C	7.51006600	−0.30808500	2.05479800
H	6.52798400	−0.30547200	1.57145900
H	8.24681500	0.10512300	1.36234900
C	7.50391100	−0.22717200	4.43339200
H	6.75988300	0.16879500	5.12799800
H	8.50441100	−0.12456400	4.86945600
O	7.44984900	0.56614600	3.21811300
C	0.10962000	2.87432300	−1.64392500
C	−1.70192600	1.26814700	−1.10168100
H	−0.41208300	2.85214100	−2.61420900
H	−1.90940500	0.49860800	−0.34103300
C	−0.12261500	4.27533700	−1.04037300
C	1.60761500	2.70975300	−1.94558400
C	−2.84135300	2.29987000	−0.98584100
C	−1.79385300	0.55277900	−2.46686800
H	−2.81418800	2.79545400	−0.01017800
H	−2.76159000	3.07170500	−1.75899100
H	−3.82081800	1.82029700	−1.10017800
H	−1.59040100	1.24027100	−3.29475600
H	−1.07241900	−0.26816600	−2.52989500
H	−2.79400100	0.13449200	−2.62844000
H	1.99420800	3.53169100	−2.55798600
H	2.18573900	2.70485100	−1.01323800
H	1.80175000	1.77325600	−2.47958900
H	−1.17732300	4.43855200	−0.81061300
H	0.44404100	4.38255100	−0.10904000
H	0.20332100	5.06846900	−1.72589700
Cl	5.20947700	2.33720600	1.02460300
Cl	4.13296500	0.76997900	4.05801100
Mg	3.23695200	1.83868000	2.19122400
Li	6.08456700	1.89419300	3.19197800
C	1.41031600	−0.84520100	−1.04799200
H	0.96811400	−1.56355500	−0.35698200
S	2.86583600	−0.09564800	−0.28927100
O	2.22021200	0.66866400	0.93513800
C	3.65920400	−1.50771900	0.48608300
C	3.24665100	−1.93356400	1.74813800
C	4.69730200	−2.13289700	−0.20597100
C	3.88337400	−3.03404600	2.31891600
H	2.45956200	−1.40585400	2.27251500
C	5.31110400	−3.24439700	0.37055300
H	5.02128100	−1.75937500	−1.17273000
C	4.90405700	−3.69257000	1.62962000
H	3.57791800	−3.37408000	3.30256100
H	6.11189300	−3.75149500	−0.15774200
H	5.39103100	−4.55246100	2.07834200
H	0.72923200	0.01992900	−1.21611800
H	1.72577600	−1.31781700	−1.98063700
C	−0.67176200	0.07072500	2.50040800
H	−1.70197500	0.39842800	2.71837700
C	0.08338000	0.04398100	3.83775300
H	1.13827100	−0.24515600	3.71144500
H	0.09560200	1.02588000	4.32482300
H	−0.33970400	−0.67463900	4.56330200
C	−0.77573500	−1.35401300	1.93584500
H	−1.24788700	−2.06760000	2.63551200
H	−1.35223600	−1.39754900	1.00196900
H	0.21712600	−1.77701500	1.71507900

TS1-B

Mg	0.27208700	1.28233500	1.11951600
Cl	1.15103100	3.27241600	2.42884900
N	−0.05685700	1.56448400	−0.89408500
C	4.51586400	4.94529600	4.90956900
H	3.65556500	4.37428700	4.55075400
H	4.15516600	5.64503900	5.66699600

C	5.23763400	5.65478600	3.73116700
H	5.35337100	6.72785600	3.90126300
H	4.68747500	5.51469000	2.79921700
C	5.57908100	3.99833300	5.47456800
H	5.18332000	3.05334700	5.84559400
H	6.18280100	4.48172700	6.25389400
C	6.60609600	4.95610500	3.65418300
H	6.92712200	4.72578200	2.63796500
H	7.38890400	5.53104100	4.16474300
O	6.43623700	3.70043600	4.35033100
C	7.12656200	−1.55536700	2.26710500
H	6.44654100	−2.21974600	1.73230500
H	8.15032900	−1.91396000	2.11577600
C	6.80982600	−1.45543900	3.76562100
H	7.20552500	−2.29113100	4.34800800
H	5.73005500	−1.39355400	3.92135200
C	6.99130400	−0.10474500	1.80274300
H	5.97829700	0.12406500	1.46144100
H	7.69597100	0.17647200	1.01660900
C	7.45878900	−0.12726000	4.13309200
H	6.99397600	0.36803800	4.98934300
H	8.53521800	−0.23012500	4.31670500
O	7.26427400	0.71985400	2.97221400
C	0.30119500	2.80174400	−1.60172000
C	−1.29101000	0.86959500	−1.31914800
H	0.16755200	2.66387200	−2.68574500
H	−1.14917300	−0.17448600	−0.99444000
C	−0.53427500	4.02739800	−1.19335400
C	1.79006400	3.10906500	−1.38526900
C	−2.57853500	1.34667100	−0.62144800
C	−1.49798400	0.81479000	−2.84052000
H	−2.44992700	1.35537000	0.46563200
H	−2.86708600	2.35222600	−0.93854900
H	−3.41249500	0.67212200	−0.84922900
H	−1.77325500	1.79105500	−3.25228800
H	−0.59177700	0.47512600	−3.35310300
H	−2.30694500	0.11855100	−3.08400400
H	2.08092000	4.00773100	−1.93772300
H	2.00813100	3.29461800	−0.32969200
H	2.42943800	2.29210100	−1.73275700
H	−1.59043100	3.89705500	−1.43826400
H	−0.45215600	4.19887400	−0.11435700
H	−0.18251300	4.92972800	−1.70792200
Cl	5.08353600	2.94583100	1.22313500
Cl	4.08771000	1.11881100	4.15567000
Mg	3.16585100	2.10424300	2.25005600
Li	6.05126400	2.16526600	3.25859900
C	1.80875400	−0.45275200	−1.26267100
H	1.31980000	−1.38256800	−0.97004700
S	3.10513500	−0.04280800	−0.12810500
O	2.35259400	0.74824000	1.04046100
C	3.51826400	−1.60197100	0.68110200
C	3.25652900	−1.79068800	2.03518500
C	4.13209300	−2.58969900	−0.09340500
C	3.60438500	−3.00967700	2.62079800
H	2.78699900	−1.00898800	2.61560200
C	4.47245900	−3.80091500	0.50490200
H	4.32944300	−2.42254700	−1.14812900
C	4.20808500	−4.01129800	1.86167200
H	3.39381900	−3.16929000	3.67297300
H	4.94237800	−4.57933300	−0.08736800
H	4.47332500	−4.95682600	2.32342200
H	1.00256300	0.50069400	−1.19252300
H	2.24660900	−0.51373500	−2.26044200
C	−0.64141600	−0.19101000	2.36109000
H	−1.71612500	0.02921800	2.46923800
C	−0.04095300	−0.19094900	3.77580700
H	1.04553500	−0.36416500	3.76609600
H	−0.19047100	0.76779200	4.28623700
H	−0.46531000	−0.97725400	4.42626900
C	−0.53393900	−1.59415200	1.74263300
H	−0.95867700	−2.38612600	2.38612300
H	−1.05145100	−1.66331800	0.77622600
H	0.51201100	−1.88378200	1.56430400

Int2-B

Mg	0.36878400	0.85944200	1.34221100
Cl	0.78009300	2.95742200	2.68870200
N	0.31170000	1.34791900	−0.80467000
C	5.17094700	4.93614600	5.56764100
H	4.28312600	4.30750200	5.65511600
H	5.23472300	5.56409500	6.45973700
C	5.12868300	5.77139300	4.25884500
H	5.05498900	6.84579400	4.44350400
H	4.28023500	5.47358900	3.63867200
C	6.42968200	4.06494600	5.41334300
H	6.28725600	3.02797500	5.71806200
H	7.28909400	4.49024300	5.94720200
C	6.43452600	5.40412800	3.54790300
H	6.35722700	5.37582300	2.46109800
H	7.26293500	6.06148200	3.84385900
O	6.72664700	4.06409800	3.99973200
C	7.00114600	−0.80831100	1.02329800
H	6.25489900	−1.21899500	0.33968300
H	7.97154600	−1.24468600	0.76654400
C	6.66770900	−1.08065600	2.49915200
H	7.01800500	−2.05680800	2.84326600
H	5.59145000	−1.01850100	2.67642700
C	7.06267400	0.72681600	0.94533100
H	6.16073800	1.16613900	0.51555000
H	7.93605100	1.08712600	0.39337300
C	7.36515500	0.07732600	3.19804000
H	6.93298900	0.33384900	4.16692000
H	8.44342700	−0.09768400	3.30928400
O	7.15437800	1.20373500	2.31795500
C	0.01501800	2.74101500	−1.26700400
C	−0.36618000	0.23728200	−1.55008400
H	−0.27644800	2.70456900	−2.32406800
H	−0.00860600	−0.67459000	−1.05823900
C	−1.12791900	3.37416900	−0.47006000
C	1.28978700	3.57852000	−1.15250300
C	−1.88427300	0.27086600	−1.39846600
C	0.05863100	0.16629400	−3.02116200
H	−2.17467900	0.32622700	−0.34693600
H	−2.32441600	1.11480900	−1.93742900
H	−2.30996800	−0.64507800	−1.81799800
H	−0.32386900	1.01196000	−3.60038500
H	1.14826600	0.14871800	−3.11527900
H	−0.33550000	−0.74687300	−3.47741200
H	1.10938500	4.59484800	−1.51308000
H	1.62106100	3.64932400	−0.11303900
H	2.10604900	3.14982900	−1.74309500
H	−2.03201300	2.76418600	−0.48933700
H	−0.83424700	3.52766000	0.57156800
H	−1.37391000	4.35290700	−0.89385000
Cl	4.59413600	3.49277300	1.23757400
Cl	4.29949400	1.55246800	4.26016900
Mg	3.01494500	2.21985000	2.41575300
Li	6.02182300	2.61160600	2.94087500
C	3.12982500	−0.62221300	−1.03186900
H	2.28235600	−1.24078700	−1.30100900
S	3.48973500	−0.27643800	0.52887400
O	2.37013400	0.69179100	1.39107500
C	3.13344400	−1.80392700	1.42822100
C	2.82118800	−1.74236100	2.78924600
C	3.26076400	−3.03230300	0.77838200
C	2.60378500	−2.92583500	3.49198800
H	2.73885900	−0.78554500	3.28620900
C	3.02926800	−4.20946700	1.49064500
H	3.52882600	−3.05520000	−0.27118500
C	2.69909000	−4.15839500	2.84480700
H	2.34714400	−2.87805100	4.54507400
H	3.10816000	−5.16599200	0.98347800
H	2.51849500	−5.07633600	3.39516000
H	1.31055400	1.18834400	−0.96415800
H	3.62108800	−0.00451500	−1.77183000
C	−0.98298000	−0.59492900	2.11003300
H	−2.02249000	−0.26525200	1.94078500
C	−0.79509100	−0.70019600	3.63423200
H	0.22301700	−1.02217900	3.89256300
H	−0.96320100	0.25806700	4.14077900
H	−1.47417000	−1.43763600	4.09803200
C	−0.84176500	−1.98872700	1.47536400
H	−1.48958500	−2.74262900	1.95731200
H	−1.10394400	−1.99133800	0.40918800
H	0.18371800	−2.37300600	1.55446000

TS2-B

Mg	0.68152100	0.22887000	0.64906200
Cl	0.23123900	2.00140500	2.31750100
N	0.26532000	0.81434600	−1.44128100
C	5.11833900	3.84042800	6.43619300
H	4.58643100	2.91490300	6.66066900
H	5.42241000	4.30207100	7.37955800
C	4.23549900	4.78982300	5.58945800
H	3.81349900	5.61230300	6.17229300

H	3.41594800	4.23083600	5.13188800
C	6.34870100	3.57252100	5.54219600
H	6.55372700	2.51164500	5.38661100
H	7.25142500	4.05847900	5.93201000
C	5.18646700	5.27839800	4.49930800
H	4.70044200	5.53033600	3.55676500
H	5.80556500	6.12039000	4.83870000
O	6.04229100	4.14206200	4.24922100
C	6.18814400	1.81470800	-0.42270400
H	5.28560100	2.38252500	-0.65576400
H	6.76639500	1.69040000	-1.34224800
C	5.81181700	0.46102300	0.23800100
H	6.14904700	-0.40005100	-0.34606200
H	4.72735900	0.39657400	0.36666800
C	7.01025100	2.54381000	0.65185300
H	6.77346700	3.60504000	0.74606500
H	8.09019100	2.42561100	0.49130700
C	6.50563700	0.50694800	1.60543400
H	5.92355900	0.07005400	2.41595700
H	7.50194000	0.04484800	1.57875800
O	6.66005500	1.91220300	1.90045100
C	0.94309400	2.05402700	-1.96808700
C	-1.15048200	0.57942100	-1.86594600
H	0.49159300	2.30327500	-2.93675000
H	-1.41630600	-0.36995800	-1.38622200
C	0.79475900	3.25355300	-1.03325200
C	2.42338800	1.72453900	-2.17367600
C	-2.09482400	1.65298200	-1.33058900
C	-1.30395400	0.38817100	-3.37933600
H	-1.94534000	1.82144500	-0.26137400
H	-1.95682500	2.60335100	-1.85313600
H	-3.13071100	1.33765800	-1.48494800
H	-1.11059000	1.31099900	-3.93313700
H	-0.62220900	-0.38375100	-3.75427400
H	-2.32525300	0.07270100	-3.61276600
H	2.96395500	2.61249400	-2.51213900
H	2.84730000	1.38355500	-1.22199600
H	2.55770300	0.94125600	-2.93075500
H	-0.23729700	3.44401300	-0.74074800
H	1.38245700	3.11684500	-0.12575700
H	1.17625500	4.14651700	-1.53768200
Cl	3.78669200	3.84112100	1.53598300
Cl	3.95955700	1.31165300	4.07887100
Mg	2.68251300	1.81842500	2.12123300
Li	5.38235100	2.85935000	2.97894600
C	1.31462300	-2.33620100	-0.35875300
H	1.07963400	-3.07747300	0.38819500
S	2.84583700	-1.68998700	-0.43987900
O	2.55081400	0.44284400	0.84213900
C	3.68287600	-2.49869000	0.93365700
C	3.81625000	-1.84353500	2.15543000
C	4.21962800	-3.77271400	0.72284000
C	4.49217700	-2.48414900	3.19380800
H	3.38984100	-0.85097600	2.24381800
C	4.89627100	-4.39923200	1.76895200
H	4.11186000	-4.26516000	-0.23804500
C	5.03227800	-3.75654200	3.00205000
H	4.59175600	-1.97317700	4.14603800
H	5.31687100	-5.38862800	1.61889200
H	5.56067900	-4.25065500	3.81167300
H	0.79022300	0.04589500	-1.86089300
H	0.68314600	-2.25292400	-1.23446100
C	-0.65220200	-1.51185600	1.30167000
H	-0.86986100	-2.43263200	0.75760800
C	-1.97320600	-0.74658700	1.46280700
H	-1.84845800	0.22826800	1.94571300
H	-2.48624200	-0.58838200	0.50862900
H	-2.66627900	-1.32003800	2.10243900
C	-0.01313100	-1.82966800	2.65268100
H	-0.70566300	-2.38678800	3.30613100
H	0.90008000	-2.42643900	2.55676200
H	0.25690600	-0.91820200	3.19991100

Prod-B

Mg	1.52107700	0.46440700	0.16978400
Cl	0.61067700	1.55096000	2.09337500
N	0.69608900	0.75723500	-1.78606500
C	5.07465300	3.46558900	6.67048600
H	4.57787200	2.49512200	6.64267700
H	5.28324700	3.72012000	7.71327300
C	4.21028900	4.54581600	5.97495500
H	3.70702000	5.21051700	6.68161300
H	3.45522900	4.07605500	5.34017000
C	6.37986300	3.44049400	5.84571700
H	6.65297900	2.44445900	5.49413900
H	7.22218500	3.87405200	6.39870400
C	5.21333800	5.28580800	5.09407900
H	4.78219200	5.72270300	4.19346100
H	5.76953900	6.05115300	5.65306800
O	6.13389200	4.25414300	4.67564200
C	6.11657400	2.57852600	-0.52591200
H	5.03814600	2.74866500	-0.49518400
H	6.47727600	2.76897700	-1.54060600
C	6.40187400	1.13593000	-0.03622300
H	7.08813100	0.59844200	-0.69682800
H	5.44909000	0.60403900	0.02911300
C	6.81738700	3.46648200	0.50100400
H	6.30976000	4.41326000	0.69183100
H	7.86745100	3.65583700	0.23713700
C	7.02807000	1.32652400	1.35797900
H	6.57459100	0.70915300	2.13406400
H	8.11406900	1.16538000	1.34845000
O	6.77721600	2.70441200	1.72528100
C	1.25626200	2.02234100	-2.39221200
C	-0.77021900	0.49689700	-1.95197800
H	0.63566000	2.29322900	-3.25492000
H	-0.93920700	-0.44796300	-1.42010000
C	1.27302300	3.17785000	-1.38855100
C	2.68206200	1.72021600	-2.85868300
C	-1.62835800	1.56628000	-1.28089200
C	-1.16642300	0.27767000	-3.41544900
H	-1.32460900	1.74150000	-0.24557000
H	-1.57700300	2.51499600	-1.82183900
H	-2.67284400	1.24231000	-1.27869200
H	-1.05979500	1.19152100	-4.00684000
H	-0.55560200	-0.50428900	-3.87949200
H	-2.21325800	-0.03393700	-3.47657700
H	3.12932300	2.61604500	-3.29786900
H	3.28653200	1.41692400	-1.99467200
H	2.69903000	0.92977100	-3.61876400
H	0.30696900	3.34833000	-0.91333100
H	2.01941800	2.98564000	-0.61154700
H	1.56295200	4.09861400	-1.90316300
Cl	3.71746800	4.26456300	2.03865100
Cl	4.44849400	1.27009400	3.87224800
Mg	3.17850000	1.95289200	1.97723500
Li	5.58339000	3.24466700	3.12275800
C	-0.14412400	-3.01459800	0.47272200
H	0.14445100	-4.05103500	0.65337400
S	1.40981700	-2.15037900	-0.03340800
O	3.28072500	0.93778700	0.40976300
C	2.65651300	-2.75970800	1.12912600
C	3.58931300	-1.85791400	1.64605500
C	2.71021100	-4.11787400	1.45631300
C	4.55159200	-2.32518600	2.54310500
H	3.58143500	-0.80800000	1.33068400
C	3.67454800	-4.56579300	2.35767100
H	2.01689800	-4.82867300	1.01986100
C	4.59101800	-3.66921900	2.91060300
H	5.25192300	-1.61325800	2.96745800
H	3.70980700	-5.61833300	2.62189400
H	5.33442400	-4.02134600	3.61910700
H	1.15832200	-0.00824300	-2.28043900
H	-0.75231300	-3.00758500	-0.43719200
C	-0.94476400	-2.45850300	1.66019000
H	-1.68367100	-3.24809100	1.86566500
C	-1.72440100	-1.19323600	1.29390500
H	-1.05705400	-0.34996400	1.10220200
H	-2.35745000	-1.35237200	0.41358300
H	-2.36893700	-0.88597700	2.12222800
C	-0.10292900	-2.26275000	2.92497800
H	-0.74880000	-2.00091800	3.76787500
H	0.45277400	-3.16807700	3.18415000
H	0.61594700	-1.44865500	2.80373400

Complex D

Mg	2.96697000	1.09760200	3.32765000
Cl	4.41296300	-0.26601100	1.92334100
C	2.86492300	0.31756500	5.30907300
H	3.82807000	-0.22616000	5.36877900
N	1.81216900	2.30843700	1.97787200
Li	5.71935600	1.54298100	1.53386300
C	0.63102900	2.93585300	2.63704900
C	1.48996200	1.55487900	0.72816400
H	-0.15026500	3.11196900	1.88593700
H	2.44176400	1.12139200	0.40138800

C	0.01633400	2.07707600	3.75341500
C	1.02221700	4.30369900	3.21374100
C	0.52053100	0.38213500	0.92844900
C	1.00223300	2.46355500	-0.41273500
H	0.87091000	-0.29421700	1.71563100
H	-0.48453100	0.72449000	1.19426900
H	0.43831600	-0.20159300	0.00620200
H	0.04751900	2.94504700	-0.17768800
H	1.73456100	3.24814300	-0.63812300
H	0.86201300	1.88628400	-1.33193800
H	0.19489500	4.75855900	3.76691000
H	1.85970600	4.20335100	3.91662800
H	1.30632300	5.01566700	2.42781300
H	-0.23874200	1.07643400	3.40111400
H	0.71419700	1.96806100	4.58991300
H	-0.89679900	2.54119100	4.14265200
Mg	3.29803400	3.67560800	1.57070500
Cl	4.73307600	2.93029100	-0.20736500
Cl	4.70621000	2.99823500	3.39395200
Mg	9.10382200	6.18805200	1.17596800
Cl	8.19857800	5.39727700	-0.94203800
C	8.89499500	8.30332300	1.35684300
H	8.02354400	8.49075900	0.69945900
N	10.36320000	4.60062800	1.89943900
Li	6.79236400	4.01752000	0.16722900
C	11.27743400	5.04169700	2.99148200
C	11.04770500	3.89985200	0.77139300
H	12.17324600	4.40685800	2.99054600
H	10.25734200	3.68811900	0.04275700
C	11.74237100	6.50048400	2.85988500
C	10.59192700	4.85615400	4.35279100
C	12.09761200	4.74818400	0.04066400
C	11.64573000	2.54342100	1.18143700
H	11.67485300	5.70260000	-0.29103300
H	12.96573600	4.95906300	0.67316000
H	12.45629200	4.22045600	-0.84860500
H	12.45674500	2.65250400	1.90878600
H	10.88309700	1.88583900	1.61628700
H	12.05370100	2.02250600	0.30958400
H	11.21533600	5.22963800	5.17083800
H	9.64663000	5.41324700	4.39090300
H	10.38659500	3.79970400	4.57003000
H	12.19680200	6.69603300	1.88757100
H	10.90005400	7.18961100	2.97852600
H	12.48014600	6.74381600	3.63267200
Mg	8.83831600	3.40493500	2.59681800
Cl	7.93807900	1.99833700	0.86758300
Cl	7.16903400	5.12907100	2.49728000
C	10.04819300	9.14269900	0.78942800
H	9.84723600	10.22827400	0.81899900
H	10.97605600	8.99110200	1.35801300
H	10.26915700	8.88760700	-0.25503500
C	8.51646900	8.80055900	2.75925400
H	9.33497400	8.64907600	3.47756500
H	8.28794400	9.88089800	2.78860100
H	7.64562100	8.27292000	3.16926100
C	1.76672500	-0.72016200	5.57763600
H	1.82691000	-1.16128400	6.58831300
H	0.76460900	-0.27610300	5.50030500
H	1.80251400	-1.55279800	4.86336700
C	2.87571600	1.37793200	6.41955400
H	1.94234600	1.95888200	6.43287000
H	2.97366300	0.94544000	7.43131300
H	3.68844500	2.10540400	6.29653900
S	4.70558500	6.19687000	0.53333900
O	3.41692900	5.62225800	1.22266200
C	4.13407200	6.91136300	-1.00930100
C	5.07927100	7.10015700	-2.01834500
C	2.78893400	7.23888000	-1.17161200
C	4.65397100	7.65977400	-3.22356300
H	6.11477600	6.80746600	-1.86857300
C	2.38171400	7.78668500	-2.38648600
H	2.08627900	7.05174900	-0.36714100
C	3.31271300	7.99990000	-3.40638400
H	5.37171200	7.81603800	-4.02188400
H	1.33861900	8.04485000	-2.53791300
H	2.98834300	8.42524800	-4.35073000
C	5.03969100	7.69817600	1.48683600
H	4.14720000	8.32485600	1.47959300
H	5.89559300	8.20064600	1.03076300
H	5.30111100	7.37247700	2.49341200
S	7.37365100	1.12701800	4.04213300
O	8.54155600	2.17382000	4.12146600
C	8.17204600	-0.47711600	4.10403400
C	9.46274400	-0.59803700	4.61666800

C	7.45776900	-1.56709000	3.60468800
C	10.05093100	-1.86102500	4.64050000
H	9.98889000	0.28210400	4.96959400
C	8.05972300	-2.82501600	3.64718200
H	6.46426100	-1.43333400	3.18537400
C	9.34912600	-2.97029400	4.16131100
H	11.05687900	-1.97924900	5.03000300
H	7.52272500	-3.68671000	3.26510900
H	9.81425300	-3.95070400	4.18109500
C	6.65146400	1.20411700	5.69947900
H	7.43933100	1.03404800	6.43418600
H	5.86685700	0.44672200	5.75996200
H	6.21100100	2.19623100	5.79691900 v

Int1-D

Mg	3.27945600	1.33159000	4.04575900
Cl	4.41580400	-0.34449400	2.69139900
C	2.15176800	0.80225200	5.75670000
H	2.87925100	0.45135700	6.50973400
N	3.10002500	5.63444100	1.64804500
Li	5.60506700	1.00820800	1.33634300
C	2.52812300	6.47665000	2.70398100
C	3.50122000	6.34735600	0.42367000
H	2.15691200	7.41756200	2.27196900
H	3.99698600	5.59865500	-0.21317200
C	1.32198300	5.77600200	3.34895600
C	3.54585000	6.85049400	3.79969800
C	2.29354400	6.86565400	-0.38399600
C	4.51438300	7.48933200	0.62531700
H	1.60632900	6.05188300	-0.63519700
H	1.72951200	7.61642800	0.18005700
H	2.61779100	7.33561700	-1.31903600
H	4.07865500	8.31384300	1.19974800
H	5.40240300	7.14028900	1.15488100
H	4.83642100	7.89747600	-0.33906400
H	3.10885900	7.52033000	4.55084600
H	3.88907600	5.94889900	4.32395700
H	4.42279100	7.33952200	3.37151900
H	0.53294800	5.60612100	2.60884500
H	1.62360000	4.80387800	3.75892000
H	0.90161000	6.36438600	4.17172600
Mg	4.11682400	4.01241900	2.14840400
Cl	4.59214600	2.68320300	0.07107600
Cl	4.94829700	3.10367100	4.26223500
Mg	8.92811800	5.86045200	0.66714600
Cl	8.33624400	4.65792400	-1.33461100
C	8.52278300	7.93972800	0.84042400
H	7.62397600	8.02896600	0.20705200
N	10.05170000	4.42727300	1.79713600
Li	6.77354700	3.49034000	-0.22068000
C	10.78853200	5.07412300	2.92161700
C	10.87896600	3.53790300	0.92396500
H	11.58128100	4.39805700	3.27051400
H	10.18455100	3.17149400	0.16027100
C	11.44483000	6.41571600	2.55503800
C	9.82814700	5.29959200	4.09887500
C	12.01522500	4.23755800	0.16521100
C	11.42364900	2.30086600	1.66039400
H	11.65593200	5.13027100	-0.35706700
H	12.83281500	4.53275500	0.82953900
H	12.43047400	3.56110700	-0.58865300
H	12.14802200	2.56997600	2.43592400
H	10.62227100	1.72014300	2.13203900
H	11.92784400	1.63042900	0.95739100
H	10.32388900	5.83193100	4.91598400
H	8.97039500	5.90999300	3.79328400
H	9.45983200	4.35614000	4.52251100
H	12.13213600	6.32149700	1.71461200
H	10.68458400	7.15503700	2.28728700
H	12.00820600	6.81152000	3.40721700
Mg	8.49598900	3.28244200	2.48355900
Cl	7.82150000	1.54055400	0.91493400
Cl	6.70439800	4.79018000	1.89451100
C	9.57404100	8.89554300	0.25826400
H	9.25461700	9.95169100	0.29605200
H	10.52466300	8.84820000	0.80869700
H	9.80238400	8.66869700	-0.79100600
C	8.11298100	8.39023100	2.25092000
H	8.94785100	8.32452500	2.96375400
H	7.77378900	9.44022500	2.27861800
H	7.29788600	7.78036900	2.66085000
C	1.14832900	-0.33830300	5.53341300
H	0.59249200	-0.60147500	6.45092600

H	0.39247900	-0.07111100	4.78535900
H	1.63693700	-1.25506600	5.17997300
C	1.44742800	2.03053400	6.35472400
H	0.71826700	2.45970700	5.65383300
H	0.88387000	1.79321500	7.27425600
H	2.15349000	2.83145500	6.60768400
S	1.68467400	1.96196700	1.33290500
O	2.61381900	2.52104500	2.50788200
C	0.18494900	1.50068500	2.18809800
C	-0.30619800	0.21601600	1.95127700
C	-0.44745700	2.38175100	3.07052500
C	-1.47805000	-0.18550900	2.59352400
H	0.22227800	-0.45770200	1.28454300
C	-1.60978800	1.96274000	3.70967700
H	-0.03063000	3.36412200	3.25881200
C	-2.12698100	0.68641400	3.46678000
H	-1.87212900	-1.18117800	2.42097400
H	-2.10813500	2.62946600	4.40517600
H	-3.03258500	0.36795600	3.97250400
C	1.18269300	3.46932500	0.45334100
H	1.75971700	4.32023100	0.90394300
H	0.10781800	3.60942700	0.55514800
H	1.46861600	3.33604900	-0.59019100
S	7.44234700	0.97490200	4.18294900
O	8.41817500	2.20168900	4.12984500
C	8.49639900	-0.46217000	3.95808700
C	9.86897800	-0.36245500	4.17661100
C	7.89474000	-1.64113900	3.51686200
C	10.66142900	-1.48753000	3.95619000
H	10.29839500	0.58166400	4.49245600
C	8.70139600	-2.76057300	3.31196800
H	6.82554200	-1.67695800	3.32955900
C	10.07831900	-2.68267800	3.52853100
H	11.73350500	-1.42959700	4.11349400
H	8.25413300	-3.68781500	2.96950800
H	10.70141500	-3.55405300	3.35475000
C	7.09022100	0.83339500	5.95448400
H	8.03569100	0.80488500	6.49782000
H	6.51571900	-0.08248600	6.10805000
H	6.49640100	1.70686400	6.22393700

TS1-D

Mg	3.27122000	1.30306500	3.87554300
Cl	4.43313100	-0.36078500	2.52048300
C	2.33317500	0.59872600	5.63644100
H	3.15284800	0.16033700	6.23513800
N	3.03792600	5.56406800	1.44583200
Li	5.62105900	0.96364700	1.16418000
C	2.65891300	6.47706800	2.54726900
C	3.34798100	6.22481800	0.15182300
H	2.24605300	7.40497700	2.13019100
H	3.63321200	5.40547700	-0.52235700
C	1.56241000	5.83644100	3.40947700
C	3.85308400	6.85956200	3.43885600
C	2.11645000	6.90618300	-0.47357700
C	4.52035600	7.21494200	0.18620400
H	1.27075200	6.21816400	-0.55041700
H	1.79411300	7.77354700	0.11137400
H	2.35403400	7.26535200	-1.48000700
H	4.27379200	8.10551300	0.77283600
H	5.41934500	6.76908000	0.61170600
H	4.75993300	7.54926800	-0.82859400
H	3.55531600	7.59223400	4.19762600
H	4.23754800	5.98066800	3.97165500
H	4.67335400	7.28393900	2.85892700
H	0.65296900	5.66091400	2.82871500
H	1.90162500	4.87525900	3.81412400
H	1.30865200	6.47980900	4.25740400
Mg	4.14884600	3.94895400	2.01436800
Cl	4.63951600	2.67832100	-0.06670500
Cl	4.93751300	3.10393300	4.16865300
Mg	8.92562200	5.85843900	0.68902000
Cl	8.44116600	4.63926400	-1.32916300
C	8.44472500	7.92517600	0.81202800
H	7.60444600	7.98520200	0.09900600
N	10.00766100	4.45758700	1.88870200
Li	6.83788000	3.45652100	-0.29090900
C	10.66355600	5.11952400	3.05449000
C	10.90205300	3.58647900	1.06448800
H	11.44782200	4.45974200	3.45043000
H	10.26067400	3.20853200	0.26062500
C	11.31319300	6.47541700	2.72879500
C	9.63028600	5.32745800	4.17236100
C	12.06659200	4.31060000	0.37513200
C	11.42629900	2.35779600	1.82917900
H	11.71972600	5.19641400	-0.16713300
H	12.83590000	4.62223300	1.08774500
H	12.54065800	3.64451200	-0.35269000
H	12.10073900	2.63740900	2.64475900
H	10.61021800	1.76198200	2.25461400
H	11.98053600	1.69756400	1.15484600
H	10.06856700	5.86571500	5.01781700
H	8.78332300	5.92486400	3.81552900
H	9.25084200	4.37859600	4.57384800
H	12.04765500	6.40106900	1.92689800
H	10.55513500	7.20341300	2.42520400
H	11.82189000	6.87645600	3.61238500
Mg	8.45455000	3.26369700	2.49925900
Cl	7.85641100	1.53943100	0.88694700
Cl	6.67695500	4.77369700	1.83128800
C	9.51287500	8.91325100	0.32192000
H	9.15840100	9.95881200	0.32550500
H	10.41104800	8.89685200	0.95565300
H	9.84178900	8.69165300	-0.70142600
C	7.89637400	8.36579100	2.17751100
H	8.66767400	8.33194800	2.96034700
H	7.51979000	9.40348500	2.16945800
H	7.07176600	7.72793400	2.51990600
C	1.31298100	-0.52256700	5.38748000
H	0.87753800	-0.91820600	6.32253500
H	0.46885900	-0.17270300	4.78113600
H	1.75736000	-1.37322400	4.85543800
C	1.71127700	1.71598400	6.48728300
H	0.89145100	2.21927000	5.95687700
H	1.27893300	1.34304900	7.43276800
H	2.44192700	2.49123600	6.75092800
S	1.55427300	2.20956600	1.25938700
O	2.63065400	2.57309100	2.42098700
C	0.15882200	1.67425300	2.24318600
C	-0.45067300	0.46855000	1.89365200
C	-0.30282100	2.44337400	3.31539500
C	-1.56250000	0.03784900	2.61881100
H	-0.05925700	-0.12359800	1.07274800
C	-1.40847700	1.99826000	4.03285000
H	0.20241900	3.36244500	3.58672600
C	-2.04119300	0.80190700	3.68231300
H	-2.04366400	-0.89895800	2.35847300
H	-1.77049200	2.58001600	4.87387400
H	-2.90020200	0.45965700	4.24997400
C	1.09045600	3.79602100	0.64088700
H	1.98000200	4.68082000	1.06474100
H	0.10320100	4.07563300	1.00589600
H	1.11190400	3.73591000	-0.44812100
S	7.37536900	0.94060200	4.15511300
O	8.35843300	2.16463400	4.12964600
C	8.43148700	-0.49924300	3.96052200
C	9.79656100	-0.40696300	4.22549300
C	7.83905700	-1.67458500	3.49759500
C	10.59010300	-1.53596500	4.03094900
H	10.21996200	0.53448400	4.55700700
C	8.64653700	-2.79802600	3.31899100
H	6.77673900	-1.70393300	3.27368300
C	10.01557300	-2.72775000	3.58252700
H	11.65645500	-1.48405400	4.22516900
H	8.20615100	-3.72255200	2.96063300
H	10.63959200	-3.60239500	3.42963000
C	6.97117800	0.79879100	5.91556500
H	7.89973500	0.75514200	6.48630700
H	6.37810100	-0.10843900	6.04837900
H	6.38065500	1.67879800	6.17010600

Int2-D

Mg	3.02458300	2.38597600	4.65055400
Cl	3.78503500	0.07973600	4.54985700
C	1.46233000	2.80652300	6.03713700
H	2.03443800	3.12313900	6.92738100
N	2.87664800	5.85223900	1.38633800
Li	4.73138500	1.20390100	2.80236800
C	1.98870000	6.36193800	2.48524800
C	3.55407600	6.91455800	0.54233300
H	1.68581000	7.38620300	2.23988300
H	4.30671000	6.37189700	-0.03695600
C	0.73346300	5.49551300	2.55167700
C	2.73574500	6.36812300	3.81801200
C	2.55427500	7.53219100	-0.43836700
C	4.26699400	7.98198900	1.36845800

H	2.10338500	6.76654200	-1.07910500
H	1.75175000	8.06557000	0.08244600
H	3.06182400	8.25015500	-1.08824000
H	3.56605700	8.60218800	1.93493200
H	4.99742300	7.55026100	2.05173200
H	4.80954200	8.64111400	0.68577200
H	2.11237500	6.80952200	4.60006300
H	2.97756100	5.34990200	4.14145300
H	3.66753600	6.93183700	3.76559500
H	0.18687800	5.51185700	1.60212500
H	0.99127500	4.46104300	2.79367600
H	0.06418700	5.85018600	3.33783200
Mg	4.16874400	4.20190400	1.84495000
Cl	4.69193100	3.63161600	-0.41126200
Cl	5.16826600	3.55495400	5.09795400
Mg	8.87272500	5.67609500	2.06194300
Cl	8.92176500	4.23864700	0.10821100
C	9.03752600	7.76233500	1.67417100
H	8.50161800	7.84590900	0.71190600
N	9.17842200	4.46367500	3.77848500
Li	6.85323900	3.51097400	0.45293900
C	9.35210900	5.28665100	5.01053900
C	10.31718700	3.53187500	3.47601100
H	9.54853900	4.62161900	5.86481800
H	9.88110700	2.74326000	2.84653000
C	10.50192800	6.30637400	4.95615000
C	8.04758400	6.03144000	5.32110900
C	11.44929500	4.15069900	2.63002900
C	10.93412400	2.84493700	4.70483400
H	11.08100000	4.58061400	1.69419100
H	11.99977300	4.92109700	3.17250900
H	12.16380400	3.37208500	2.34059100
H	11.51971900	3.54437100	5.30918000
H	10.16713100	2.39918400	5.33935200
H	11.62027200	2.05675100	4.37500800
H	8.15493600	6.60724700	6.24483800
H	7.78358400	6.73147700	4.52382600
H	7.19376700	5.36112700	5.47614500
H	11.47969600	5.82424300	4.91341300
H	10.40233000	6.95799300	4.08233700
H	10.48920000	6.93904300	5.85031300
Mg	7.39042600	3.39539400	3.98700200
Cl	6.84087900	1.82983700	2.15861300
Cl	6.26393000	5.24190400	2.35764100
C	10.47875200	8.22945000	1.41739600
H	10.53824200	9.28682300	1.10370000
H	11.10303600	8.14666000	2.31916100
H	10.97121100	7.63755400	0.63507500
C	8.34001400	8.71439500	2.65468700
H	8.81439600	8.70058100	3.64584600
H	8.36605500	9.76728300	2.32148500
H	7.28356400	8.45498200	2.80758400
C	0.74732200	1.49804400	6.41469700
H	0.04871100	1.62442200	7.26016100
H	0.14415200	1.11191100	5.58009500
H	1.44904100	0.70145500	6.68924600
C	0.42362100	3.90044600	5.76851600
H	-0.17939900	3.67862700	4.87654100
H	-0.29480400	4.01544800	6.59909100
H	0.88241600	4.88311000	5.60831200
S	1.84620200	1.92830700	1.66753600
O	3.37943000	2.60755800	2.63708800
C	2.62815100	0.34406100	1.29288700
C	3.88459900	0.37101600	0.67623400
C	2.01035000	-0.86686600	1.60114200
C	4.55741300	-0.82801400	0.43851900
H	4.33199000	1.31301300	0.37840200
C	2.69074400	-2.05848200	1.35198000
H	1.01918300	-0.88582600	2.03449200
C	3.96611500	-2.04302300	0.78716900
H	5.54131000	-0.80055300	-0.01724200
H	2.21870200	-3.00119000	1.60841200
H	4.49067900	-2.97511700	0.60375400
C	0.58418500	1.56174300	2.62497800
H	2.26393000	5.37372600	0.72301600
H	0.61776700	0.75168400	3.34296100
H	-0.14488200	2.34973900	2.75934100
S	7.14846900	0.70864100	5.66778400
O	7.96024900	1.98758400	5.30838200
C	7.69626800	-0.53602700	4.48330100
C	8.94588500	-0.40123800	3.87904400
C	6.81535700	-1.56091000	4.14184900
C	9.32512200	-1.32937800	2.91355400
H	9.58611100	0.43406400	4.13696800
C	7.21246700	-2.48669900	3.17539000

H	5.83039800	-1.61233800	4.59139400
C	8.46015000	-2.36964200	2.56221600
H	10.28902500	-1.23318400	2.42469900
H	6.53643200	-3.28768000	2.89450400
H	8.75859000	-3.08472800	1.80202200
C	8.03818900	0.10719600	7.13482800
H	9.10206300	0.05117500	6.89959300
H	7.64088100	-0.87470400	7.39965300
H	7.85388600	0.82306600	7.93738300

TS2-D

Mg	3.15678600	2.40073800	4.51518600
Cl	3.85159900	0.06660900	4.38775000
C	1.56308900	2.75858600	5.94692900
H	2.30594800	3.07220600	6.70469300
N	2.89798700	5.87617800	1.24277100
Li	4.68910800	1.24490100	2.57421700
C	2.06463000	6.38219000	2.38517800
C	3.55498400	6.93074500	0.37626600
H	1.73501100	7.40184200	2.15334000
H	4.26620000	6.37505400	-0.24258700
C	0.82784100	5.49583700	2.52065100
C	2.87472500	6.40192900	3.68155000
C	2.52401100	7.58288100	-0.54863000
C	4.33275100	7.97197600	1.17660100
H	2.01689800	6.83522600	-1.16855900
H	1.76599600	8.13641100	0.01577800
H	3.01768900	8.28967600	-1.22109300
H	3.67378600	8.60009100	1.78353500
H	5.08418700	7.51518100	1.81969600
H	4.85643700	8.62762400	0.47608600
H	2.27318000	6.81821000	4.49429600
H	3.16714600	5.39018900	3.98254800
H	3.78452300	6.99536600	3.59319700
H	0.23373900	5.49222100	1.59988200
H	1.12141600	4.46894600	2.75553700
H	0.19116200	5.84693000	3.33524000
Mg	4.17575600	4.21121000	1.69513500
Cl	4.81909100	3.63504900	-0.52502600
Cl	5.18485500	3.54865500	5.28564100
Mg	8.82178400	5.67869400	2.13036100
Cl	8.94709700	4.27515700	0.15331800
C	8.97517900	7.76636600	1.73670100
H	8.43820000	7.83982100	0.77397200
N	9.12679500	4.46803400	3.85014000
Li	6.92132200	3.43415900	0.46978700
C	9.28768900	5.30538300	5.07439500
C	10.27489100	3.54091900	3.56777700
H	9.46878400	4.64994300	5.93976100
H	9.85009800	2.74168500	2.94402400
C	10.44424200	6.31831900	5.02370900
C	7.98534300	6.06419800	5.35881600
C	11.41130900	4.15647700	2.72492600
C	10.88637100	2.87161300	4.80934600
H	11.04804300	4.57730500	1.78346300
H	11.95634500	4.93250100	3.26484700
H	12.13003700	3.37707200	2.44766500
H	11.46064000	3.58195100	5.41171600
H	10.11792800	2.42407000	5.44041600
H	11.58250200	2.08646300	4.49303500
H	8.08686900	6.64992100	6.27715600
H	7.73924200	6.75854500	4.55084100
H	7.12504500	5.40281300	5.50748300
H	11.42046600	5.83197300	5.00019800
H	10.35946700	6.96027200	4.14102200
H	10.42169700	6.96184800	5.90996500
Mg	7.33900400	3.37680600	4.03689700
Cl	6.87932600	1.79111300	2.17705800
Cl	6.23617800	5.20008000	2.40757100
C	10.41438400	8.23604800	1.47391700
H	10.47102500	9.29089300	1.15056000
H	11.03979100	8.16298900	2.37584300
H	10.90750100	7.63822000	0.69652400
C	8.27594100	8.72521800	2.70877200
H	8.75405000	8.72419000	3.69820500
H	8.29463100	9.77494000	2.36459700
H	7.22148900	8.46150400	2.86918100
C	0.86843400	1.51025000	6.49517200
H	0.37893900	1.69538900	7.46641300
H	0.07330500	1.15520200	5.82487400
H	1.56430800	0.67492200	6.63446700
C	0.57704200	3.91408200	5.78711800
H	-0.17068200	3.71917400	5.00393900

H	0.00054900	4.10598000	6.70858700
H	1.07631700	4.85266900	5.52292200
S	1.54363700	1.82371400	1.83075000
O	3.47763600	2.68578300	2.58128100
C	2.35716500	0.32311400	1.28831800
C	3.46441900	0.47213200	0.44293000
C	1.91252900	−0.94203200	1.67444700
C	4.16681200	−0.66243800	0.03653300
H	3.79090900	1.45509000	0.11905900
C	2.62215400	−2.06609900	1.25550800
H	1.03536900	−1.05497600	2.29880000
C	3.75386800	−1.93040400	0.45077900
H	5.03700700	−0.54465200	−0.60038800
H	2.28793400	−3.05027700	1.56662400
H	4.30503700	−2.81056600	0.13627500
C	0.70435500	1.42582500	3.16844500
H	2.25186200	5.40728100	0.60507500
H	0.92978900	0.54576400	3.75571000
H	−0.00957200	2.15043800	3.53376200
S	7.12684800	0.65622100	5.64685200
O	7.94036600	1.94037800	5.31666000
C	7.74510000	−0.59014200	4.50046600
C	9.01759000	−0.44115700	3.95054800
C	6.90017700	−1.63827700	4.14029000
C	9.46004100	−1.38194100	3.02476600
H	9.62700400	0.41349900	4.21986500
C	7.36139900	−2.57797000	3.21690800
H	5.89508200	−1.69618400	4.54251200
C	8.63432600	−2.44883100	2.66007300
H	10.44317500	−1.27579500	2.57795600
H	6.71681900	−3.40024100	2.92315900
H	8.98210300	−3.17509200	1.93211600
C	7.95732200	0.07327700	7.15622200
H	9.03136400	0.03024200	6.96932300
H	7.56214900	−0.91251900	7.40953400
H	7.72805900	0.79160100	7.94488100

Prod-D

Mg	3.86505100	2.08854900	3.22523700
Cl	4.39934800	−0.22348600	2.93032800
C	2.74295600	0.49496000	6.13048400
H	3.68735900	0.74361100	5.63115300
N	3.71336500	5.64959600	−0.22281600
Li	5.29348900	1.25704200	1.17088800
C	2.52739600	6.06940400	0.59885500
C	4.52355200	6.74677200	−0.87107800
H	2.19193600	7.05479700	0.25392000
H	5.39876300	6.23047700	−1.28124500
C	1.40414000	5.05650400	0.37612600
C	2.88853400	6.15477500	2.08321100
C	3.75611500	7.38233700	−2.03326800
C	5.01385400	7.79446600	0.12517700
H	3.44952600	6.62850100	−2.76672200
H	2.86205600	7.91141200	−1.68752300
H	4.39097000	8.10809500	−2.54891200
H	4.18740000	8.37599800	0.54495300
H	5.58511800	7.35015000	0.94053800
H	5.67584000	8.49220800	−0.39409500
H	2.03128300	6.52618200	2.65249700
H	3.13938500	5.16335600	2.47695200
H	3.73424400	6.81619400	2.27057700
H	1.08754300	5.03021300	−0.67256900
H	1.73408100	4.05474900	0.66748000
H	0.53179500	5.31299300	0.98302300
Mg	4.88083200	4.10376500	0.67842200
Cl	6.21718700	3.35194500	−1.13788700
Cl	5.01083700	3.33828600	4.86143400
Mg	8.93410300	5.98598700	2.43902600
Cl	9.74061500	4.69893200	0.55176700
C	8.84575300	8.08476700	2.08769300
H	8.49002000	8.09697500	1.04097700
N	9.04568700	4.75266300	4.17160400
Li	7.97191100	3.38492000	0.35408300
C	8.84615300	5.56116300	5.40886800
C	10.32003000	3.95860500	4.13740200
H	8.92238700	4.90287800	6.28755700
H	10.13644900	3.15455200	3.41053900
C	9.85761000	6.70305800	5.60882700
C	7.43131500	6.15217300	5.42077200
C	11.55089900	4.71962200	3.60333800
C	10.69173500	3.28578800	5.46871300
H	11.36292700	5.18263700	2.63130100
H	11.89460700	5.49106200	4.29448900
H	12.37895400	4.01841700	3.45048800
H	11.04027200	4.01443500	6.20725200
H	9.84637400	2.74153100	5.89242500
H	11.51355900	2.58061200	5.30203800
H	7.26926200	6.71803000	6.34299700
H	7.27232100	6.83609600	4.58294400
H	6.64920200	5.38726100	5.38246400
H	10.86666500	6.33410100	5.79700700
H	9.88875500	7.35390000	4.72849400
H	9.56903300	7.31697800	6.46904200
Mg	7.42491500	3.45032200	3.91171000
Cl	7.50590400	1.81578200	2.03665100
Cl	6.46169400	5.15839700	2.17122200
C	10.21692300	8.77738600	2.09533100
H	10.16889800	9.83439000	1.77715500
H	10.66374400	8.78519000	3.10049000
H	10.93503500	8.27761600	1.43257800
C	7.83854800	8.90128800	2.90707500
H	8.12125300	8.94965900	3.96801100
H	7.75716200	9.94962700	2.56747100
H	6.82644900	8.47532400	2.87055700
C	2.91455000	−0.89583900	6.75421300
H	3.70363700	−0.87888300	7.51318800
H	1.99234700	−1.22684200	7.24528700
H	3.18474400	−1.63623700	5.99548200
C	2.46251500	1.56323700	7.19112700
H	1.50646000	1.37423300	7.69287300
H	3.24789700	1.56109000	7.95311100
H	2.43026900	2.56743200	6.75982000
S	1.48404300	2.01899900	4.13094700
O	4.10446000	2.60504700	1.45235900
C	0.52348700	1.49890000	2.69991600
C	1.10342400	1.45874200	1.42719500
C	−0.82350700	1.16829700	2.88423000
C	0.31576400	1.07709300	0.33909800
H	2.14656000	1.73956900	1.27481500
C	−1.59160500	0.77352300	1.79067900
H	−1.26650000	1.22411000	3.87347300
C	−1.02292500	0.72891000	0.51624100
H	0.76091600	1.04780000	−0.65071300
H	−2.63508000	0.51075100	1.93346300
H	−1.62457700	0.42719200	−0.33544200
C	1.64765500	0.41908700	5.06093100
H	3.33871700	5.11683100	−1.01022000
H	1.88349700	−0.34520300	4.31903400
H	0.67504900	0.20636600	5.51355200
S	6.97440500	0.68956000	5.19359000
O	7.82257700	1.99405800	5.26146500
C	8.11188500	−0.58713500	4.64375700
C	9.48547700	−0.40926900	4.80034400
C	7.57288000	−1.71215600	4.02053500
C	10.34206100	−1.40590800	4.33765900
H	9.86606400	0.50193700	5.24704300
C	8.44445500	−2.70486400	3.57197400
H	6.50204700	−1.78988900	3.86268300
C	9.82267700	−2.55211200	3.73068600
H	11.41529400	−1.28377200	4.44241700
H	8.04586800	−3.58686200	3.08135200
H	10.49624500	−3.32169400	3.36715200
C	6.83181600	0.23141900	6.94553500
H	7.82600800	0.24577600	7.39499100
H	6.38406400	−0.76244900	7.00826300
H	6.18257600	0.97577000	7.40969600

Part 2: Experimental Details

Table of Contents

1. Experimental Procedures S2-3

1.1 Material and methods S2-3

1.2 Synthesis of starting materials 7a and 7a-*d₃* S2-3

Synthesis of *i*-$Pr_2NMgCl\cdot LiCl$ (**4a**) S2-3

Synthesis of methyl phenyl sulfoxide (**7a**) S2-4

Synthesis of thioanisole-d_3 (**S2**) S2-5

Synthesis of **7a-d_3** S2-6

1.3 NMR experiments S2-7

Signal stability on No-D ^{1}H-NMR experiments S2-7

General procedure for NMR experiments (GP 1) S2-8

Kinetic profiling of the conversion of **7a** to **8a** followed by ^{1}H-NMR spectroscopy S2-9

Kinetic profiling of the conversion of **7a-d_3** to **8a-d_2** by *in-situ* NMR experiments S2-12

3. ^{1}H-NMR and ^{13}C-NMR spectra of starting materials S2-16

^{1}H-NMR (400 MHz. $CDCl_3$) of **7a** S2-17

^{1}H-NMR (400 MHz. $CDCl_3$) of **S2** S2-18

^{13}C-NMR (100 MHz. $CDCl_3$) of **S2** S2-19

^{1}H-NMR (400 MHz. $CDCl_3$) of **7a-d_3** S2-20

^{13}C-NMR (100 MHz. $CDCl_3$) of **7a-d_3** S2-21

4. References S2-22

1. Experimental Procedures

1.1 Material and methods

Moisture sensitive reactions were carried out using oven dried glassware under an atmosphere of argon unless otherwise specified. Dry solvents were obtained by passing them through activated alumina columns. Solvents were acquired from commercial sources: tetrahydrofuran (THF, VWR 1146001 tetrahydrofuran AnalaR NORMAPUR® ACS, Reag. Ph. Eur.), dichloromethane (DCM, VWR 1108025 AnalaR NORMAPUR® ACS, Reag. Ph. Eur.) and ethanol (99.5%, KiiltoClean). The solvents used in column chromatography were obtained from commercial suppliers and used without further purification. Reagents, *i*-Propylmagnesium chloride (2.0 M in Et_2O, Sigma-Aldrich, 1068-55-9) iodomethane-D3 (99.5+ Atom% D, Aldrich, 212-744-5), thioanisole (99%, Lancaster, 100-68-5) and 2-chloropropan (99%, Sigma-Aldrich, 75-29-6) were obtained from commercially available sources and used as received unless otherwise noted. *i*-Propylmagnesium chloride lithium chloride complex (*i*-PrMgCl·LiCl) was prepared following the reported procedure by Knochel.[1] Grignard reagents were titrated with iodine using the method reported by Knochel.[2] Diisopropylamine (Sigma-Aldrich, 108-18-9) was distilled prior to use.

NMR spectra were recorded at room temperature on a Bruker instrument 400 MHz (^{1}H) and at 100 MHz (^{13}C) or 500 MHz (^{1}H) and at 125 MHz (^{13}C). Chemical shifts (δ) are reported in ppm, using the residual solvent peak in $CDCl_3$ (δ_H = 7.26 and δ_C = 77.16 ppm) as internal reference. Data are reported in chemical shift, multiplicity (s: singlet, d: doublet, sept: septet m: multiplet), coupling constants (*J*) in hertz (Hz) and integration. Kinetic measurements were carried out a Bruker instrument 500 MHz using no-D spectroscopy in THF, using the solvent signal (δ_H = 3.58 ppm) as chemical shift reference and starting material integration as concentration reference.

1.2 Synthesis of starting materials 7a and 7a-*d₃*

Synthesis of *i*-Pr_2NMgCl·LiCl (**4a**)

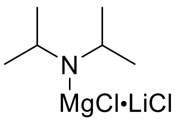

4a

According the reported procedure of Knochel and co-workers,[3] freshly distilled diisopropylamine (0.31 mL, 2.2 mmol, 1.1 equiv.) was dissolved with THF (0.2 mL) in an oven dried flask under inert atmosphere. *i*-PrMgCl·LiCl (2.4 mL of a 0.85 M solution in THF, 2.0 mmol, 1.0 equiv.) was added dropwise at r.t. and the mixture was stirred for 1 h. The

concentration of *i*-$Pr_2NMgCl\cdot LiCl$ (**4a**) in the solution was determined by double titration in water using phenolphthalein as indicator, yielding a concentration of 0.74-0.80 M.

Synthesis of methyl phenyl sulfoxide (**7a**)

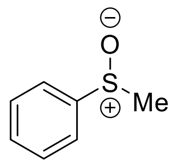

7a

Following a procedure from Kakarla and co-workers,[4] thioanisole (1.2 mL, 10.0 mmol, 1.0 equiv.), SiO_2 (2.0 g, 33.3 mmol, 3.33 equiv.) and acetic anhydride (1.0 mL, 11.0 mmol, 1.1 equiv.) were suspended in CH_2Cl_2 (50 mL) and hydrogen peroxide (30% in H_2O, 12.0 mmol, 1.2 equiv.) was added. The resulting mixture was stirred at r.t. for 36 h. The mixture was filtered, the filtrate diluted in water (20 mL) and the aqueous layer extracted with CH_2Cl_2 (3 x 15 mL). The combined organic layers were washed with brine, dried over Na_2SO_4 and concentrated in vacuo. The residue was purified by column chromatography on silica (eluent: Pentane/EtOAc = 1:3) to afford **7a** as a colorless oil (700 mg, 5.0 mmol, 50%).

Appearance: colorless oil

TLC: R_f = 0.21 (Pentane/EtOAc, 3:1, UV-active)

^{1}H-NMR (400 MHz, $CDCl_3$) δ (ppm) = 7.73 – 7.59 (m, 2H), 7.58 – 7.45 (m, 3H), 2.74 – 2.68 (s, 3H).

The spectroscopic data was found consistent with the literature.[5]

Synthesis of thioanisole-d_3 (**S2**)

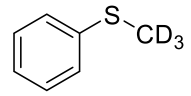

S2

Following a reported procedure,[6] a suspension of thiophenol (0.51 mL, 5.00 mmol, 1.0 equiv.) and K_2CO_3 (0.55 g, 5.19 mmol, 1.04 equiv.) was stirred in ethanol (25 mL) at r.t. for 1 h. Iodo($^2H_3$)methane (0.34 mL, 5.50 mmol, 1.10 equiv., 99% D) was added slowly, and the mixture was stirred at 30 °C for 36 h. The solvent was partially evaporated and H_2O (25 mL) was added to the mixture. The aqueous layer was extracted with CH_2Cl_2 (3 x 15 mL) and the org. layers combined, washed with water and brine and dried over Na_2SO_4. The solvent was removed, and the residue purified by column chromatography on silica (pentane/EtOAc = 9:1) to obtain **S2** as a colorless oil in 63% yield (>99% D determined by ^{1}H-NMR).

Appearance: colorless oil

TLC: R_f = 0.21 (pentane, UV-active)

^{1}H-NMR (400 MHz, $CDCl_3$) δ (ppm) = 7.31 – 7.19 (m, 4H), 7.14 – 7.05 (m, 1H).

^{13}C-NMR (101 MHz, $CDCl_3$) δ (ppm) = 138.5, 128.9, 126.8, 125.1, 15.3 (extremely weak *C*D_3).

The spectroscopic data was found consistent with the literature.[6]

Synthesis of **7a-*d*₃**

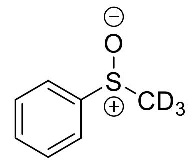

7a-*d*3

To a suspension of sulfide **S2** (400 mg, 3.14 mmol, 1.0 equiv.), silica gel (629.1 mg, 10.47 mmol, 3.33 equiv.) and acetic anhydride (0.33 mL, 3.46 mmol, 1.10 equiv.) in CH_2Cl_2 (20 mL) stirred at r.t., was added hydrogen peroxide (30% in H_2O, 0.29 mL, 3.77 mmol, 1.2 equiv.). The mixture was stirred at r.t. for 24 h, then filtered, diluted with water (10 mL) and the aq. layer extracted with CH_2Cl_2 (3x 5 mL). The org. layers were combined, dried over Na_2SO_4 and the solvent removed in vacuo. The residue was purified by column chromatography on silica (Pentane/EtOAc = 2:5) to afford **7a-*d*3** (308 mg, 2.2 mmol, 68%) as a colorless oil (>99% D determined by ^{1}H-NMR).

Appearance: colorless oil

TLC: R_f = 0.16 (Pentane/EtOAc, 5:2, UV-active)

^{1}H-NMR (400 MHz, $CDCl_3$) δ (ppm) = 7.75 – 7.61 (m, 2H), 7.56 – 7.45 (m, 3H).

^{13}C-NMR (101 MHz, $CDCl_3$) δ (ppm) = 145.6, 131.1, 129.4, 123.5, 43.22 (sept., *J* = 21.5 Hz).

The spectroscopic data was found consistent with the literature.[7]

1.3 NMR experiments

Signal stability on No-D ^{1}H-NMR experiments

To make sure that the reaction can be monitored without internal reference, the signal stability was assessed in No-D ^{1}H-NMR experiments over a long time period (8 h) using absolute integration numbers. Whenever possible, avoiding an internal standard on reactions with highly reactive intermediates removes potential interferences caused by degradation of the internal standard in solution, and improves the signal-to-noise ratio and shimming compared to NMR tube inserts.

A solution of 1,1,2,2-tetrachloroethane (TCE, 5.0 µL, 0.05 mmol) in THF (0.5 mL) was used to determine the signal stability of the NMR measurements under the experimental conditions. Consecutive No-D ^{1}H-NMR spectra were recorded over a time period of 8 h every 240 s. The absolute integral of the TCE in the first scan was assigned to its initial known concentration (0.1 M) and used to reference the integral values in the subsequent measurements (Figure S1). Expectedly, the signal oscillated stochastically over time around the expected value with a maximum relative error of 2.6% of the expected value (Table S1).

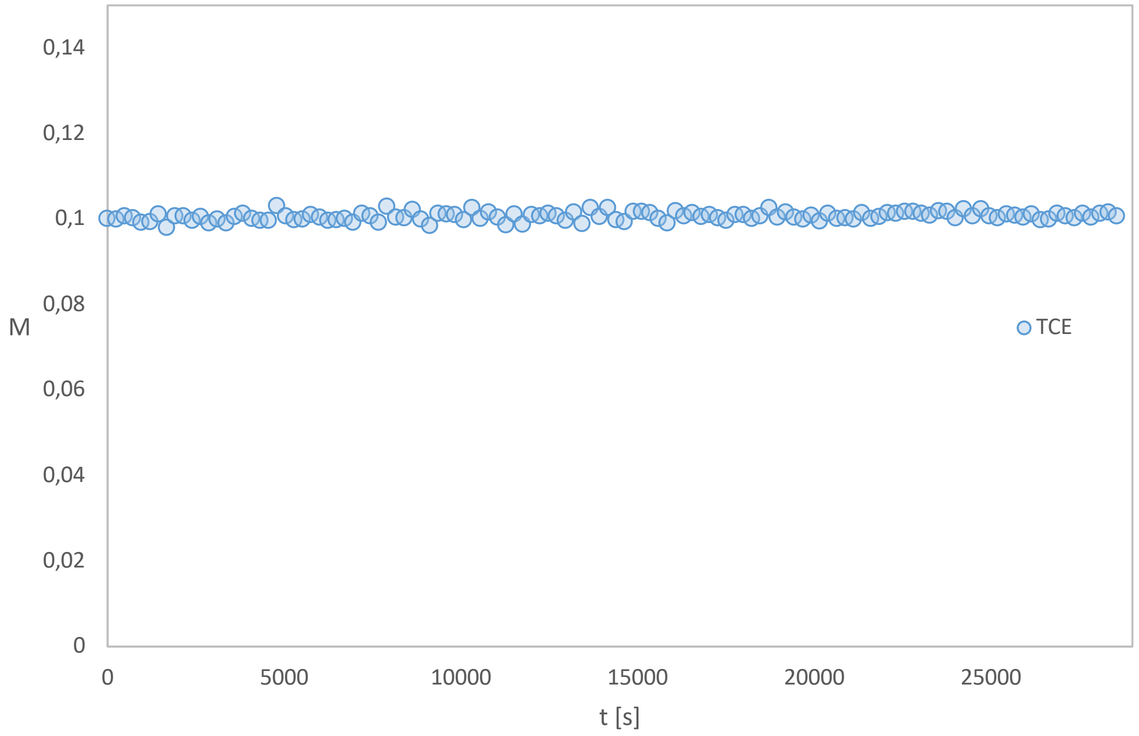

Figure S1: *Signal stability of No-D ^{1}H-NMR experiments monitoring TCE (0.1 M) in THF over 8 h.*

***Table S1**: Summary of the signal stability measurements.*

**Monitoring time (h)**	8
**Measurements**	120
$\overline{[TCE]}$ **(M)**	0.100(5)
$[TCE]_{Max}$ **(M)**	0.103(0)
$\Delta[TCE]_{Max}$ **(M)**	0.002(5)
$[TCE]_{Min}$ **(M)**	0.097(9)
$\Delta[TCE]_{Min}$ **(M)**	0.002(6)
**Maximum relative error (%)**	2.6 %

General procedure for NMR experiments (GP 1)

The procedure was adapted from the original report by Mendoza and co-workers.[8] NMR experiments were performed in oven-dried, Ar-flushed NMR tubes equipped with a septum. Solutions were transferred using gas tight syringes that were flushed with inert gas prior to use. The appropriate sulfoxide **7** (0.05 mmol) was weighed into the NMR tube, the atmosphere exchanged to Ar followed by the addition of dry THF. The mixture was cooled down to 0 °C with an ice bath and base **4a** (1.1 equiv.) was added. The solution was allowed to warm up to r.t. at which point the spectrometer was shimmed and tuned to the sample, *i*-PrMgCl (**1e**, 2.0 M in THF, 1.05 equiv.) was added in one shot. The evolution of the system was monitored with consecutive ^{1}H-NMR experiments at 25 °C.

Kinetic profiling of the conversion of **7a** to **8a** followed by ^{1}H-NMR spectroscopy

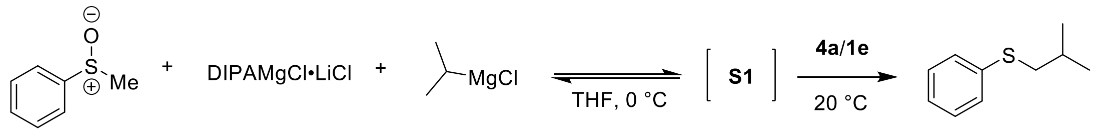

Following GP 1, a solution of sulfoxide **7a** (0.05 mmol) in THF (0.4 mL) was mixed with DIPAMgCl·LiCl (**4a**, 73 µL, 0.75 M in THF, 55 µmol, 1.1 equiv.) at 0 °C and a ^{1}H-NMR spectrum was recorded. Grignard reagent **1e** (26 µL, 2.0 M in THF, 53 µmol, 1.05 equiv.) was added at r.t. and the progress of the reaction was followed by ^{1}H-NMR spectroscopy.

[a]Note: The broad signals denoted as **S1** are likely an average of various starting material complexes in fast equilibrium in solution. The inherent stability of this mixture prevented further structural characterization by NMR.

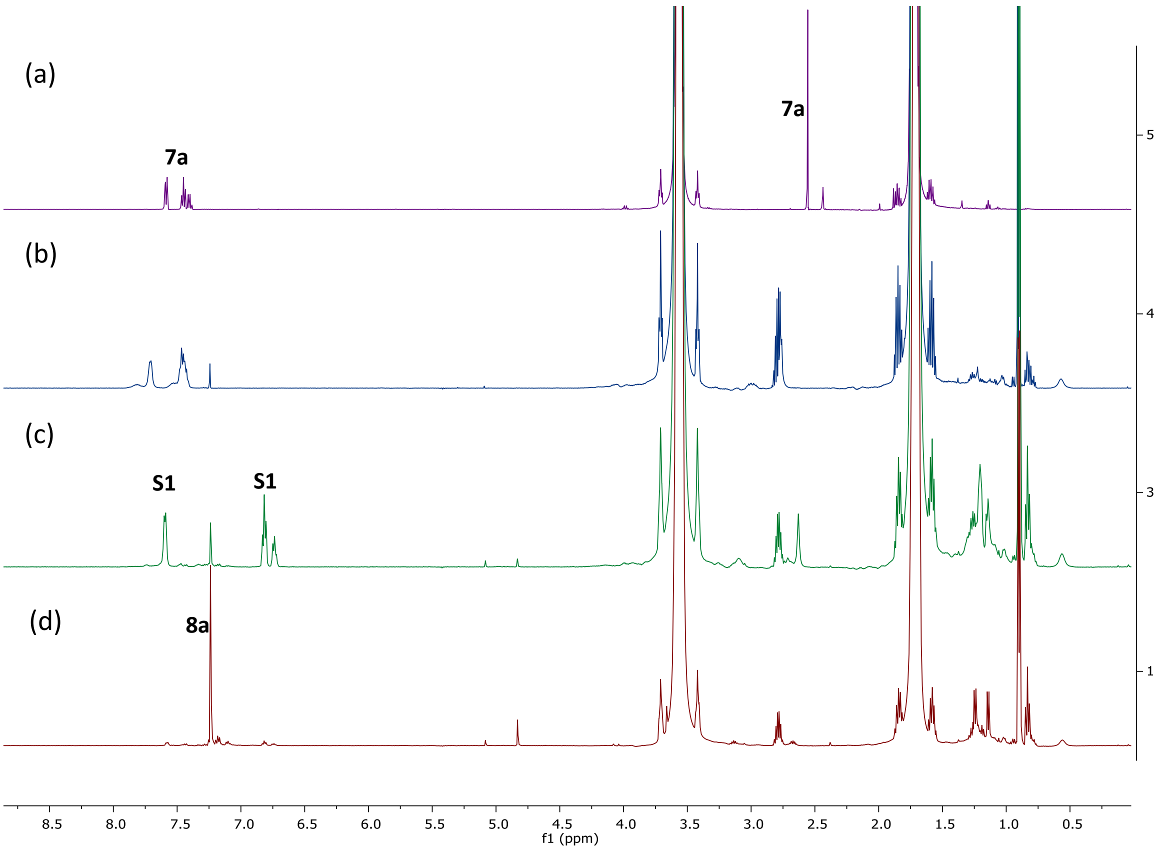

Figure S2: *^{1}H-spectra of sulfoxide **7a** a) in THF, b) after addition of turbo-Hauser base **4a**, c) after addition of Grignard reagent **1e**, d) after 3.4 h of reaction time.*

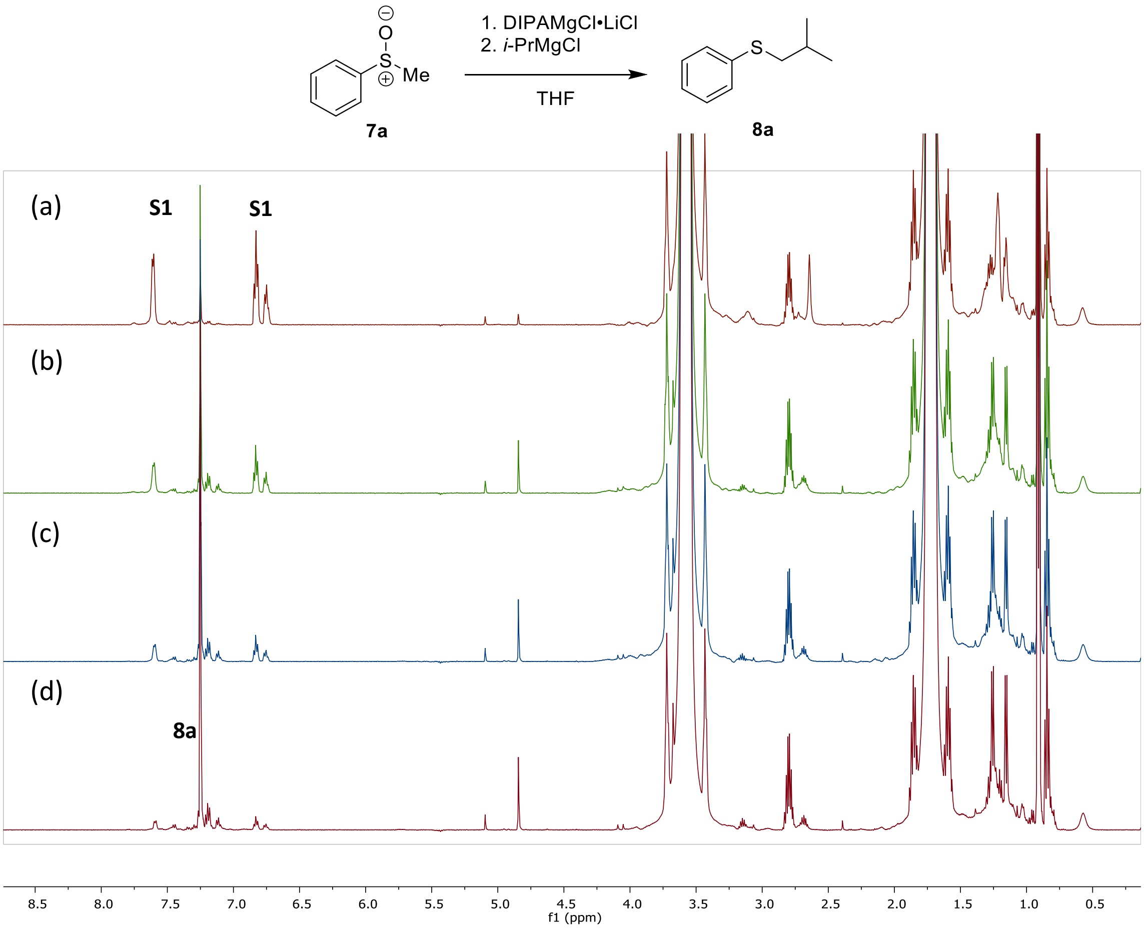

Figure S3: *^{1}H-NMR spectra of the conversion of* ***S1*** *to* ***8a*** *Reaction progress of the Pummerer reaction a) directly after the addition of i-PrMgCl (**1e**), b) after 54 min reaction time c) after 144 min reaction time d) after 3.4 h reaction time.*

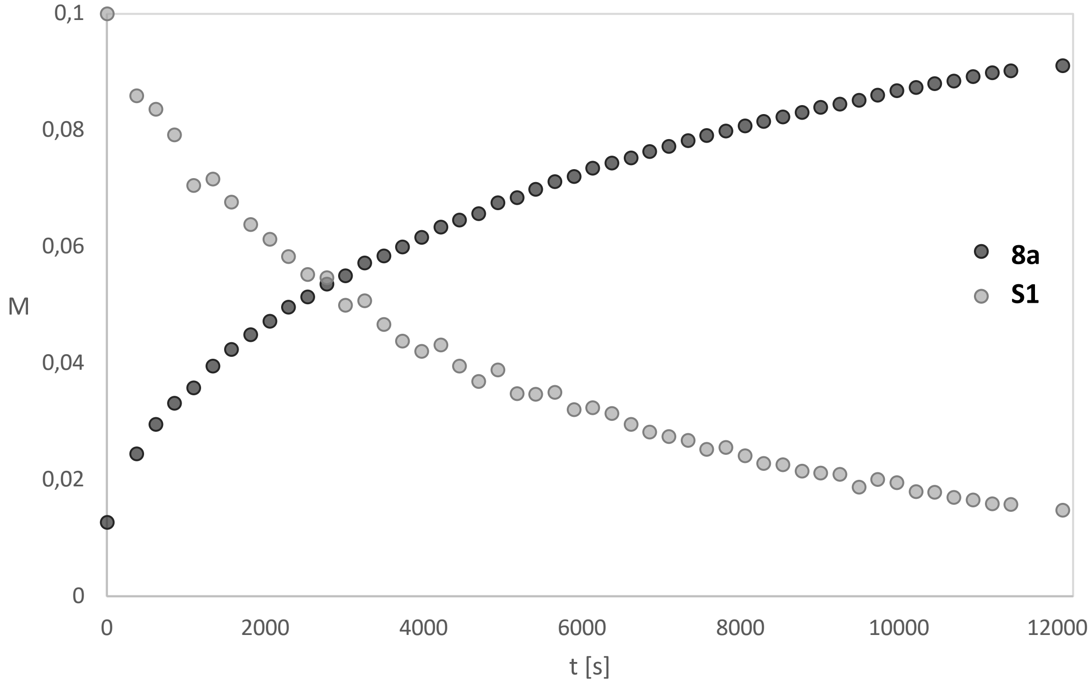

Figure S4: *Concentration-time profile for the consumption of intermediate* ***S1*** *and formation of product* ***8a****.*

***Table S2**. Data of the conversion of **S1** with time (see Figure S4)*

Entry	t (s)	[S1] (M)	[8a] (M)	Entry	t (s)	[S1] (M)	[8a] (M)
1	0	0.10	0.013	26	6135	0.032	0.073
2	375	0.086	0.024	27	6375	0.031	0.074
3	615	0.084	0.030	28	6615	0.030	0.075
4	855	0.079	0.033	29	6855	0.028	0.076
5	1095	0.071	0.036	30	7095	0.027	0.077
6	1335	0.072	0.039	31	7335	0.027	0.078
7	1575	0.068	0.042	32	7575	0.025	0.079
8	1815	0.064	0.045	33	7815	0.026	0.080
9	2055	0.061	0.047	34	8055	0.024	0.081
10	2295	0.058	0.050	35	8295	0.023	0.082
11	2535	0.055	0.051	36	8535	0.023	0.082
12	2775	0.055	0.054	37	8775	0.022	0.083
13	3015	0.050	0.055	38	9015	0.021	0.084
14	3255	0.051	0.057	39	9255	0.021	0.085
15	3495	0.047	0.058	40	9495	0.019	0.085
16	3735	0.044	0.060	41	9735	0.020	0.086
17	3975	0.042	0.062	42	9975	0.020	0.087
18	4215	0.043	0.063	43	10215	0.018	0.087
19	4455	0.040	0.065	44	10455	0.018	0.088
20	4695	0.037	0.066	45	10695	0.017	0.088
21	4935	0.039	0.068	46	10935	0.017	0.089
22	5175	0.035	0.068	47	11175	0.016	0.090
23	5415	0.035	0.070	48	11415	0.016	0.090
24	5655	0.035	0.071	49	12071	0.015	0.091
25	5895	0.032	0.072				

Reaction conditions: **7a** (0.1 M), DIPAMgCl·LiCl (**4a**, 0.11 M) and *i*-PrMgCl (**1e**, 1.05 M) in THF (0.5 mL) at r.t.

Kinetic profiling of the conversion of **7a-*d*₃** to **8a-*d*₂** by *in-situ* NMR experiments

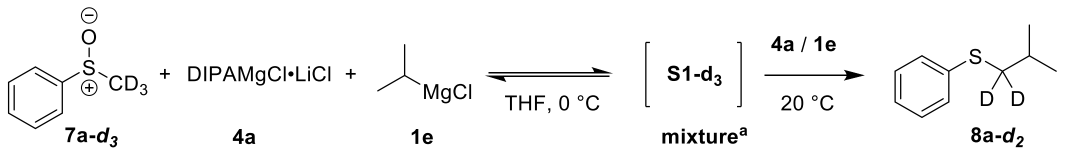

Following general procedure GP-1, deutero-sulfoxide **7a-*d*₃** (7.2 mg, 50 µmol, 1.0 equiv.) in THF (0.40 mL) was mixed with DIPAMgCl·LiCl (**4a**, 73 µL, 0.75 M in THF, 55 µmol, 1.1 equiv.) at 0 °C and a ^{1}H-NMR spectrum was recorded. Grignard reagent **1e** (26 µL, 2.0 M in THF, 53 µmol, 1.05 equiv.) was added at r.t. and reaction progress was monitored by ^{1}H-NMR spectroscopy.

[a]Note: The broad signals denoted as **S1-d₃** are likely an average of various starting material complexes in fast equilibrium in solution. The inherent stability of this mixture prevented further structural characterization by NMR.

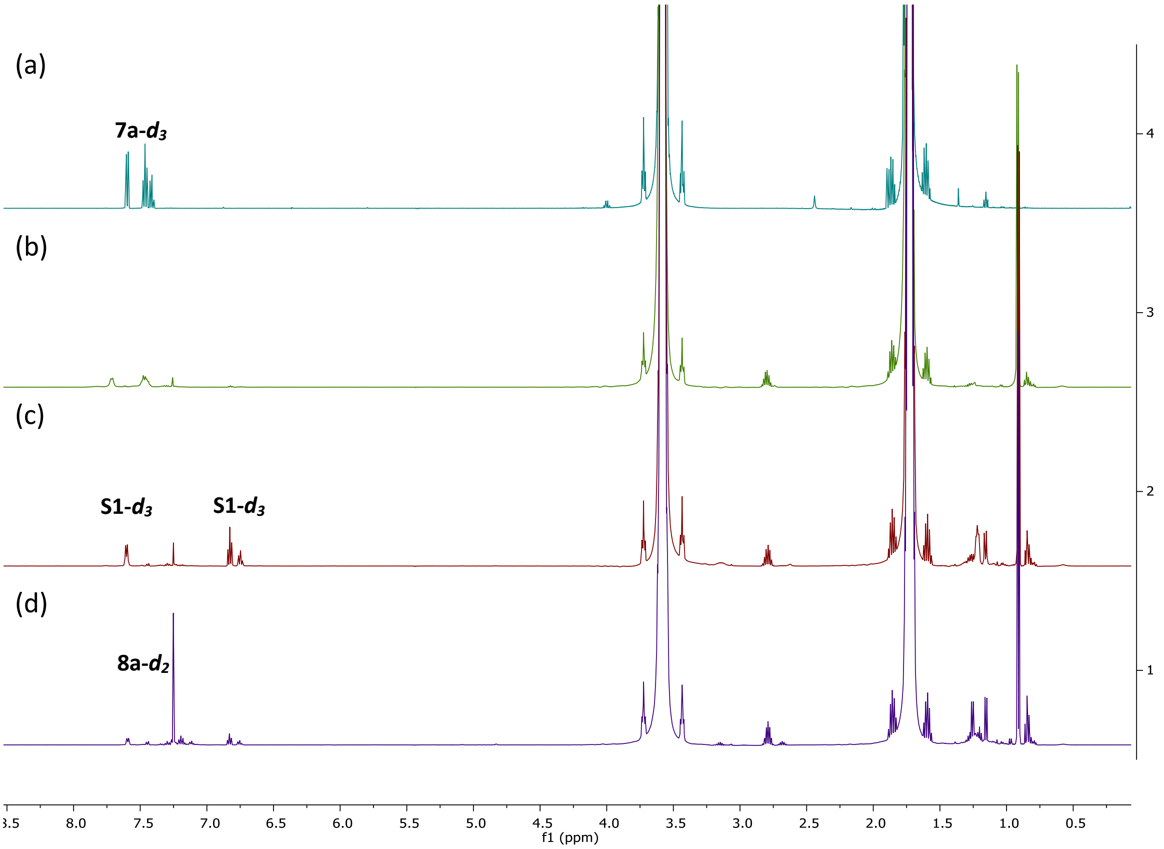

Figure S5*: Overlay of the ^{1}H-NMR spectra of a)* ***7a-d₃*** *in THF b) after addition of DIPAMgCl·LiCl (****4a****), c) after addition of Grignard* ***1e*** *d) after 8 h of reaction time.*

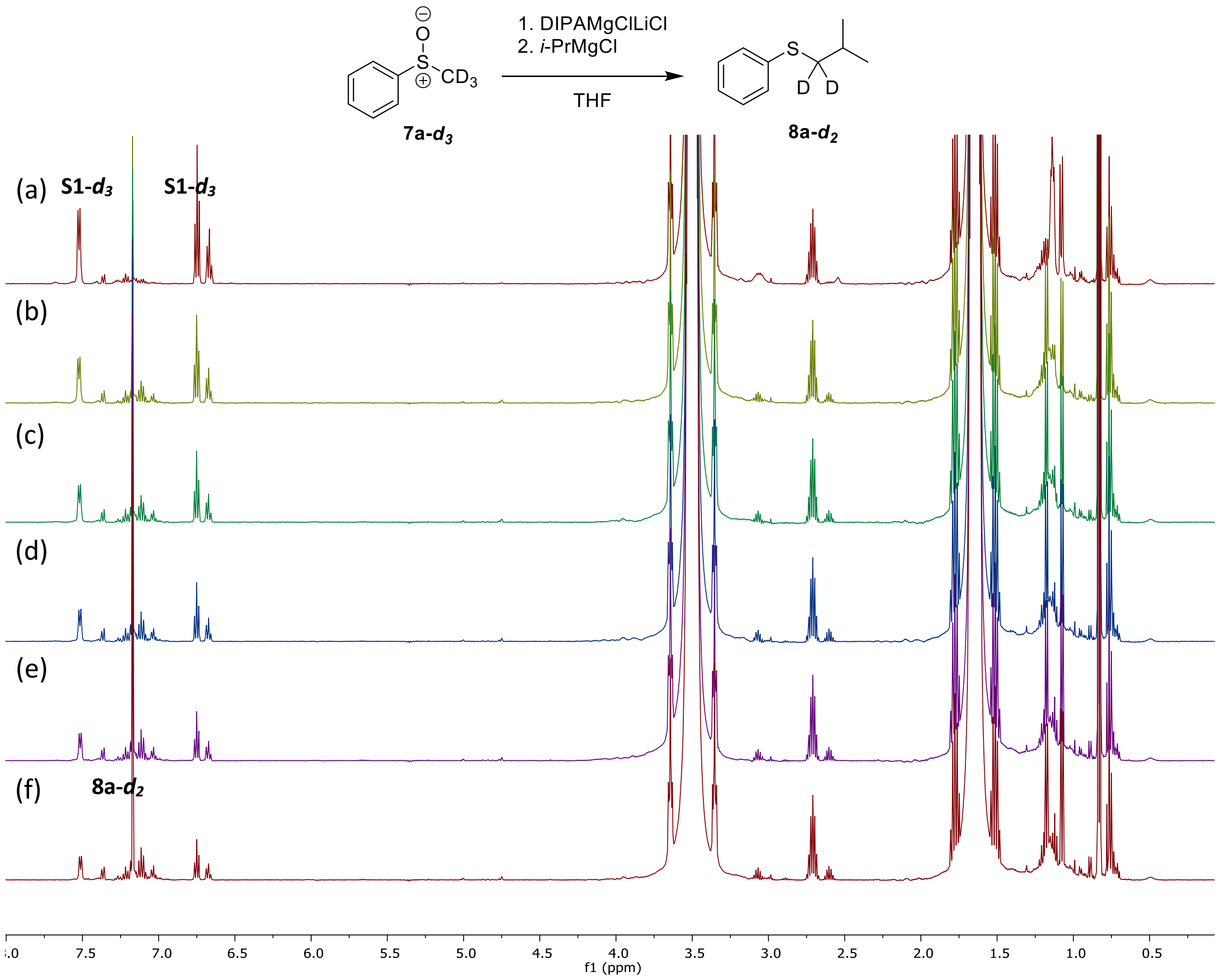

Figure S6. *^{1}H NMR spectra of the conversion of* ***S1-d₃*** *to* ***8a-d₂*** *(a) directly after addition of i-PrMgCl (**1e**), (b) after 97 min reaction time, (c) after 197 min reaction time, (d) after 297 min reaction time, (e) after 6.4 h reaction time, (f) after 8 h reaction time.*

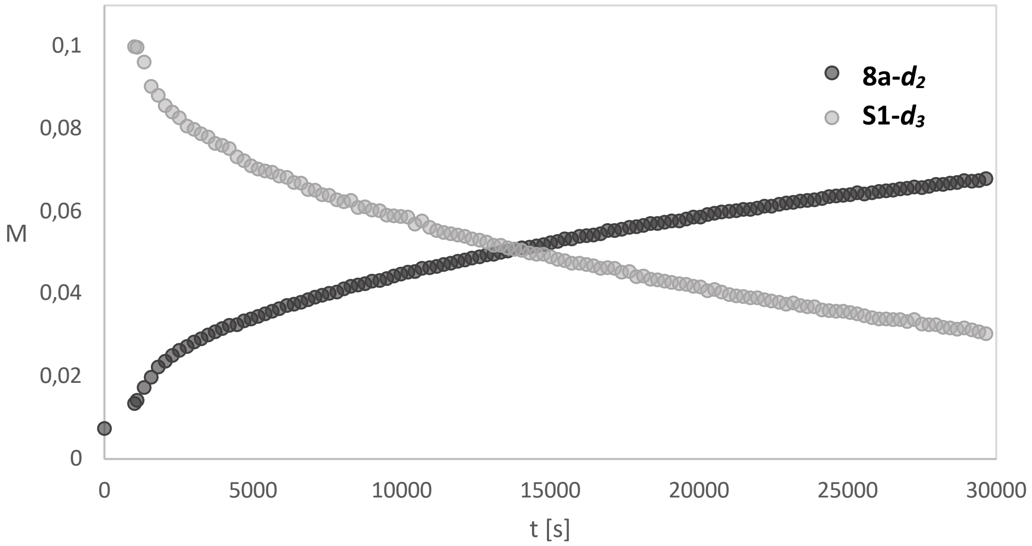

Figure S7*: Kinetic profile of the consumption of intermediate* ***S1-d₃*** *and the formation of product* ***8a-d₂*** *(data see Table S3).*

Table S3*: Data of the reaction profile (see Figure S7)*

Entry	t (s)	[S1-d_3] (M)	[8a-d_2] (M)	Entry	t (s)	[S1-d_3] (M)	[8a-d_2] (M)
1	0	0.100	0.013	61	14229	0.048	0.053
2	69	0.100	0.014	62	14469	0.048	0.053
3	309	0.096	0.017	63	14709	0.047	0.053
4	549	0.090	0.020	64	14949	0.047	0.054
5	789	0.088	0.022	65	15189	0.047	0.054
6	1029	0.086	0.024	66	15429	0.047	0.054
7	1269	0.084	0.025	67	15669	0.046	0.055
8	1509	0.083	0.026	68	15909	0.046	0.055
9	1749	0.081	0.027	69	16149	0.046	0.055
10	1989	0.080	0.028	70	16389	0.045	0.056
11	2229	0.079	0.029	71	16629	0.045	0.056
12	2469	0.078	0.030	72	16869	0.044	0.056
13	2709	0.077	0.031	73	17109	0.044	0.057
14	2949	0.076	0.032	74	17349	0.044	0.057
15	3189	0.075	0.032	75	17589	0.043	0.057
16	3429	0.073	0.033	76	17829	0.043	0.057
17	3669	0.072	0.034	77	18069	0.043	0.058
18	3909	0.071	0.034	78	18309	0.043	0.058
19	4149	0.070	0.035	79	18549	0.042	0.058
20	4389	0.070	0.035	80	18792	0.042	0.059
21	4629	0.070	0.036	81	19032	0.042	0.059
22	4869	0.069	0.036	82	19268	0.041	0.059
23	5109	0.068	0.037	83	19509	0.041	0.060
24	5349	0.067	0.038	84	19749	0.041	0.060
25	5589	0.067	0.038	85	19989	0.040	0.060
26	5829	0.065	0.039	86	20229	0.040	0.060
27	6069	0.065	0.039	87	20469	0.039	0.061
28	6309	0.064	0.040	88	20709	0.039	0.061
29	6549	0.064	0.040	89	20949	0.039	0.061
30	6789	0.063	0.040	90	21189	0.039	0.061
31	7029	0.062	0.041	91	21429	0.038	0.061
32	7269	0.063	0.042	92	21669	0.038	0.062
33	7509	0.061	0.042	93	21909	0.038	0.062
34	7749	0.061	0.042	94	22151	0.038	0.062
35	7989	0.060	0.043	95	22392	0.037	0.063
36	8229	0.060	0.043	96	22629	0.037	0.063
37	8469	0.059	0.044	97	22869	0.037	0.063
38	8709	0.059	0.044	98	23109	0.036	0.063
39	8949	0.059	0.045	99	23349	0.036	0.064
40	9189	0.059	0.045	100	23589	0.036	0.064
41	9429	0.057	0.045	101	23829	0.036	0.064
42	9669	0.058	0.046	102	24069	0.035	0.064
43	9909	0.056	0.046	103	24309	0.035	0.065
44	10149	0.055	0.047	104	24549	0.035	0.064
45	10389	0.055	0.047	105	24789	0.034	0.065
46	10629	0.055	0.048	106	25029	0.034	0.065
47	10869	0.05	0.048	107	25269	0.034	0.065
48	11109	0.054	0.048	108	25509	0.034	0.065
49	11349	0.053	0.049	109	25749	0.034	0.066
50	11589	0.053	0.049	110	25989	0.033	0.066

51	11829	0.053	0.050	111	26229	0.034	0.066
52	12069	0.052	0.050	112	26469	0.033	0.066
53	12309	0.052	0.050	113	26709	0.033	0.066
54	12549	0.051	0.050	114	26949	0.033	0.066
55	12789	0.051	0.051	115	27189	0.032	0.067
56	13029	0.051	0.051	116	27429	0.032	0.067
57	13269	0.050	0.051	117	27669	0.032	0.067
58	13509	0.050	0.052	118	27909	0.032	0.068
59	13749	0.050	0.052	119	28149	0.031	0.067
60	13989	0.049	0.053	120	28389	0.031	0.067

Reaction conditions: **7a-*d*$_3$** (0.1 M), DIPAMgCl·LiCl (**4a**, 0.11 M) and *i*-PrMgCl (**1e**, 1.05 M) in THF (0.5 mL) at r.t.

3. ^{1}H-NMR and ^{13}C-NMR spectra of starting materials

^{1}H-NMR (400 MHz. $CDCl_3$) of **7a**

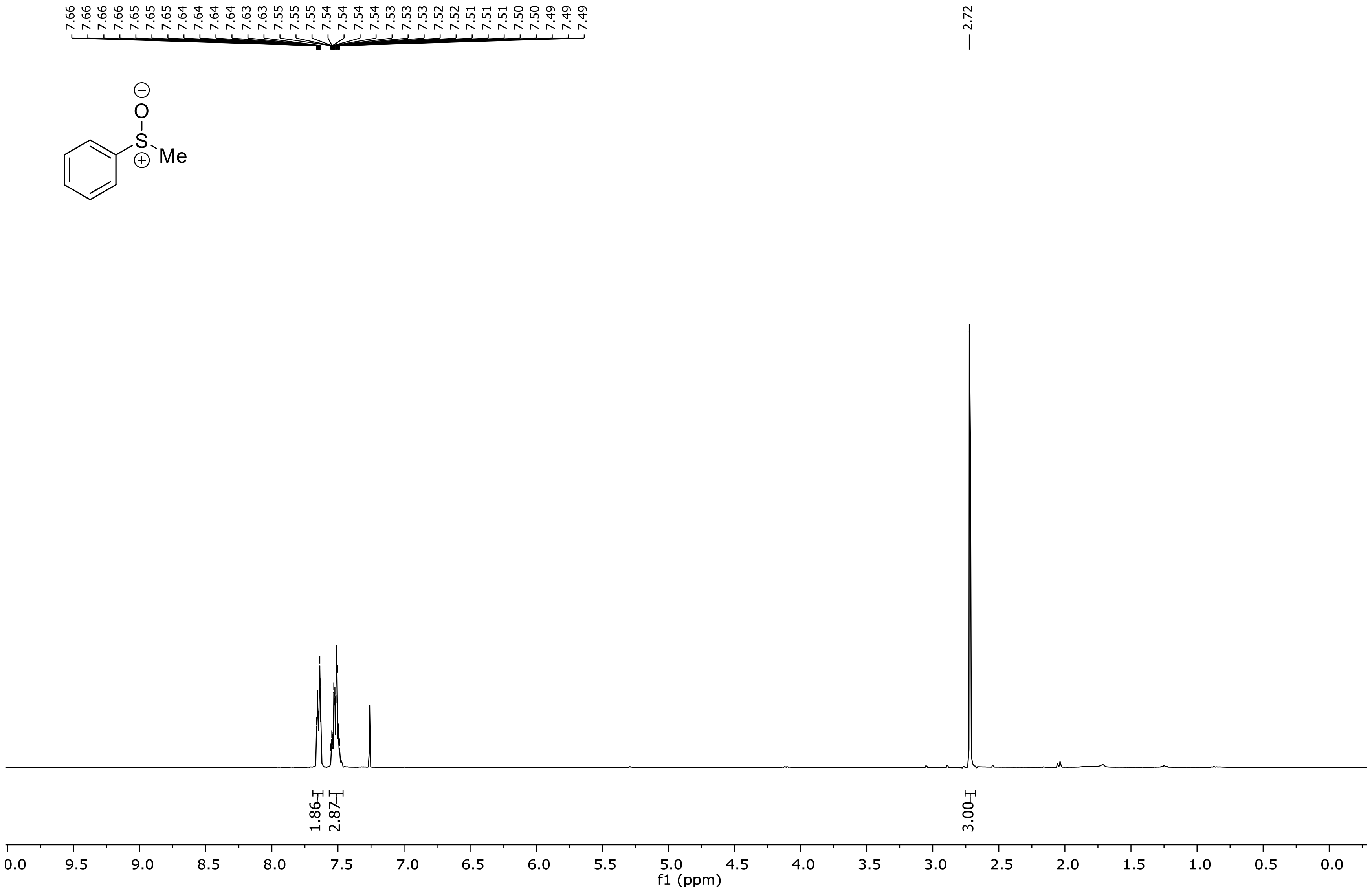

^{1}H-NMR (400 MHz. $CDCl_3$) of **S2**

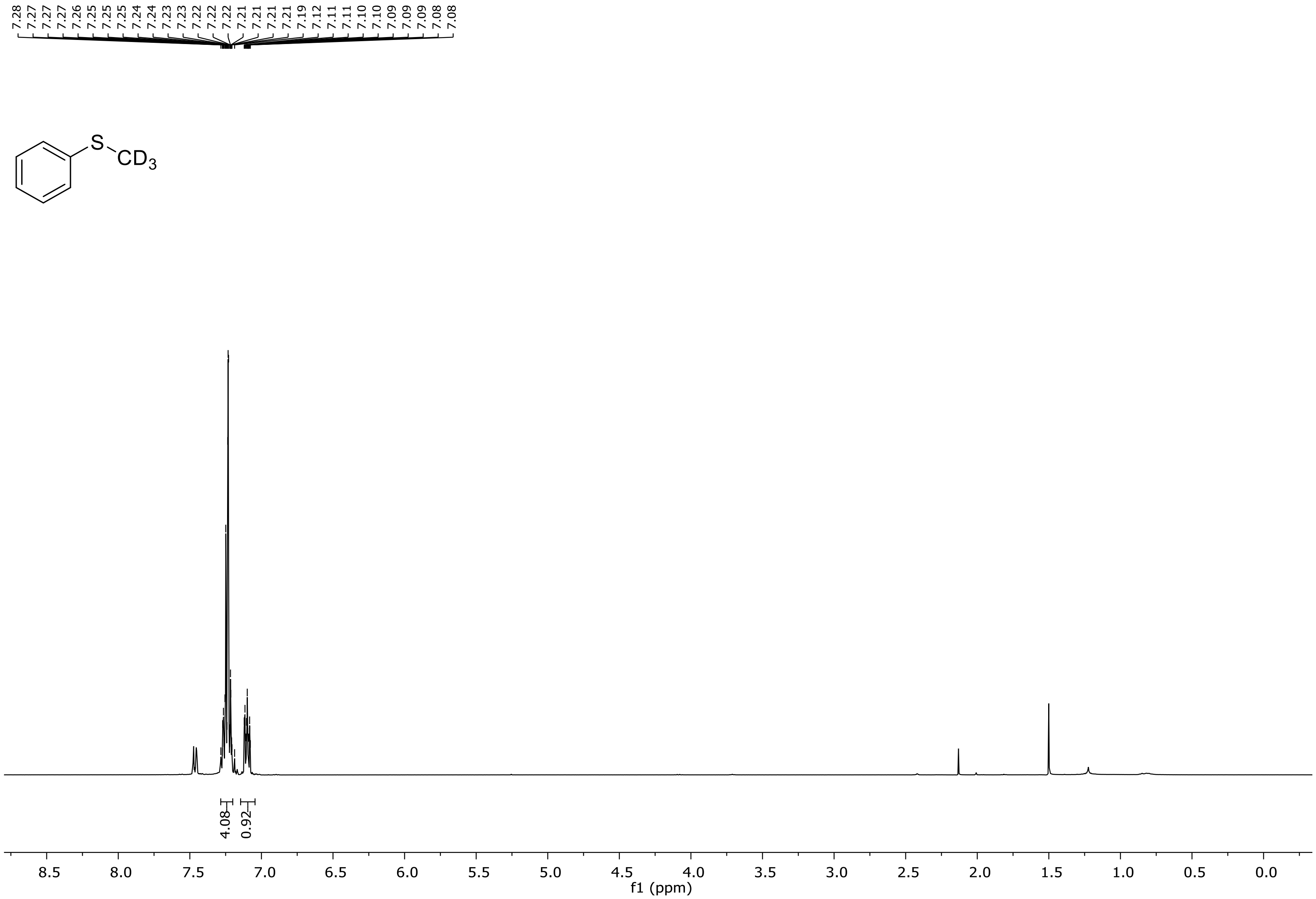

^{13}C-NMR (100 MHz. $CDCl_3$) of **S2**

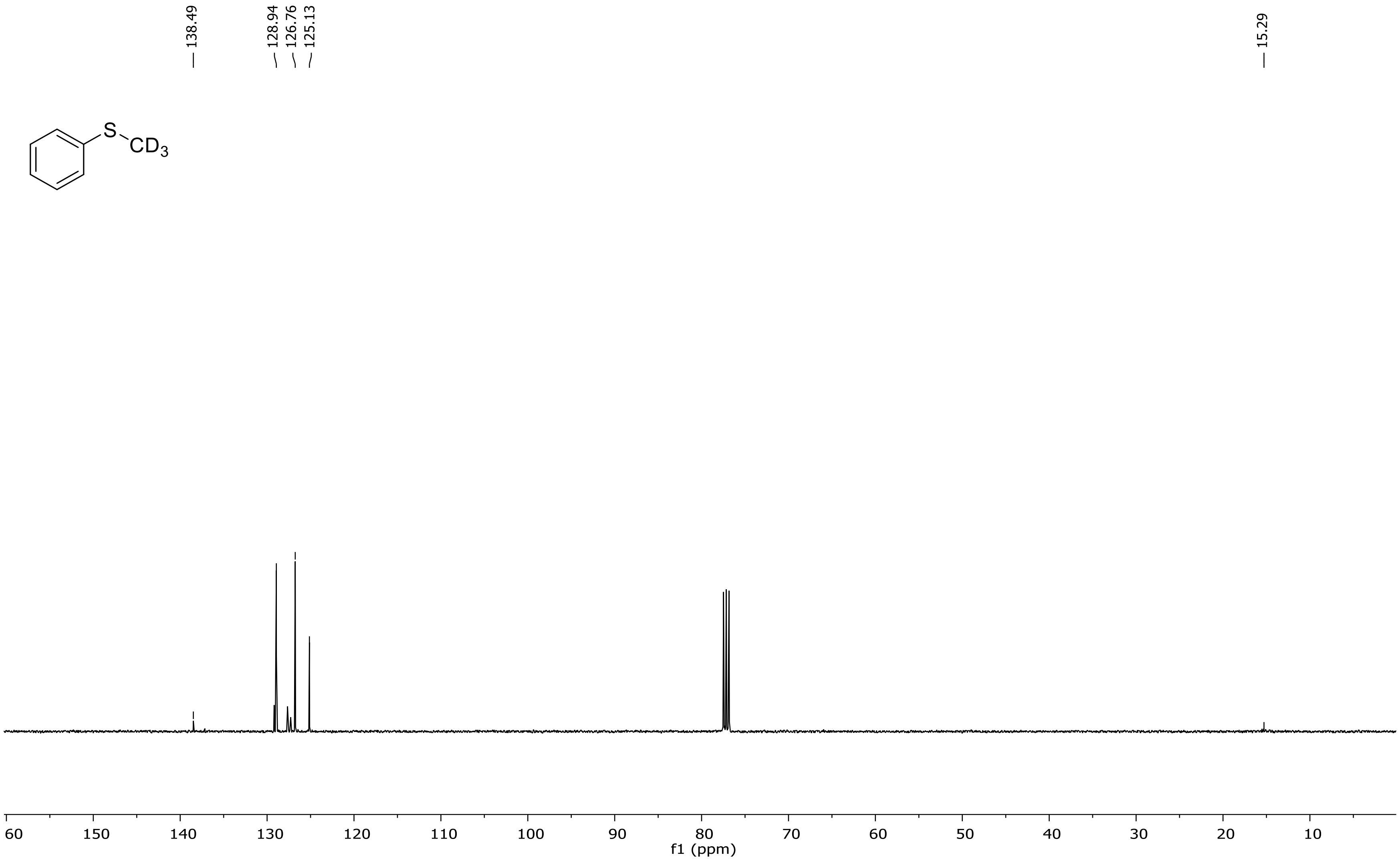

^{1}H-NMR (400 MHz. $CDCl_3$) of **7a-*d*$_3$**

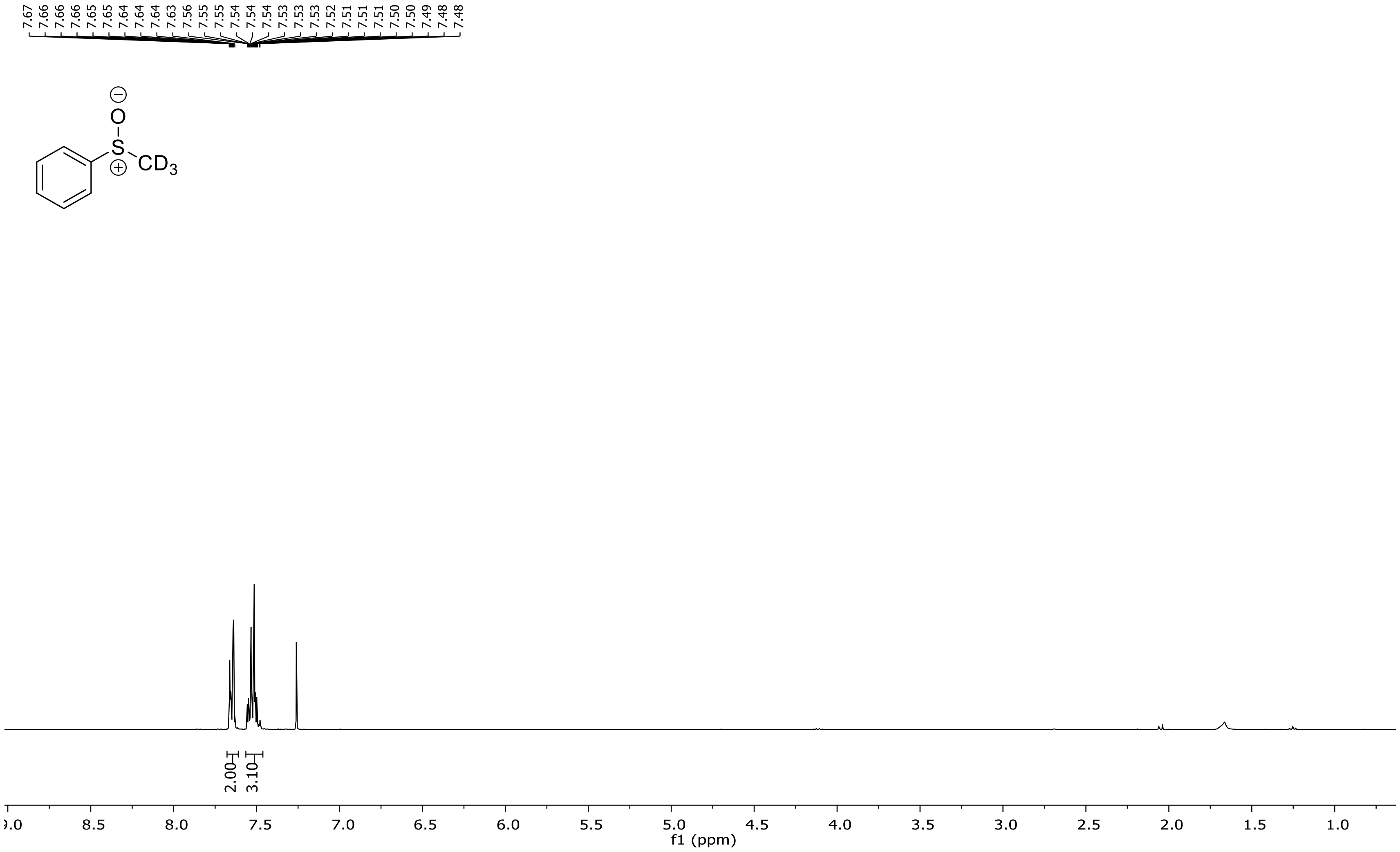

^{13}C-NMR (100 MHz. $CDCl_3$) of ***7a-d₃***

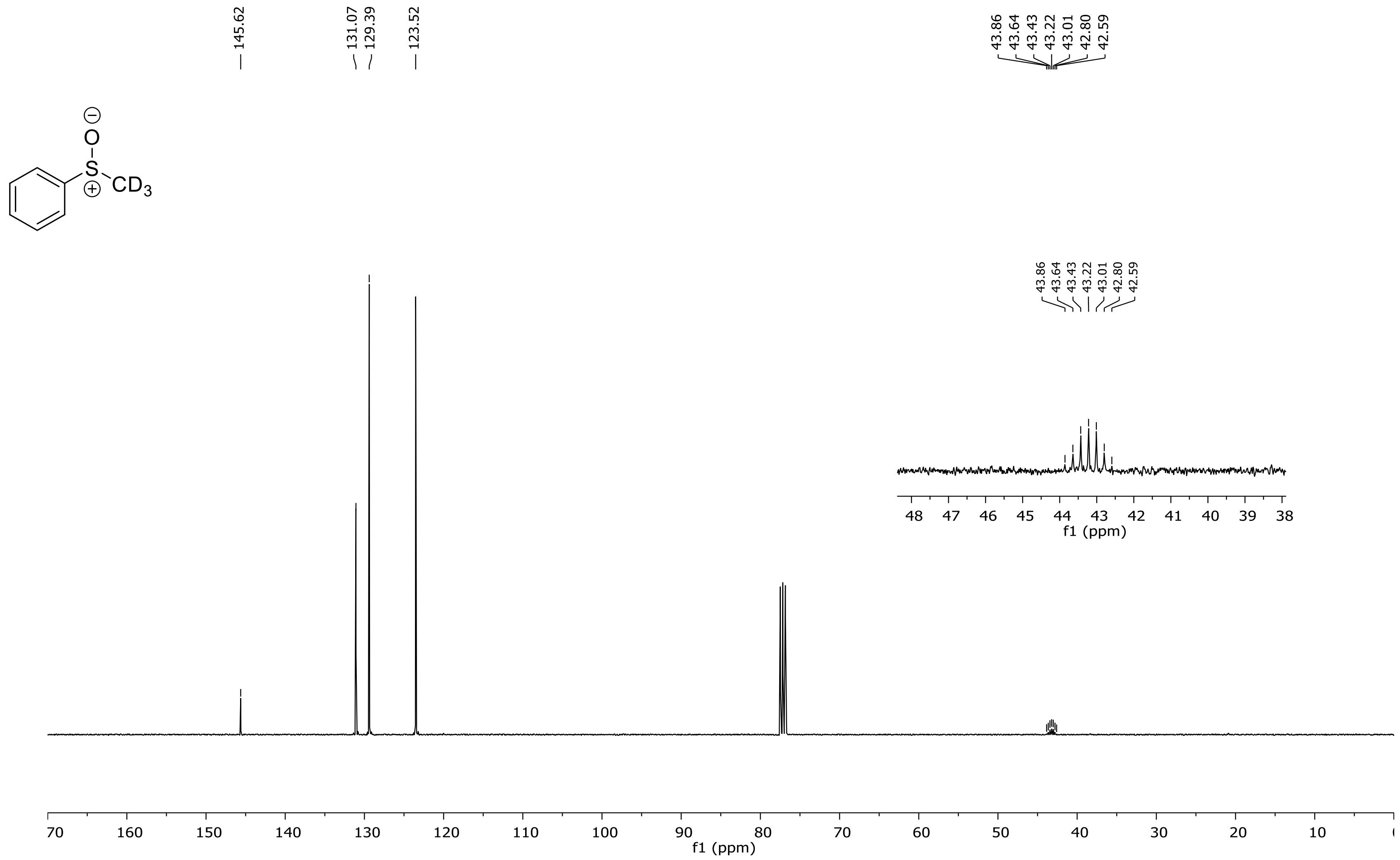

4. References

[**1**] Knochel, P.; Krasovskiy, A. Method of preparing organomagnesium compounds. 2004 EP1582523.

[**2**] Knochel, P.; Krasovskiy, A., Convenient Titration Method for Organometallic Zinc, Magnesium, and Lanthanide Reagents. *Synthesis* **2006,** *2006*, 0890-0891.

[**3**] Krasovskiy, A.; Krasovskaya, V.; Knochel, P., Mixed Mg/Li Amides of the Type $R_2NMgCl{\cdot}LiCl$ as Highly Efficient Bases for the Regioselective Generation of Functionalized Aryl and Heteroaryl Magnesium Compounds. *Angew. Chem., Int. Ed.* **2006,** *45*, 2958-2961.

[**4**] Kakarla, R.; Dulina, R. G.; Hatzenbuhler, N. T.; Hui, Y. W.; Sofia, M. J., Simple and Efficient Method for the Oxidation of Sulfides to Sulfoxides: Application to the Preparation of Glycosyl Sulfoxides. *J. Org. Chem.* **1996,** *61*, 8347-8349.

[**5**] Yoshimura, A.; Nguyen, K. C.; Klasen, S. C.; Saito, A.; Nemykin, V. N.; Zhdankin, V. V., Preparation, structure, and versatile reactivity of pseudocyclic benziodoxole triflate, new hypervalent iodine reagent. *Chem. Commun.* **2015,** *51*, 7835-7838.

[**6**] Baciocchi, E.; Chiappe, C.; Del Giacco, T.; Fasciani, C.; Lanzalunga, O.; Lapi, A.; Melai, B., Reaction of Singlet Oxygen with Thioanisole in Ionic Liquids: a Solvent Induced Mechanistic Dichotomy. *Org. Lett.* **2009,** *11*, 1413-1416.

[**7**] Eberhart, A. J.; Procter, D. J., Nucleophilic ortho-Propargylation of Aryl Sulfoxides: An Interrupted Pummerer/Allenyl Thio-Claisen Rearrangement Sequence. *Angew. Chem., Int. Ed.* **2013,** *52*, 4008-4011.

[**8**] Colas, K.; Martín-Montero, R.; Mendoza, A., Intermolecular Pummerer Coupling with Carbon Nucleophiles in Non-Electrophilic Media. *Angew. Chem., Int. Ed.* **2017,** *56*, 16042-16046.
